# Supplementary material for: The population genomics of archaeological transition in west Iberia: Investigation of ancient substructure using imputation and haplotype-based methods
Source: PLoS Genet. 2017 Jul 27;13(7):e1006852. doi: 10.1371/journal.pgen.1006852 (PMC5531429; doi:10.1371/journal.pgen.1006852)
Supplement: S1 Text — (DOCX) [file pgen.1006852.s001.docx]

# **S1 Text**

# Archaeological information

Rui Martiniano, Lara M Cassidy, Ros Ó'Maoldúin, Russell McLaughlin, Nuno M Silva, Licinio Manco, Daniel Fidalgo, Tania Pereira, Maria J Coelho, Miguel Serra, Joachim Burger, Rui Parreira, Elena Moran, Antonio C Valera, Eduardo Porfirio, Rui Boaventura, Ana M Silva, Daniel G Bradley

## **1.1 Mid Neolithic sites**

### **1.1.1 Lugar do Canto**

Lugar do Canto is a natural cave in which human remains were buried, located near Alcanede, Santarém. It was discovered and excavated in the mid-1970s [[1]](https://paperpile.com/c/3XgBrp/hxrrX). There appear to have been two phases of burial; the first was associated with bone and lithic tools, shell ornaments and polished amphibolite gouges typical of the earlier Neolithic in Portugal, and the second with axes, adzes and wide lithic blades more typical of the middle Neolithic, in total spanning a period from c.4200 to 3500 BC. This approximate dating is supported by one radiocarbon date on human bone, 3880 to 3640 cal BC (@ 95.4%, 4770+40 BP, Beta-276509) (Boaventura et al. 2014). Osteological analysis identified an MNI of 48, that included adults and subadults of varying ages (Rolston in [[1]](https://paperpile.com/c/3XgBrp/hxrrX)). The remains are currently undergoing a reassessment [[2]](https://paperpile.com/c/3XgBrp/OyE9Z). Four petrouses were included in this study, three from adult females (LC41, LC42, LC44) and one from (an adult male?) (LC 45). All present signs of remodeled trauma: LC41 and LC42 of depressed cranial fracture or incomplete trepanation and LC44 and LC45 of complete trepanation.

## **1.2 Late Neolithic/Chalcolithic sites**

1.2.1 Cova da Moura

Cova da Moura is a natural cave, in which human remains were buried, located in the Sizandro River Valley, Estremadura. The site was discovered and excavated in the 1930s [[3]](https://paperpile.com/c/3XgBrp/1ANQZ). Radiocarbon dates obtained from human remains are as follows: 2620 to 2210 cal BC (@ 95.4%, 3950+60 BP, UBAR-536); 3635 to 3372 cal BC (@ 95.4%, 4715+50, UBAR-593) (Silva, 2002). Burial may have continued for as long as 1000 years [[4]](https://paperpile.com/c/3XgBrp/e48mq) but appears to have been concentrated in the Late Neolithic and Copper Age, when exceptionally rich and exotic grave goods (jet, variscite and ivory) were also deposited [[5]](https://paperpile.com/c/3XgBrp/uAWnR). The archaeological deposits were very disturbed and the human bones were disarticulated. Osteological analysis obtained a minimum number of individuals (MNI) of 90, including adults and non-adults [[4]](https://paperpile.com/c/3XgBrp/e48mq). Analysis of stable and strontium isotopes were carried out to establish dietary and mobility patterns [[5,6]](https://paperpile.com/c/3XgBrp/H3Tf9+uAWnR). 4 of the 12 individuals sampled spent at least their early lives somewhere other than the region around Cova da Moura. We analysed CM364 and CM96B.

### **1.2.2 Dolmen de Ansião**

The site of the Dolmen of Ansião is located in the mountainous area of the Alta Estremadura. It was discovered and excavated during road construction in the late nineteenth century. The artefacts [[7]](https://paperpile.com/c/3XgBrp/qS41W) and a single radiocarbon date from a human femur, 3635 to 3372 cal BC (@ 95.4%, 4640±90 BP, Sac- 1559) [[4]](https://paperpile.com/c/3XgBrp/e48mq) suggest a Late Neolithic date. The remains have been moved several times and the assemblage is highly commingled and fragmented. A recent osteological assessment [[4]](https://paperpile.com/c/3XgBrp/e48mq) ascertained an MNI of 37, with 23 adults of both sexes and 14 subadults of various ages. Several injuries including two probable projectile injuries and six depressed cranial fractures were also noted. One (adult) petrous (DA96B) was included in this study.

### **1.2.3 Monte Canelas**

The Monte Canelas necropolis is located in the southern Algarve region of Portugal, only c.700 m north-northwest of the great Alcalar necropolis. There are at least four hypogea at Monte Canelas [[8]](https://paperpile.com/c/3XgBrp/JTs1S). The remains analysed for this project were from Hypogeum 1. Hypogeum 1 consists of two interconnected rock cut chambers, in which, although the majority of remains were commingled, it was possible to identify two phases of deposition, separated by a partial collapse of the chambers [[9]](https://paperpile.com/c/3XgBrp/SjqHs). Osteological analysis identified a total MNI of 171 [[10,11]](https://paperpile.com/c/3XgBrp/RHnRq+N3Cpc). The first phase occurred in the last quarter of the 4th Millennium BC and included a MNI of 147 individuals among which five burials survived ‘in-situ’. They were interred in individual cells, laid in the fetal position with grave goods, including a silex blade, an axe, stone bead necklaces and bone pins [[10,12]](https://paperpile.com/c/3XgBrp/UlMjm+RHnRq). The second phase, or reuse, occurred at the end of the 3rd or beginning of the 2nd Millennium BC. One sample from Hypogeum 1 was included in this study, inhumation 337 (3326-2888 cal BC @ 95.4%,, 4370±60 BP, OxA-5514; 2926-2680 cal BC @ 95.4%, 4250+40 BP, Beta-290366) [[8,10,13]](https://paperpile.com/c/3XgBrp/RHnRq+3LdAP+JTs1S). This was the only ‘in-situ’ burial in the northern chamber and belonged to an elderly female, approximately 60 years of age at death, who was buried with a silex blade, two bone pins and a bead. Osteological analysis noted significant dental wear, degenerative lesions on her vertebrae, signs of scoliosis and a small osteoma in the occipital bone [[10,11]](https://paperpile.com/c/3XgBrp/N3Cpc+RHnRq). Only one sample was sequenced (MC337A).

### **1.2.4 Cabeço da Arruda I**

Cabeço da Arruda I was a probable rock-cut tomb in Torres Vedras, that was partially destroyed upon discovery in the 1930s [[2,14]](https://paperpile.com/c/3XgBrp/iCrjl+OyE9Z). The artefacts suggest the tomb was used between the second half of the fourth and the first part of the third millennia BC. A radiocarbon date obtained from a human long bone returned a date of 3330 to 2885 cal BC (@ 95.4%, 4370 ± 70 BP, Beta – 123363) (Silva, 2002). The human remains were highly commingled and fragmented. Osteological analyses identified an MNI of 19, that included both sexes and adults and non-adults. A depressed fracture, with evidence of healing, was noted on one of the adult skulls [[2,14]](https://paperpile.com/c/3XgBrp/iCrjl+OyE9Z). From this site we obtained sequence data from CA117 and CA122.

## **1.3 Bronze Age sites**

### **1.3.1 Monte do Gato de Cima 3**

Monte do Gato de Cima 3 is a Bronze Age cemetery near Serpa in the Beja district of Alentejo. It is part of a complex of monuments on a small hill (Outeiro Alto 2), that includes ceremonial and funerary remains from the Late Neolithic to the Bronze Age [[15–17]](https://paperpile.com/c/3XgBrp/adNAA+6oit0+JVKFm). One adult male (MG104), from a circular pit burial, was sampled for this project. He was buried in the fetal position with an assemblage typical of the local Bronze Age, an “Atalaia” cup, a bowl and an ox limb. Two overlapping radiocarbon dates suggest a burial date in the late 17th or earlier 16th centuries BC: 1640 to 1430 cal BC (@ 95.4%, 3260+50, Sac 2573) and 1740 to 1545 cal BC (@ 95.4%, 3360±30, Beta-318379) [[18]](https://paperpile.com/c/3XgBrp/03Krt). Osteological analysis noted the unilateral absence of the left mandibular condyle, a probable sign of a traumatic event (Silva et al., in preparation).

### **1.3.2 Monte do Vale do Ouro 2**

Monte do Vale do Ouro 2 is a Bronze Age site near Ferreira do Alentejo, in the Beja district. Excavation at Vale do Ouro 2 uncovered over seventy pits, two of which contained multiple inhumations. One individual (VO10207) was sampled for this project. The sampled individual was a 20-25 year old possible female found underneath two other burials, a 6-10 year old child and another adult, in a circular pit; all were inhumed in the fetal position [[19]](https://paperpile.com/c/3XgBrp/CbPnX). Such inhumations in this region are typically Bronze Age.

### **1.3.3 Torre Velha 3**

Torre Velha 3 is a multi-period site, dating from the Chalcolithic to Late Antiquity, near Serpa, Beja, in the Alentejo region [[20–23]](https://paperpile.com/c/3XgBrp/YfveX+LvsER+V0GS5+mshx2). The remains included 25 hypogea and several circular pits containing Middle Bronze Age burials. Most burials were inhumed in a flexed position and accompanied by grave goods, including pottery, metal artefacts and offerings of meat. Two individuals from Torre Velha 3 were sampled for this project, one from a hypogeum (TV32032) and the other from a circular pit (TV3831). The hypogeum contained two adult male burials, both over 35 years in age. This is unusual in Torre Velha, as it is the only case of two adults of the same sex being buried together. The sampled inhumation was also unusual for being buried in a supine position with flexed limbs; this may be because of his great size and the constricted size of the hypogeum. Osteological analysis identified excessive bone formation on the proximal end of his right femur, possibly signifying a slipped femoral capital epiphysis [[24]](https://paperpile.com/c/3XgBrp/yUk2s). He was buried with a cow limb [[25]](https://paperpile.com/c/3XgBrp/PiV22), a sample of which was dated to between 1750 and 1510 cal BC (@95.4%, 3340±50, Sac-2480). The circular pit contained an adult male and female. They were both buried prone, but with their limbs flexed and their heads to one side so that they faced one another, the male on his right and the female on her left. Although laid out in perfect anatomical position, several bones were missing [[26]](https://paperpile.com/c/3XgBrp/h8mUP) and some bones of both burials were coloured with ochre [[27]](https://paperpile.com/c/3XgBrp/6dCfP). Grave goods included a carinated bowl, the remains of a lithic sickle and another ceramic vessel. The burial is dated to the Bronze Age by the typology of the artefacts [[21]](https://paperpile.com/c/3XgBrp/LvsER).

All dates calibrated in Oxcal v4.2.4 [[28]](https://paperpile.com/c/3XgBrp/Y1lff) and calibrated with IntCal 13 [[29]](https://paperpile.com/c/3XgBrp/X1PVs) at 95.4% probability (2 Sigma). A map with the location of archaeological sites is shown in S1 Fig.

# References

1. [Leitão M, North CT, Norton J, Ferreira O da V, Zbyszewski G. A gruta pré-histórica do Lugar do Canto, Valverde (Alcanede). Arqueologo Português. 1987;5: 37–65.](http://paperpile.com/b/3XgBrp/hxrrX)

2. [Silva AM, Boaventura R, Ferreira MT, Marques R. Skeletal evidence of interpersonal violence from Portuguese Late Neolithic collective burials: an overview. Sticks, stones and broken bones Neolithic violence in a European perspective. 2012; 317–340.](http://paperpile.com/b/3XgBrp/OyE9Z)

3. [Belo R, Trindade L, da Veiga Ferreira O. Gruta da Cova da Moura: Torres Vedras. 1961.](http://paperpile.com/b/3XgBrp/1ANQZ)

4. [Silva AM. Dental anthropology of the Chalcolithic Portuguese population from Cova da Moura (Torres Vedras, Portugal). Permanent lower teeth. Investigaciones en Biodiversidad Humana Santiago de Compostela (Spain): Universidad de Santiago de Compostela. 2000; 367 –374.](http://paperpile.com/b/3XgBrp/e48mq)

5. [Waterman AJ, Peate DW, Silva AM, Thomas JT. In search of homelands: using strontium isotopes to identify biological markers of mobility in late prehistoric Portugal. J Archaeol Sci. 2014/2;42: 119–127.](http://paperpile.com/b/3XgBrp/uAWnR)

6. [Waterman AJ, Tykot RH, Silva AM. Stable Isotope Analysis of Diet-based Social Differentiation at Late Prehistoric Collective Burials in South-Western Portugal. Archaeometry. 2016;58: 131–151.](http://paperpile.com/b/3XgBrp/H3Tf9)

7. [Leisner G. V.(1959): Die Megalithgräber der Iberischen Halbinsel. Der Westen. Madrider Forschungen;](http://paperpile.com/b/3XgBrp/qS41W)

8. [Morán E. El asentamiento prehistórico de Alcalar (Portimão, Portugal): la organización del territorio y el proceso de formación de un estado prístino en el tercer milenio ANE [Internet]. Universidad de Sevilla. 2014. Available:](http://paperpile.com/b/3XgBrp/JTs1S) <http://fondosdigitales.us.es/tesis/tesis/2620/el-asentamiento-prehistorico-de-alcalar-portimao-portugal-la-organizacion-del-territorio-y-el-proceso-de-formacion-de-un-estado-pristino-en-el-tercer-milenio-ne/>

9. [Morán E, Parreira R, Santa-Rita J. Alcalar: monumentos megalíticos. Instituto de Gestão do Património Arquitectónico e Arqueológico; 2007.](http://paperpile.com/b/3XgBrp/SjqHs)

10. [da Silva AMG. O hipogeu de Monte Canelas I (IV-III milénios AC): estudo paleobiológico da população humana exumada. 1996.](http://paperpile.com/b/3XgBrp/RHnRq)

11. [Silva AM, Cunha E. Paleopathological study of the community exhumed from the Hipogeu of Monte Canelas I (Alcalar, Portugal). Actas del V Congreso Nacional de Paleopatologia. 2001. pp. 353–356.](http://paperpile.com/b/3XgBrp/N3Cpc)

12. [Silva AM, Parreira R. O Hipogeu I de Monte Canelas: caracterização antropológica dos enterramentos in situ e das conexões anatómicas. Cascais; 2010.](http://paperpile.com/b/3XgBrp/UlMjm)

13. [Silva AM, Parreira R. O Hipogeu I de Monte Canelas: caracterização antropológica dos enterramentos in situ e das conexões anatómicas. Cascais; 2010.](http://paperpile.com/b/3XgBrp/3LdAP)

14. [Silva AM. A Neolithic skull lesion probably caused by an arrowhead. Antropologia portuguesa. Universidade de Coimbra, Instituto de Antropologia; 2002;19: 139–144.](http://paperpile.com/b/3XgBrp/iCrjl)

15. [Valera AC, Filipe V. Outeiro Alto 2 (Brinches, Serpa): Nota preliminar sobre um espaço funerário e de socialização do Neolítico Final à Idade do Bronze. Apontamentos de Arqueologia e Património. 2010;5: 49–56.](http://paperpile.com/b/3XgBrp/adNAA)

16. [Valera AC, Filipe V. Outeiro Alto 2 (Brinches, Serpa): nota preliminar sobre um espaço funerário e de socialização do Neolítico Final a Idade do Bronze. Apontamentos de Arqueologia e Património. 2012;8: 9–42.](http://paperpile.com/b/3XgBrp/6oit0)

17. [Valera AC, Filipe V, Cabaço N. O recinto de fosso do Outeiro Alto 2 (Brinches, Serpa). Apontamentos de Arqueologia e Património. 2013;9: 21–35.](http://paperpile.com/b/3XgBrp/JVKFm)

18. [Valera AC, Godinho R, Calvo E, Berraquero JM, Filipe V, Santos H. Um mundo em negativo: fossos, fossas e hipogeus entre o Neolítico Final e a Idade do Bronze na margem esquerda do Guadiana (Brinches, Serpa). Actas do IV Colóquio Arqueológico de Alqueva. 2010; Available:](http://paperpile.com/b/3XgBrp/03Krt) <http://www.academia.edu/download/30650436/Microsoft_Word_-_Um_mundo_em_negativo.pdf>

19. [Pereira TF da E. Enterramentos em fossa no distrito de Beja: práticas funerárias e estudo dos vestígios osteológicos da Pré-história recente. estudogeral.sib.uc.pt; 2014; Available:](http://paperpile.com/b/3XgBrp/CbPnX) <https://estudogeral.sib.uc.pt/handle/10316/27222>

20. [Alves C, Costeira C, Estrela S, Porfirio E, Serra M, Soares AMM, et al. Hipogeos funerarios del Bronce Pleno en Torre Velha 3 (Serpa, Portugal). Zephyrvs. 2011;66: 133–153.](http://paperpile.com/b/3XgBrp/YfveX)

21. [Alves C, Costeira C, Estrela S, Porfírio E, Serra M. Torre Velha 3 (Serpa): Dados Preliminares. Al-Madan; 2012.](http://paperpile.com/b/3XgBrp/LvsER)

22. [Alves C, Costeira C, Estrela S, Serra M, Porfirio E. Necrópole Tardo-Antiga da Torre Velha 3, Serpa (Baixo Alentejo, Portugal). VI Encuentro de Arqueología del Suroeste Peninsular. 2013. pp. 1929–1966.](http://paperpile.com/b/3XgBrp/V0GS5)

23. [Valério P, Monge Soares AM, Fátima Araújo M, Silva RJC, Porfírio E, Serra M. Arsenical copper and bronze in Middle Bronze Age burial sites of southern Portugal: the first bronzes in Southwestern Iberia. J Archaeol Sci. 2014/2;42: 68–80.](http://paperpile.com/b/3XgBrp/mshx2)

24. [Fidalgo DFF. Contextos funerários e estudo antropológico dos restos ósseos humanos dos hipogeus de Torre Velha 3 (São Salvador, Serpa): Uma aproximação ao estudo das comunidades humanas do Bronze do Sudoeste. 2014; Available:](http://paperpile.com/b/3XgBrp/yUk2s) <https://eg.sib.uc.pt/handle/10316/27862>

25. [Porfírio EMB, Serra MAP. Rituais funerários e comensalidade no Bronze do Sudoeste da Península Ibérica: novos dados a partir de uma intervenção arqueológica no sítio da Torre Velha 3 (Serpa). Estudos do Quaternário/Quaternary Studies. 2014; Available:](http://paperpile.com/b/3XgBrp/PiV22) <http://www.apeq.pt/ojs/index.php/apeq/article/view/93>

26. [Coelho MJ. Do Calcolítico à Idade do Bronze: Contextos funerários e análise paleobiológica de restos osteológicos humanos exumados das fossas de Torre Velha 3 (São Salvador, Serpa). Dissertação de Mestrado em Biologia e Evolução Humanas. 2015.](http://paperpile.com/b/3XgBrp/h8mUP)

27. [Porfírio E. Arquitectura e práticas funerárias da Idade do Bronze no concelho de Serpa: o caso de Torre Velha 3. Antrope. 2014;série monográfica, n.^o^ 1: 250–268.](http://paperpile.com/b/3XgBrp/6dCfP)

28. [Ramsey CB, Scott M, van der Plicht H. Calibration for archaeological and environmental terrestrial samples in the time range 26-50 ka cal BP. Radiocarbon. Cambridge University Press; 2013;55: 2021–2027.](http://paperpile.com/b/3XgBrp/Y1lff)

29. [Reimer PJ, Bard E, Bayliss A, Beck JW, Blackwell PG, Bronk Ramsey C, et al. IntCal13 and Marine13 radiocarbon age calibration curves 0-50,000 years cal BP. University of Arizona; 2013; Available:](http://paperpile.com/b/3XgBrp/X1PVs) <http://researchcommons.waikato.ac.nz/handle/10289/8955>

**S1 Fig - Map and geographical locations of the archaeological locations of the samples sequenced in the present study.**
