# Supplementary material for: The population genomics of archaeological transition in west Iberia: Investigation of ancient substructure using imputation and haplotype-based methods
Source: PLoS Genet. 2017 Jul 27;13(7):e1006852. doi: 10.1371/journal.pgen.1006852 (PMC5531429; doi:10.1371/journal.pgen.1006852)
Supplement: S2 Text — (DOCX) [file pgen.1006852.s002.docx]

# **S2 Text**

# Ancient DNA analysis

Rui Martiniano, Lara M Cassidy, Ros Ó'Maoldúin, Russell McLaughlin, Nuno M Silva, Licinio Manco, Daniel Fidalgo, Tania Pereira, Maria J Coelho, Miguel Serra, Joachim Burger, Rui Parreira, Elena Moran, Antonio C Valera, Eduardo Porfirio, Rui Boaventura, Ana M Silva, Daniel G Bradley

## **2.1 Sample preparation**

### **2.1.1 DNA sampling and extractions**

Archaeological samples were processed at the Ancient DNA lab, Smurfit Institute, Trinity College Dublin (Ireland), in clean-room facilities, were modern DNA has not been amplified. For the present study, we selected 14 samples from Portugal, from which 4 date to Middle Neolithic, 6 to the Final Neolithic and 4 to the Bronze Age (Table 1, S1 Fig). All samples consisted of the petrous portion of the temporal bone, because it typically allows for higher amounts of endogenous DNA to be retrieved, in comparison with other bone elements [[1]](https://paperpile.com/c/VSR6eP/YS2Wu). First, we decontaminated bone surfaces by UV irradiation for approximately 20 minutes and removed the superficial layer of the bone with a drill bit. Next, we cut the densest part of each petrous bone and extracted 100-150 mg of bone powder per sample using a silica-based method as described in [[2]](https://paperpile.com/c/VSR6eP/jfxFB) with modifications [[3]](https://paperpile.com/c/VSR6eP/ErxBt). After 24 hours of incubation at 55 °C and 37 °C, we keep the supernatant and re-extracted undigested pellets for another 48 hrs. Finally, we purified the second supernatant with Minelute columns (Qiagen MinElute PCR Purification Kit, Qiagen, Hilden, Germany) and eluted in 50 ul of dH2O. We included blank controls in all experiments: DNA extraction, library preparation and PCRs, in order to check for possible contamination.

### **2.1.2 Library preparation and amplification**

We incorporated ancient DNA fragments into Next-generation sequencing libraries using the Meyer and Kircher protocol [[4]](https://paperpile.com/c/VSR6eP/gHMuZ) and modified as in [[1,5]](https://paperpile.com/c/VSR6eP/8FaGu+YS2Wu). Library amplification was done for 12-14 cycles with AccuPrime Pfx Polymerase (Invitrogen) and a distinct indexing oligo for each ancient sample. We quantified PCR products with Quant-iT dsDNA HS Assay kit (Invitrogen, Oregon, USA) and TapeStation 2200 (Agilent Technologies, Santa Clara, CA), averaged measurements obtained with both instruments and pooled for sequencing.

### **2.1.3 Illumina Sequencing**

We initially screened NGS libraries with an Illumina MiSeq (50 cycle kit, single-end reads mode, Institute of Molecular Medicine (IMM), Trinity College Dublin), pooled with PhiX control at 1% concentration. The best preserved samples, as indicated by the proportion of endogenous human DNA, were then re-amplified with 2-4 indexing oligos per sample, pooled and sequenced with Illumina HiSeq 2000 (100 cycle kit, single-end reads mode; Macrogen).

## **2.2 Next-Generation Sequencing Reads Processing and Filtration**

### **2.2.1 NGS reads processing, alignment and filtration**

We trimmed NGS reads with Cutadapt v. 1.3 [[6]](https://paperpile.com/c/VSR6eP/qOo1z) and kept reads with a minimum length of 30 base pairs, and allowing for a minimum overlap of 1 bp between the read and the adapter (cutadapt -a AGATCGGAAGAGCACACGTCTGAACTCCAGTCAC -O 1 -m 30). Next, we aligned NGS reads to the human reference genome (UCSC hg19) with Burrows-Wheeler Aligner v.0.7.5a-r405 (BWA) [[7]](https://paperpile.com/c/VSR6eP/45QDW), which we filtered by base quality 15 and disabled seed length as recommended for mapping ancient DNA sequences [[8]](https://paperpile.com/c/VSR6eP/9GAjS). We used SAMtools v.0.1.19-44428cd [[9]](https://paperpile.com/c/VSR6eP/EyBVh) to removed duplicates and to select reads mapped with quality ⋝ 30. We estimated genome coverage using qualimap [[10]](https://paperpile.com/c/VSR6eP/N2A8G) (Table 1).

## **2.3 Sex determination**

We determined sex of the ancient samples from Portugal using a Ry_compute [[11]](https://paperpile.com/c/VSR6eP/zfOpl) which calculates the ratio of X to Y chromosome reads and outputs the most likely sex. In total we determined 5 samples to be female and 9 samples to be male (S2 Fig).

## **2.4 Contamination estimates and verification of authenticity**

### **2.4.1 Estimation of Deamination rates**

To determine the extent of deamination patterns, we analysed our reads with mapDamage 2.0 [[12]](https://paperpile.com/c/VSR6eP/AJy6a) (S3 Fig). The values obtained for cytosine deamination are consistent with what is expected for ancient samples and this provides evidence for the authenticity of the ancient DNA sequences obtained.

### **2.4.2 Estimation of contamination rates based on the X-chromosome**

The haploid state of the X-chromosome in male individuals allows estimating the level of contamination. To do this, we used ANGSD [[13]](https://paperpile.com/c/VSR6eP/POvwK) which implements a previously published method [[14]](https://paperpile.com/c/VSR6eP/qOkrv). This method assumes that if males are haploid for the X-chromosome, then mismatches at polymorphic sites imply either we are in the presence of contamination or sequencing errors. In order to account for the latter, polymorphic sites and adjacent bases are screened for mismatches, because sequencing errors should be observed at identical frequencies across the genome. X-chromosome contamination was estimated to be on average 1.3% (0-2.3%) (S1 and S2 Table)

### **2.4.3 Mitochondrial DNA contamination estimates**

First, we aligned reads to the Cambridge Reference sequence (ref), filtering for PCR duplicates and removing potential contamination with mapdamage (ref). We uploaded aligned mitochondrial reads to the server (ref) where we obtained information about mtDNA haplogroup assignment and heteroplasmy. The average heteroplasmy proportion across the 14 samples was 0.044 (0-0.127) and consisted of C to T and G to A modifications that were not removed (S3 Table). Considering that X-chromosome contamination was very low (~2%), and that the heteroplasmy identified consisted of potential deamination changes, we conclude that there is no substantial contamination affecting our results.

## **2.5 Uniparental markers**

### **2.5.1 Y-chromosome lineage determination**

In order to investigate Y-chromosomal haplogroups in ancient samples, we used Y-haplo [[15]](https://paperpile.com/c/VSR6eP/C3kJ) (<https://github.com/23andMe/yhaplo>). First, we called genotypes with GATK Unifiedgenotyper, with the parameter ploidy set to 1, restricting our analysis to sites within callable regions [[16]](https://paperpile.com/c/VSR6eP/yLiE). Calls were filtered by minimum quality 30 with vcftools and male lineages identified with callHaplogroups.py -c option, which makes use of curated Y-chromosome markers from the International Society of Genetic Genealogy (ISOGG) 2016 (https://isogg.org/tree/2016/index16.html). Derived alleles are shown in S4 Table.

In broad terms, the Late Neolithic/Chalcolithic samples presented 3 Y-chromosome lineages belonging to I2a1 and one G2a2a1. Both G and I haplogroups were identified in previously sampled Neolithic remains from Spain [[17]](https://paperpile.com/c/VSR6eP/szI8M) and the I2a1a lineage was found in a Chalcolithic Spanish [[18]](https://paperpile.com/c/VSR6eP/T9aIb). Haplogroup G has been associated with the Neolithic Demic diffusion [[19]](https://paperpile.com/c/VSR6eP/9XsqG), with G2a2 being identified in Anatolian aDNA samples [[18,20]](https://paperpile.com/c/VSR6eP/T9aIb+DAc0m), Otzi [[21]](https://paperpile.com/c/VSR6eP/9Nxr) and German LBK [[22]](https://paperpile.com/c/VSR6eP/z9Qq). I lineages were already present in european HGs from Scandinavia and Hungary, such as Motala [[23]](https://paperpile.com/c/VSR6eP/c1u0) and KO1 [[1]](https://paperpile.com/c/VSR6eP/YS2Wu), respectively, and several Neolithic samples throughout Europe, which is consistent with admixture between hunter-gatherer and early farmer groups and the persistence of pre-Neolithic lineages in Neolithic contexts.

In the Bronze Age samples we observe a switch from G and I lineages to the R1b-M269 clades associated with the Steppe migrations into Europe by the Yamnaya pastoralists [[22]](https://paperpile.com/c/VSR6eP/z9Qq). It is important to distinguish that while ancient Central [[18,22,24]](https://paperpile.com/c/VSR6eP/z9Qq+T9aIb+6H49) and Northwestern Europeans [[25]](https://paperpile.com/c/VSR6eP/sBQZ) typically present R1b1a2a1-L51 derived, of numerous western European distribution, the Yamnaya belong to R1b1a2a2-CTS1078/Z2105, of more Eastern distribution. Of the 3 R1b-M269 lineages observed in the Portuguese Bronze Age, 2 individuals had sufficient coverage to identify the derived allele at the P312 marker which is frequent in present-day Western Europeans and also has been determined in 2 Bell Beaker samples in Central Europe [[22,24]](https://paperpile.com/c/VSR6eP/z9Qq+6H49).

In present-day Portuguese, the frequency of the main lineages here identified are R1b-M269 59.9%, G 5.5% and I 4.9 % [[26]](https://paperpile.com/c/VSR6eP/fYKFB), suggesting that migrations occurring after the Neolithic, which transported the majority of R1b-derived individuals, considerably shaped the Y-chromosomal landscape in Portugal. It was not possible to identify the allelic state at DF27 marker which defines the most frequent clade in Iberia [[27]](https://paperpile.com/c/VSR6eP/O53sI).

We note that a Late Neolithic/Chalcolithic sample (MC337A) for which we generated low coverage data (0.05X) presented a derived allele at marker L1063, which defines lineage R1b1a2. While it is possible that R1b1a2 lineages existed at low frequencies in Iberia before the Bronze Age, we do not have sufficient evidence that supports this. First, the L1063 marker is a C to T mutation, and therefore it could consist of aDNA damage; second, there is only one read covering this SNP; third, there are no more markers which confirm that this sample belongs to the R branch; last, the fact that MC337A presents the ancestral allele at marker M9, which defines haplogroup K, does not support this samples’ assignment to R1b1a2, since the K haplogroup is upstream of R. The Portuguese Bronze Age samples show the derived allele at K-M9, which is consistent with these individuals belonging to R1b-M269 lineages.

Taking these results into consideration, it is possible that MC337A belonged to R1b1a2, as one other sample from EN Spain carried the basal R1b1, however the present evidence (above) is not sufficient to demonstrate this convincingly. Furthermore, results presented by other studies [[18,22,24]](https://paperpile.com/c/VSR6eP/z9Qq+6H49+T9aIb), show that G and I haplogroups, not R1b1a2, were present in the the vast majority of Neolithic samples in Iberia and the new data we present in this study is concordant with this notion, and our finding of a Bronze Age arrival of R1b-P312 lineages to Iberian Peninsula is not affected by this possibility.

We note that our results were produced with low coverage data, and therefore urge caution in their interpretation.

### **2.5.2 mtDNA haplogroup identification**

Next-Generation Sequencing reads were aligned to the revised Cambridge Reference Sequence (rCRS; NC_012920.1) [[28]](https://paperpile.com/c/VSR6eP/LKFiR), using BWA filtering for base (q ≥ 30) and mapping (q ≥ 30) quality and duplicate reads were removed. We then used SAMtools [[9]](https://paperpile.com/c/VSR6eP/EyBVh) to obtain the mtDNA consensus for each ancient sample, which was then uploaded to HaploFind (<https://haplofind.unibo.it>; [[29]](https://paperpile.com/c/VSR6eP/TY3pq)), where mitochondrial haplogroup defining positions were obtained. We estimated mtDNA contamination by calculating the proportion of mismatches at haplotype defining sites (S3 Table). Additionally, we also uploaded alignments in the bam format to mtDNA-Server (<http://mtdna-server.uibk.ac.at/>; beta version) where we confirmed mtDNA haplogroups and estimated coverage (Table 1, S3 Table).

# References

1. [Gamba C, Jones ER, Teasdale MD, McLaughlin RL, Gonzalez-Fortes G, Mattiangeli V, et al. Genome flux and stasis in a five millennium transect of European prehistory. Nat Commun. 2014;5: 5257.](http://paperpile.com/b/VSR6eP/YS2Wu)

2. [Yang DY, Eng B, Waye JS, Dudar JC, Saunders SR. Technical note: improved DNA extraction from ancient bones using silica-based spin columns. Am J Phys Anthropol. 1998;105: 539–543.](http://paperpile.com/b/VSR6eP/jfxFB)

3. [MacHugh DE, Edwards CJ, Bailey JF, Bancroft DR, Bradley DG. The Extraction and Analysis of Ancient DNA From Bone and Teeth: a Survey of Current Methodologies. Anc Biomol. 2000;3: 81.](http://paperpile.com/b/VSR6eP/ErxBt)

4. [Meyer M, Kircher M. Illumina sequencing library preparation for highly multiplexed target capture and sequencing. Cold Spring Harb Protoc. 2010;2010: db.prot5448.](http://paperpile.com/b/VSR6eP/gHMuZ)

5. [Martiniano R, Coelho C, Ferreira MT, Neves MJ, Pinhasi R, Bradley DG. Genetic evidence of African slavery at the beginning of the trans-Atlantic slave trade. Sci Rep. 2014;4: 5994.](http://paperpile.com/b/VSR6eP/8FaGu)

6. [Martin M. Cutadapt removes adapter sequences from high-throughput sequencing reads [Internet]. EMBnet.journal. 2011. pp. 10–12. doi:](http://paperpile.com/b/VSR6eP/qOo1z)[http://journal.embnet.org/index.php/embnetjournal/article/view/200](http://dx.doi.org/http://journal.embnet.org/index.php/embnetjournal/article/view/200)

7. [Li H, Durbin R. Fast and accurate short read alignment with Burrows-Wheeler transform. Bioinformatics. 2009;25: 1754–1760.](http://paperpile.com/b/VSR6eP/45QDW)

8. [Schubert M, Ginolhac A, Lindgreen S, Thompson JF, Al-Rasheid KAS, Willerslev E, et al. Improving ancient DNA read mapping against modern reference genomes. BMC Genomics. 2012;13: 178.](http://paperpile.com/b/VSR6eP/9GAjS)

9. [Li H, Handsaker B, Wysoker A, Fennell T, Ruan J, Homer N, et al. The Sequence Alignment / Map (SAM) Format and SAMtools 1000 Genome Project Data Processing Subgroup. Bioinformatics. 2009;25: 2078–2079.](http://paperpile.com/b/VSR6eP/EyBVh)

10. [García-Alcalde F, Okonechnikov K, Carbonell J, Cruz LM, Götz S, Tarazona S, et al. Qualimap: evaluating next-generation sequencing alignment data. Bioinformatics. 2012;28: 2678–2679.](http://paperpile.com/b/VSR6eP/N2A8G)

11. [Skoglund P, Storå J, Götherström A, Jakobsson M. Accurate sex identification of ancient human remains using DNA shotgun sequencing. J Archaeol Sci. 2013;40: 4477–4482.](http://paperpile.com/b/VSR6eP/zfOpl)

12. [Jónsson H, Ginolhac A, Schubert M, Johnson PLF, Orlando L. mapDamage2.0: fast approximate Bayesian estimates of ancient DNA damage parameters. Bioinformatics. 2013;29: 1682–1684.](http://paperpile.com/b/VSR6eP/AJy6a)

13. [Korneliussen T, Albrechtsen A, Nielsen R. ANGSD: Analysis of Next Generation Sequencing Data. BMC Bioinformatics. 2014;15: 356.](http://paperpile.com/b/VSR6eP/POvwK)

14. [Rasmussen M, Guo X, Wang Y, Lohmueller KE, Rasmussen S, Albrechtsen A, et al. An Aboriginal Australian Genome Reveals Separate Human Dispersals into Asia. Science. 2011;334 : 94–98.](http://paperpile.com/b/VSR6eP/qOkrv)

15. [David Poznik G. Identifying Y-chromosome haplogroups in arbitrarily large samples of sequenced or genotyped men [Internet]. bioRxiv. 2016. p. 088716. doi:](http://paperpile.com/b/VSR6eP/C3kJ)[10.1101/088716](http://dx.doi.org/10.1101/088716)

16. [Poznik GD, Henn BM, Yee M-C, Sliwerska E, Euskirchen GM, Lin AA, et al. Sequencing Y chromosomes resolves discrepancy in time to common ancestor of males versus females. Science. 2013;341: 562–565.](http://paperpile.com/b/VSR6eP/yLiE)

17. [Lacan M, Keyser C, Ricaut F-X, Brucato N, Duranthon F, Guilaine J, et al. Ancient DNA reveals male diffusion through the Neolithic Mediterranean route. Proc Natl Acad Sci U S A. 2011;108: 9788–9791.](http://paperpile.com/b/VSR6eP/szI8M)

18. [Mathieson I, Lazaridis I, Rohland N, Mallick S, Patterson N, Roodenberg SA, et al. Genome-wide patterns of selection in 230 ancient Eurasians. Nature. 2015;528: 499–503.](http://paperpile.com/b/VSR6eP/T9aIb)

19. [Cinnioglu C, Cengiz C, Roy K, Toomas K, Ersi K, Sevil A, et al. Excavating Y-chromosome haplotype strata in Anatolia. Hum Genet. 2004;114: 127–148.](http://paperpile.com/b/VSR6eP/9XsqG)

20. [Hofmanová Z, Kreutzer S, Hellenthal G, Sell C, Diekmann Y, del Molino DD, et al. Early farmers from across Europe directly descended from Neolithic Aegeans [Internet]. bioRxiv. 2015. p. 032763. doi:](http://paperpile.com/b/VSR6eP/DAc0m)[10.1101/032763](http://dx.doi.org/10.1101/032763)

21. [Keller A, Graefen A, Ball M, Matzas M, Boisguerin V, Maixner F, et al. New insights into the Tyrolean Iceman’s origin and phenotype as inferred by whole-genome sequencing. Nat Commun. 2012;3: 698.](http://paperpile.com/b/VSR6eP/9Nxr)

22. [Haak W, Lazaridis I, Patterson N, Rohland N, Mallick S, Llamas B, et al. Massive migration from the steppe was a source for Indo-European languages in Europe. Nature. 2015; doi:](http://paperpile.com/b/VSR6eP/z9Qq)[10.1038/nature14317](http://dx.doi.org/10.1038/nature14317)

23. [Skoglund P, Malmström H, Omrak A, Raghavan M, Valdiosera C, Günther T, et al. Genomic diversity and admixture differs for Stone-Age Scandinavian foragers and farmers. Science. 2014;344: 747–750.](http://paperpile.com/b/VSR6eP/c1u0)

24. [Allentoft ME, Sikora M, Sjögren K-G, Rasmussen S, Rasmussen M, Stenderup J, et al. Population genomics of Bronze Age Eurasia. Nature. 2015;522: 167–172.](http://paperpile.com/b/VSR6eP/6H49)

25. [Cassidy LM, Martiniano R, Murphy EM, Teasdale MD, Mallory J, Hartwell B, et al. Neolithic and Bronze Age migration to Ireland and establishment of the insular Atlantic genome. Proc Natl Acad Sci U S A. 2016;113: 368–373.](http://paperpile.com/b/VSR6eP/sBQZ)

26. [Beleza S, Gusmão L, Lopes A, Alves C, Gomes I, Giouzeli M, et al. Micro-phylogeographic and demographic history of Portuguese male lineages. Ann Hum Genet. 2006;70: 181–194.](http://paperpile.com/b/VSR6eP/fYKFB)

27. [Valverde L, Illescas MJ, Villaescusa P, Gotor AM, García A, Cardoso S, et al. New clues to the evolutionary history of the main European paternal lineage M269: dissection of the Y-SNP S116 in Atlantic Europe and Iberia. Eur J Hum Genet. 2016;24: 437–441.](http://paperpile.com/b/VSR6eP/O53sI)

28. [Andrews RM, Kubacka I, Chinnery PF, Lightowlers RN, Turnbull DM, Howell N. Reanalysis and revision of the Cambridge reference sequence for human mitochondrial DNA. Nat Genet. 1999;23: 147.](http://paperpile.com/b/VSR6eP/LKFiR)

29. [van Oven M, Kayser M. Updated comprehensive phylogenetic tree of global human mitochondrial DNA variation. Hum Mutat. 2009;30: E386–94.](http://paperpile.com/b/VSR6eP/TY3pq)

**S1 Table - X-chromosome contamination estimated with ANGSD (Korneliussen et al. 2014) and based on a previously published method (Rasmussen et al. 2011).**

**S2 Table - X-chromosome contamination based on the number of mismatches at X-chromosome SNPs and adjacent sites.**

**S3 Table - mtDNA lineages and contamination estimates based on mismatches at haplotype defining sites.**

**S4 Table - Y-chromosome lineages determined in the ancient Portuguese samples.**

**S2 Fig - Sex Determination using Ry_compute.**

**S3 Fig - Post-mortem misincorporations in ancient samples.**
