# Supplementary material for: The population genomics of archaeological transition in west Iberia: Investigation of ancient substructure using imputation and haplotype-based methods
Source: PLoS Genet. 2017 Jul 27;13(7):e1006852. doi: 10.1371/journal.pgen.1006852 (PMC5531429; doi:10.1371/journal.pgen.1006852)
Supplement: S3 Text — (DOCX) [file pgen.1006852.s003.docx]

# **S3 Text**

# Comparison of ancient samples with other ancient and modern datasets using genotype data

##

Rui Martiniano, Lara M Cassidy, Ros Ó'Maoldúin, Russell McLaughlin, Nuno M Silva, Licinio Manco, Daniel Fidalgo, Tania Pereira, Maria J Coelho, Miguel Serra, Joachim Burger, Rui Parreira, Elena Moran, Antonio C Valera, Eduardo Porfirio, Rui Boaventura, Ana M Silva, Daniel G Bradley

## **3.1 Processing of Published Ancient Data**

Ancient data from eighteen previous studies [[1–17]](https://paperpile.com/c/eE9Kz0/SoDG2+hkpTl+vlmsf+ukrYh+vExsN+BeW0A+bFOdA+r7RXC+1WmDN+qCXSk+9R3vv+ViZRo+TaPqv+szPoC+cFxT7+KlP4S+FdPIi) were combined with the Portuguese samples from the current study for use in population genetic analyses.

Briefly, FASTQ files from [[1,4,11]](https://paperpile.com/c/eE9Kz0/SoDG2+ukrYh+9R3vv) and BAM files from [[3,5,9,15,18]](https://paperpile.com/c/eE9Kz0/vlmsf+vExsN+1WmDN+mTwt6+cFxT7) were aligned/realigned using BWA [[19]](https://paperpile.com/c/eE9Kz0/qFUyb) to the human reference genome (hg19/GRCh37), with the mitochondrial genome replaced by the revised Cambridge reference sequence (rCRS). Mapped reads were sorted, filtered for mapping quality 30, and had PCR duplicates removed using samtools [[20]](https://paperpile.com/c/eE9Kz0/0KvlW). Read groups were added using Picard Tools v1.101 (<http://broadinstitute.github.io/picard/>). RealignerTargetCreator and IndelRealigner tools from GenomeAnalysisTK v2.4-7 [[21]](https://paperpile.com/c/eE9Kz0/4Bpzo) were used to realign indels. Reads were also softclipped, meaning that the base qualities of two base pairs at both the 5’ and 3’ ends of reads were reduced to a PHRED score of 2. The processing of data from [[2,12–14,17]](https://paperpile.com/c/eE9Kz0/hkpTl+ViZRo+TaPqv+szPoC+FdPIi), is the same as that described in their respective methods sections, with the exception that the data from [[12–14]](https://paperpile.com/c/eE9Kz0/ViZRo+TaPqv+szPoC) was not subject to mapDamage rescaling and data from [[17]](https://paperpile.com/c/eE9Kz0/FdPIi) was softclipped.

The processed whole genome data from these thirteen studies, alongside the fourteen Portuguese samples (processed without mapDamage rescaling), was used for imputation analysis detailed in S5 Text. Only published samples with a coverage above 0.85X were considered for imputation. For diploid genotype calling in high coverage ancient samples used for imputation validation, non-UDG treated samples were rescaled using mapDamage [[22]](https://paperpile.com/c/eE9Kz0/pwICj).

## **3.2. Haploid genotype calling and merging with the Affymetrix Human Origins dataset**

For haploid genotype calling all non-UDG treated samples (including samples comprised of a large portion of non-UDG treated libraries) had the base quality scores of likely deaminated positions in reads rescaled using mapDamage 2.0 [[22]](https://paperpile.com/c/eE9Kz0/pwICj).

The pileup tool in GATK v2.4 [[23]](https://paperpile.com/c/eE9Kz0/NFpu0) was used to determine sample base calls for 594,896 autosomal positions in the Human Origins dataset described in [[5]](https://paperpile.com/c/eE9Kz0/vExsN) for ancient samples from [[1–5,9,11–15,17,18]](https://paperpile.com/c/eE9Kz0/SoDG2+hkpTl+vlmsf+ukrYh+vExsN+1WmDN+mTwt6+9R3vv+ViZRo+TaPqv+szPoC+cFxT7+FdPIi) and the fourteen Portuguese individuals. A minimum base quality of 30 was required and sites with three or more different bases present were removed. A single base call was then picked at random from each site and duplicated to create a diploid homozygous genotype at that position. This strategy was used, regardless of coverage, for all ancient genomes.

These pseudo-diploid genotype calls were then merged using PLINK v1.90 [[24]](https://paperpile.com/c/eE9Kz0/lHkUS) with corresponding genotypes from 160 ancient individuals, taken from the targeted 1240k SNP capture dataset published in [[16]](https://paperpile.com/c/eE9Kz0/KlP4S), including the genotypes of 67 individuals initially published in [[10]](https://paperpile.com/c/eE9Kz0/qCXSk) and 10 low coverage individuals (0.10- 1.24X) initially published in [[2]](https://paperpile.com/c/eE9Kz0/hkpTl). The dataset also included genotypes for three palaeolithic hunter gatherers sequenced in separate studies [[6–8]](https://paperpile.com/c/eE9Kz0/BeW0A+bFOdA+r7RXC). Published samples with less than 100,000 confident genotype calls were subsequently removed from the merged dataset. Three outlying Russian Iron age samples were also removed. This left 224 ancient samples for use in population genetic analysis. The ancient dataset was then merged with 1941 modern individuals from the Affymetrix Human Origins dataset described in [[5]](https://paperpile.com/c/eE9Kz0/vExsN), for use in PCA (S3.3) and ADMIXTURE analysis (Section 3.4)

## **3.3 Principal Component Analysis**

The genetic affinities of the fourteen Portuguese samples were examined in the context of modern and ancient Eurasian populations using principal component analysis (PCA). Smartpca version 10210 from EIGENSOFT [[25,26]](https://paperpile.com/c/eE9Kz0/ijC3f+6aqFA) was used to perform PCA on a subset of West Eurasian populations (604 individuals) from the Human Origins dataset [[5]](https://paperpile.com/c/eE9Kz0/vExsN), based on approximately 600,000 SNPs. The genetic variation of 224 ancient Eurasian genomes, including the 14 Portuguese individuals, was then projected onto the modern PCA (lsqproject: YES option). The results are displayed in S4 Fig.

## **3.4 ADMIXTURE Analysis.**

We applied the model-based clustering approach of the program ADMIXTURE v.1.23 [[27]](https://paperpile.com/c/eE9Kz0/gI9RZ) to estimate ancestry components in 10 of the Portuguese samples, alongside 1941 modern humans from populations worldwide [[5]](https://paperpile.com/c/eE9Kz0/vExsN) and 166 published ancient individuals. Only ancient samples with a minimum of 250,000 secure genotype calls were included. The dataset was also filtered for related individuals. SNPs with a genotyping rate below 97.5% were removed. The remaining variants were then pruned for sites in strong linkage disequilibrium. This was done using the --indep-pairwise option in PLINK v1.90 with the parameters 200, 25 and 0.4, resulting in a final 219,982 SNPs for analysis.

ADMIXTURE was run for all ancestral population numbers from K=2 to K=15, with cross-validation enabled (--cv flag). This analysis was replicated 40 times over. The results for the best of these replicates for each value of K, i.e. those with the highest loglikelihood, were extremely similar to those presented in [[13]](https://paperpile.com/c/eE9Kz0/TaPqv). Here, we obtained the lowest median CV error for K=10. Fig 4 displays the admixture profiles for all ancient individuals and selected modern Eurasian individuals for the best replicate of K=10, ordered by geographical region and time depth. The Tyrolean iceman, Oetzi, has been excluded from this figure, due to a lack of individuals from the same region, as well as two Russian Iron Age individuals without a securely dated context. Four Palaeolithic genomes included in the analysis are also not shown.

**S4 Fig - Principal component analysis of 604 modern West Eurasians onto which variation from 224 ancient genomes has been projected.**

The analysis is based on approximately 600,000 SNP positions. Moderns samples from the Human Origins dataset are represented in greyscale, with the exception of modern Iberians shown in green. Ancient samples are coloured by time depth and shaped according to geographic region. Ancient individuals from Portugal are outlined in red.

# References

1. [Keller A, et al. (2012) New insights into the Tyrolean Iceman’s origin and phenotype as inferred by whole-genome sequencing. *Nat Commun* 3:698.](http://paperpile.com/b/eE9Kz0/SoDG2)

2. [Gamba C, et al. (2014) Genome flux and stasis in a five millennium transect of European prehistory. *Nat Commun* 5:5257.](http://paperpile.com/b/eE9Kz0/hkpTl)

3. [Skoglund P, et al. (2014) Genomic diversity and admixture differs for Stone-Age Scandinavian foragers and farmers. *Science* 344(6185):747–750.](http://paperpile.com/b/eE9Kz0/vlmsf)

4. [Olalde I, et al. (2014) Derived immune and ancestral pigmentation alleles in a 7,000-year-old Mesolithic European. *Nature* 507(7491):225–228.](http://paperpile.com/b/eE9Kz0/ukrYh)

5. [Lazaridis I, et al. (2014) Ancient human genomes suggest three ancestral populations for present-day Europeans. *Nature* 513(7518):409–413.](http://paperpile.com/b/eE9Kz0/vExsN)

6. [Seguin-Orlando A, et al. (2014) Paleogenomics. Genomic structure in Europeans dating back at least 36,200 years. *Science* 346(6213):1113–1118.](http://paperpile.com/b/eE9Kz0/BeW0A)

7. [Fu Q, et al. (2014) Genome sequence of a 45,000-year-old modern human from western Siberia. *Nature* 514(7523):445–449.](http://paperpile.com/b/eE9Kz0/bFOdA)

8. [Raghavan M, et al. (2014) The genetic prehistory of the New World Arctic. *Science* 345 (6200 ). doi:](http://paperpile.com/b/eE9Kz0/r7RXC)[10.1126/science.1255832](http://dx.doi.org/10.1126/science.1255832)[.](http://paperpile.com/b/eE9Kz0/r7RXC)

9. [Allentoft ME, et al. (2015) Population genomics of Bronze Age Eurasia. *Nature* 522(7555):167–172.](http://paperpile.com/b/eE9Kz0/1WmDN)

10. [Haak W, et al. (2015) Massive migration from the steppe was a source for Indo-European languages in Europe. *Nature*. doi:](http://paperpile.com/b/eE9Kz0/qCXSk)[10.1038/nature14317](http://dx.doi.org/10.1038/nature14317)[.](http://paperpile.com/b/eE9Kz0/qCXSk)

11. [Olalde I, et al. (2015) A Common Genetic Origin for Early Farmers from Mediterranean Cardial and Central European LBK Cultures. *Mol Biol Evol* 32(12):3132–3142.](http://paperpile.com/b/eE9Kz0/9R3vv)

12. [Jones ER, et al. (2015) Upper Palaeolithic genomes reveal deep roots of modern Eurasians. *Nat Commun* 6:8912.](http://paperpile.com/b/eE9Kz0/ViZRo)

13. [Cassidy LM, et al. (2016) Neolithic and Bronze Age migration to Ireland and establishment of the insular Atlantic genome. *Proc Natl Acad Sci U S A* 113(2):368–373.](http://paperpile.com/b/eE9Kz0/TaPqv)

14. [Martiniano R, et al. (2016) Genomic signals of migration and continuity in Britain before the Anglo-Saxons. *Nat Commun* 7:10326.](http://paperpile.com/b/eE9Kz0/szPoC)

15. [Schiffels S, et al. (2016) Iron Age and Anglo-Saxon genomes from East England reveal British migration history. *Nat Commun* 7:10408.](http://paperpile.com/b/eE9Kz0/cFxT7)

16. [Mathieson I, et al. (2015) *Eight thousand years of natural selection in Europe* doi:](http://paperpile.com/b/eE9Kz0/KlP4S)[10.1101/016477](http://dx.doi.org/10.1101/016477)[.](http://paperpile.com/b/eE9Kz0/KlP4S)

17. [Hofmanová Z, et al. (2015) Early farmers from across Europe directly descended from Neolithic Aegeans. *bioRxiv*:032763.](http://paperpile.com/b/eE9Kz0/FdPIi)

18. [Günther T, et al. (2015) Ancient genomes link early farmers from Atapuerca in Spain to modern-day Basques. *Proc Natl Acad Sci U S A* 112(38):11917–11922.](http://paperpile.com/b/eE9Kz0/mTwt6)

19. [Li H, Durbin R (2009) Fast and accurate short read alignment with Burrows-Wheeler transform. *Bioinformatics* 25(14):1754–1760.](http://paperpile.com/b/eE9Kz0/qFUyb)

20. [Li H, et al. (2009) The Sequence Alignment / Map (SAM) Format and SAMtools 1000 Genome Project Data Processing Subgroup. *Bioinformatics* 25:2078–2079.](http://paperpile.com/b/eE9Kz0/0KvlW)

21. [McKenna A, et al. (2010) The Genome Analysis Toolkit: a MapReduce framework for analyzing next-generation DNA sequencing data. *Genome Res* 20(9):1297–1303.](http://paperpile.com/b/eE9Kz0/4Bpzo)

22. [Jónsson H, Ginolhac A, Schubert M, Johnson PLF, Orlando L (2013) mapDamage2.0: fast approximate Bayesian estimates of ancient DNA damage parameters. *Bioinformatics* 29(13):1682–1684.](http://paperpile.com/b/eE9Kz0/pwICj)

23. [McKenna A, et al. (2010) The Genome Analysis Toolkit: a MapReduce framework for analyzing next-generation DNA sequencing data. *Genome Res* 20(9):1297–1303.](http://paperpile.com/b/eE9Kz0/NFpu0)

24. [Chang CC, et al. (2015) Second-generation PLINK: rising to the challenge of larger and richer datasets. *Gigascience* 4:7.](http://paperpile.com/b/eE9Kz0/lHkUS)

25. [Patterson N, Price AL, Reich D (2006) Population structure and eigenanalysis. *PLoS Genet* 2:2074–2093.](http://paperpile.com/b/eE9Kz0/ijC3f)

26. [Price AL, et al. (2006) Principal components analysis corrects for stratification in genome-wide association studies. *Nat Genet* 38(8):904–909.](http://paperpile.com/b/eE9Kz0/6aqFA)

27. [Alexander DH, Novembre J, Lange K (2009) Fast model-based estimation of ancestry in unrelated individuals. *Genome Res* 19(9):1655–1664.](http://paperpile.com/b/eE9Kz0/gI9RZ)
