# Supplementary material for: The population genomics of archaeological transition in west Iberia: Investigation of ancient substructure using imputation and haplotype-based methods
Source: PLoS Genet. 2017 Jul 27;13(7):e1006852. doi: 10.1371/journal.pgen.1006852 (PMC5531429; doi:10.1371/journal.pgen.1006852)
Supplement: S4 Text — (DOCX) [file pgen.1006852.s004.docx]

# **S4 Text**

# Exploring ancient Iberian affinities through F- and D-statistics.

Rui Martiniano, Lara M Cassidy, Ros Ó'Maoldúin, Russell McLaughlin, Nuno M Silva, Licinio Manco, Daniel Fidalgo, Tania Pereira, Maria J Coelho, Miguel Serra, Joachim Burger, Rui Parreira, Elena Moran, Antonio C Valera, Eduardo Porfirio, Rui Boaventura, Ana M Silva, Daniel G Bradley

## **4.1 Dataset Preparation**

To further dissect the genetic affinities of Portuguese Neolithic and Bronze Age individuals, tests of admixture in the form of D-statistics were carried out. Postmortem damage in aDNA, as well as the differing strategies used by labs to address it, are both potential confounders of estimates of gene flow between ancient individuals. For this reason we chose to perform D-statistics on transversion sites alone. We used biallelic autosomal transversion positions from the 1000 Genomes Phase 3 release, with a minor allele frequency filter above 1% in European populations imposed. Haploid genotype calling in whole genome ancient data was carried out as described in Supplementary S3.2. This resulted in 2,598,013 positions for use in statistical tests. Four Mbuti individuals from the SGDP dataset [[1]](https://paperpile.com/c/s9HOea/9vraO) were used as an outgroup in all tests. Z-Scores above 1.5 are considered of interest, and those above 3 significant. Of note, samples from the targeted 1240k SNP capture dataset published in [[2]](https://paperpile.com/c/s9HOea/F1WW) were excluded from analysis. Results are shown in S5 and S6 Table.

### **4.1.1 Relationships to other Neolithic groups**

The earliest Neolithic individuals sampled from Iberia belong to the Cardial Ware tradition, the main culture of the Early Mediterranean Neolithic. These individuals have been previously shown to share more affinity with Neolithic Anatolians and Greeks than those from Germany and Hungary [[3]](https://paperpile.com/c/s9HOea/KSyt). We expand on these results here, exploring the affinities of a Cardial individual from the Early Neolithic (EN) of northeast Spain [[4]](https://paperpile.com/c/s9HOea/3e6d) with individuals from Middle Neolithic Hungary, Early and Late Neolithic Greece, the Neolithic in Anatolia and the Neolithic LBK culture of Central Europe. Tests were constructed in the form - D(Mbuti, Cardial EN; NE1, NE2). Although no significant values were obtained, we find the Cardial individual shows somewhat higher affinity to the Greek Early Neolithic in every test combination performed (Z=1.358-2.245). This observation would support archaeological evidence suggesting a westward Neolithic expansion across the Mediterranean from Greece associated with Cardium pottery, independent from the inland movement into Hungary and Germany via the Danubian valley [[5]](https://paperpile.com/c/s9HOea/v0dLs).

To explore the affinities of later Neolithic populations in Portugal, a similar set of tests were carried out in the form - D(Mbuti, Portuguese MN/LNCA; NE1, NE2). Here, we observe a preference for both the Greek and Cardial EN over all other Early Neolithic groups (Z=2.533-3.991), further suggesting a Mediterranean origin for the Atlantic Neolithic communities of Iberia.

### **4.1.2 Mesolithic introgression through time**

Increased western hunter gatherer (WHG) introgression between the Spanish EN and MN has been previously reported [[6]](https://paperpile.com/c/s9HOea/Zvbll). Here, the test D(Mbuti, WHG; Cardial EN, Portuguese MN/LNCA) was used to investigate the difference in overall western hunter gatherer ancestry between Portuguese Neolithic groups and the Cardial EN. As previously observed a notable increase in WHG ancestry was apparent in the Portuguese MN and LNCA (Z=5.932-6). The above test was then repeated for each of the four individual WHG genomes published. Significant Z scores were achieved for all individuals (Z=4.507-5.506), with the exception of the Hungarian WHG, KO1 (Z=2.787-2.994), suggesting that the increased WHG ancestry present in the Portuguese Neolithic came from a western rather than central European source.

### **4.1.3 Late Neolithic/Chalcolithic Iberia**

The Late Neolithic and Chalcolithic of Iberia dates from approximately 4000-2200 cal BC. A set of whole genomes from Spanish Early Chalcolithic individuals in the Atapuerca Mountains have been previously published in [[7]](https://paperpile.com/c/s9HOea/O70e). These samples date from approximately 3200-2600 cal BC. The affinities of this population and those of the six Portuguese Late Neolithic/Chalcolithic (LNCA) samples were investigated in relation to the preceding Iberian MN groups.

Using the test D(Mbuti, X ; Iberian MN, Iberian LNCA), we find that both Spanish CA and Portuguese LNCA form clades with Portuguese MN to the exclusion of all other ancient groups. However, several ancient populations, including Sintashta, Yamnaya, Unetice and Portuguese LNCA, show near significant introgression into Portuguese MN with respect to Spanish LNCA (Z=2.014-2.751). The Portuguese and Spanish LNCA also form clades with each to the exclusion of other ancient groups - D(Mbuti, X ; Spain LNCA; Portugal LNCA), although again near significant introgression from Yamnaya and Portuguese MN into the Portuguese LNCA is apparent (Z=2.022-2.548). Overall these results may suggest some local continuity between the Portuguese MN to LNCA. However, an immediate archaeological explanation for the observation of near significant Steppe introgression into Portuguese MN and LNCA populations with respect to the Spanish LNCA is unclear.

### **4.1.4 Bronze Age Iberia**

Only one previously published Bronze Age Iberian sample exists [7], sampled from the Atapuerca mountains in Northern Spain. Here, we compare this individual to a group of four Bronze Age contemporaries from south Portugal. We first ask whether we can detect introgression from HG, European BA and Steppe groups into the Spanish and Portuguese BA with respect to preceding Iberian LNCA populations - D(Mbuti, X; Iberian LNCA, Iberian BA). We observe a significant increase in Steppe introgression into the Portuguese BA with respect to the Portuguese LNCA (Z=3.079-3.549), with smaller increases in Corded Ware, BB and Unetice ancestry also observed (Z=1.226-1.623). This introgression is also apparent when the test is repeated with the Spanish CA in the place of Portuguese LNCA. Furthermore, significant WHG and Neolithic introgression into the Spanish CA and Portuguese LNCA to the exclusion of the Portuguese BA is evident. A fainter version of the pattern observed above is apparent also for the Spanish BA sample, the lower coverage of which may have prevented stronger signals from emerging.

We also test whether the Spanish and Portuguese BA form clades to the exclusion of other ancient samples D(Mbuti, X; Spanish BA, Portugal BA). No significant results were obtained. In addition, we ask whether Iberian LN/CA groups form clades with respect to Spanish and Portuguese Bronze Age. No significant introgression was observed. This would suggest no strong regional continuity between the Portuguese LN and BA, with respect to other Iberian populations.

### **4.1.5 Outgroup F3 statistics with modern European populations**

Tests in the form F3(Modern European Population, X; Mbuti) were performed, where X indicates Portuguese BA, LNCA or MN, to identify the modern populations with which each group shares most drift. Results obtained were similar to those presented in [[7]](https://paperpile.com/c/s9HOea/O70e) and are displayed in S5 Fig. Portuguese MN and LNCA individuals scored higher values on average to European populations relative to the Portuguese BA, however very similar patterns of affinity were observed. Both groups showed highest hits with modern Sardinians and Basques. However, in a reversal of what is seen in the Neolithic groups, the Portuguese BA showed highest affinities to populations in the Basque region, rather than Sardinians. A subtle increase in affinity to Northern European populations was also apparent in the BA samples. This may reflect increased continuity between the Basques and Iberian Bronze Age, relative to other populations on the Peninsula.

# References

1. [Mallick S, Li H, Lipson M, Mathieson I, Gymrek M, Racimo F, et al. The Simons Genome Diversity Project: 300 genomes from 142 diverse populations. Nature. 2016; doi:](http://paperpile.com/b/s9HOea/9vraO)[10.1038/nature18964](http://dx.doi.org/10.1038/nature18964)

2. [Mathieson I, Lazaridis I, Rohland N, Mallick S, Patterson N, Roodenberg SA, et al. Genome-wide patterns of selection in 230 ancient Eurasians. Nature. 2015;528: 499–503.](http://paperpile.com/b/s9HOea/F1WW)

3. [Hofmanová Z, Kreutzer S, Hellenthal G, Sell C, Diekmann Y, Díez-Del-Molino D, et al. Early farmers from across Europe directly descended from Neolithic Aegeans. Proc Natl Acad Sci U S A. 2016;113: 6886–6891.](http://paperpile.com/b/s9HOea/KSyt)

4. [Olalde I, Schroeder H, Sandoval-Velasco M, Vinner L, Lobón I, Ramirez O, et al. A Common Genetic Origin for Early Farmers from Mediterranean Cardial and Central European LBK Cultures. Mol Biol Evol. 2015;32: 3132–3142.](http://paperpile.com/b/s9HOea/3e6d)

5. [Cunliffe B. Europe between the Oceans 9000 BC–AD 1000. New Haven-London. researchgate.net; 2008; Available:](http://paperpile.com/b/s9HOea/v0dLs) <https://www.researchgate.net/profile/Jesper_Boldsen/publication/227376643_Barry_Cunliffe/links/540d975f0cf2d8daaacb4e8b.pdf>

6. [Haak W, Lazaridis I, Patterson N, Rohland N, Mallick S, Llamas B, et al. Massive migration from the steppe was a source for Indo-European languages in Europe. Nature. 2015; doi:](http://paperpile.com/b/s9HOea/Zvbll)[10.1038/nature14317](http://dx.doi.org/10.1038/nature14317)

7. [Günther T, Valdiosera C, Malmström H, Ureña I, Rodriguez-Varela R, Sverrisdóttir ÓO, et al. Ancient genomes link early farmers from Atapuerca in Spain to modern-day Basques. Proc Natl Acad Sci U S A. 2015;112: 11917–11922.](http://paperpile.com/b/s9HOea/O70e)

**S5 Table - D-statistics in the form of D(Mbuti, X; Y, Z) to test admixture between ancient populations.**

**S6 Table - Selected D-statistics associated with Portuguese Neolithic and Bronze samples.**

**S5 Fig - Outgroup F3-Statistics in the form F3(Mbuti; X, Modern European Population).**
