# Supplementary material for: The population genomics of archaeological transition in west Iberia: Investigation of ancient substructure using imputation and haplotype-based methods
Source: PLoS Genet. 2017 Jul 27;13(7):e1006852. doi: 10.1371/journal.pgen.1006852 (PMC5531429; doi:10.1371/journal.pgen.1006852)
Supplement: S5 Text — (DOCX) [file pgen.1006852.s005.docx]

# **S5 Text**

# Imputation of missing genotypes in ancient samples

Rui Martiniano, Lara M Cassidy, Ros Ó'Maoldúin, Russell McLaughlin, Nuno M Silva, Licinio Manco, Daniel Fidalgo, Tania Pereira, Maria J Coelho, Miguel Serra, Joachim Burger, Rui Parreira, Elena Moran, Antonio C Valera, Eduardo Porfirio, Rui Boaventura, Ana M Silva, Daniel G Bradley

## **5.1 Genotype imputation in ancient samples**

We used the 1000 Genomes phase 3 dataset (source ftp://ftp.1000genomes.ebi.ac.uk/vol1/ftp/release/20130502/) as provided with BEAGLE, which was filtered for structural variants and for SNPs with <5 copies of the reference allele or <5 copies of the non-reference alleles. For more information regarding filtering criteria, see <http://bochet.gcc.biostat.washington.edu/beagle/1000_Genomes_phase3_v5a/READ_ME_beagle_ref>. Using a similar approach to [[1–3]](https://paperpile.com/c/THdaqt/bSfgS+s4TCb+480lK), we used Beagle 4.0 [[4]](https://paperpile.com/c/THdaqt/LHxLl) to impute missing genotypes in ancient individuals. We selected all published European samples that have been sequenced by whole-genome shotgun sequencing and which coverage is above 0.85X (S7 Table), which we processed in an identical way as for the samples sequenced in the present study. We made the exception to include 2 samples from the present study at ~0.75X coverage. Samples above 15X, Loschbour, LBK [[5]](https://paperpile.com/c/THdaqt/Y66kF), NE1, BR2 [[1]](https://paperpile.com/c/THdaqt/bSfgS) were downsampled to 2X, by selecting random reads with SAMtools v.0.1.19-44428cd [[6]](https://paperpile.com/c/THdaqt/5ASqc). Prior to the variant calling step, we filtered the 1000 Genomes phase 3 dataset ([ftp.1000genomes.ebi.ac.uk/vol1/ftp/release/20130502/](http://ftp.1000genomes.ebi.ac.uk/vol1/ftp/release/20130502/)) by removing all variants that are not SNPs, multiallelic SNPs and X- and Y-chromosomes, after which ~77.8 million (77,818,345) variants remained. Next, we used the Genome Analysis Toolkit (GATK) UnifiedGenotyper [[7]](https://paperpile.com/c/THdaqt/z7oct), to call these variants providing an interval_list file created from the genomic positions in the 1000 Genomes phase 3 (--output_mode EMIT_ALL_SITES, --genotyping_mode GENOTYPE_GIVEN_ALLELES). The resulting VCF file was filtered for potential deamination signals by replacing 0/1 and 1/1 genotypes by ‘./.’, where the reference allele is C or G and the alternate is T or A. We also replaced 0/0 and 0/1 genotypes by ‘./.’ if the reference allele is T or A and the alternate is C or G. We split the filtered VCF file by chromosome using vcftools [[8]](https://paperpile.com/c/THdaqt/hdfEE) and then we used splitvcf.jar to split in 50,000 marker subsets with an overlap of 25,000 markers. Missing genotypes were imputed in each file separately by providing 1000 Genomes phase 3 reference haplotypes (http://bochet.gcc.biostat.washington.edu/beagle/1000_Genomes_phase3_v5a/) and GRCh37 genomic maps (<http://bochet.gcc.biostat.washington.edu/beagle/genetic_maps/>) and parameters as follows: gl=<input>, impute=true, lowmem=false, gprobs=true. This resulted in 30.675.833 markers imputed across 67 individuals.

## 5.2 Assessing imputation accuracy

In order to determine genotype imputation accuracy in in ancient samples, we compared two VCFs, one containing 1000 Genomes phase 3 called genotypes in the 4 high coverage ancient genomes (Loschbour, LBK, NE1, BR2) and the other with imputed genotypes for the same samples previously downsampled to 2X. We first filtered the high coverage VCF by excluding sites with quality below 20 and sites that are above 15x coverage across all individuals (--minQ 20 --min-meanDP 15). Then, we extracted variants of the Hellenthal et al. 2014 in the imputed samples, selecting only those with a GP (genotype probability) above 0.99. Finally, we compared imputed and non-imputed genotypes using SnpSift (Cingolani et al, 2012), obtaining a concordance rate of approximately 99% (S6 Fig).

In order to assess imputation accuracy across the allele frequency spectrum, we took calls filtered by posterior genotype probability threshold of ⋝ 0.99 and grouped into minor allele frequency bins of 0.005. Results are shown in S7 Fig, and suggest, as expected, that genotype imputation is less accurate for lower frequency variants, most likely because these are not present in the phased haplotype dataset used as a reference for imputation. The lower accuracy obtained for variants with global MAF < 0.05 may be improved in the future with the sequencing of several novel high quality ancient genomes of diverse ancestries. However, it is unlikely that rare variants will be imputed accurately and inferences that depend on these will require higher coverage genomes to correctly call genotypes.

## 5.3 Assessing bias in both imputed and pseudo-haploid calls towards 1000G reference panel populations using D-statistics

We set out to explore potential bias in imputed data resulting from the choice of 1000 Genomes reference panel populations used in imputation. This was achieved using D-Statistics of the basic form - D(Chimp, 1KG population; directly called ancient, imputed ancient). We also noted significant biases in pseudo-haploid data and discuss these below.

It is important to note that Chimp may be an imperfect outgroup for tests of reference/imputation bias, dependent on whether majority of ancestral alleles present in chimp are also common human reference alleles. If increased imputation accuracy or alignment quality is present for reference alleles, inflated shared drift may occur between chimp and imputed/pseudo-haploid individuals, relative to diploid calls, which preserve rarer variation.

For this reason, we emphasise relative patterns of bias across difference reference panel populations, rather than overall magnitudes. The majority of tests were carried out using the five high coverage (>15X) ancient samples, all of whom had been down-sampled to 2X for imputation.

## 5.3.1 Pseudo-haploid bias to reference panel is stronger than imputation bias

First, we examined inflated shared drift with reference populations for imputed calls, filtered for a genotype likelihood of 0.99, relative to both pseudo-haploid and true diploid calls. Pseudo-haploid calls were generated at the ~30 M sites used for imputation for the full coverage (>15X) bam file of each sample as described in (Section 5.3.2). Diploid genotypes were called for the same sites as described in Section 5.2 and subsequently filtered for a depth of coverage of 10X and a genotype quality of 30. To control for inaccuracy caused by low MAF we first only examined SNPs that had an overall world MAF of 25% or above in the 1000G dataset (~2.7 M sites). To avoid the confounding effects of damage, transversion SNPs alone were also tested (~890 K sites).

The results of this test, D(Chimp, 1000G Reference population; Ancient Direct Call, Ancient Imputed Call), are displayed in S8 Fig. Positive scores indicate a higher similarity between the imputed data and reference populations relative to directly called genotypes, while for negative scores the reverse is true. It is immediately apparent that the largest bias is that of pseudo-haploid calls towards the reference panel, when compared to imputed calls (S8 Fig; Z = -4.623 to -18.80). In contrast, comparison between diploid and imputed calls, shows definite imputation bias for certain populations, although overall it is smaller and sometimes negative in magnitude (Z = -4.098 to 4.009). A relative increase in affinity in all imputed samples to European reference populations is observed, and also to South Asian populations for the Caucasus HG individual, Kotias. Similar results were obtained for transversion SNPs, with some reduction of imputation bias observed (Section 5.3.4).

## 5.3.2 Imputation bias increases for rarer variants

To further explore the inflated affinity of imputed calls to specific reference panel populations, compared to diploid calls, the test D(Chimp, 1000G Ref; Diploid call, Imputed call) was performed for four different MAF filters (World MAF of 25% and 5%; and European MAF of 25% and 5%), with the results presented in S9 Fig. Again, these tests were carried out also on transversion SNPs alone.

The World MAF filter of 5% resulted in the largest magnitudes of reference bias (Z= 2.20 to 16.60), as well as the largest differences in bias effects, dependent on the ancestry of the reference panel population. The lowest bias effects were seen for a European MAF of 25%, for which the majority of values obtained were in fact negative.

These results suggest that common variants, and more specifically variants that are common in the reference populations most closely related to the tested ancient samples, are less prone to bias, most likely due to increased imputation accuracy.

## 5.3.3 Imputation bias inflates sample affinity to closely related populations from the reference panel.

The overall pattern of imputation bias tends to stay relatively similar across changing MAFs, despite fluctuation in magnitudes. This pattern appears somewhat predictable, in that ancient samples will show the most bias to the populations most closely related to them - Europeans for the majority of ancient samples and also South Asians for Kotias. Consistently lower bias is obtained for African reference populations, a reversal for what is seen for pseudo-haploid calls (Section 5.3.6).

At lower MAF filters further bias for more specific populations is apparent in some samples. For the Neolithic individuals, Stuttgart and NE1, heightened affinity to modern day Iberians (IBS) and Tuscans (TSI) is seen. For the Caucasus HG, Kotias, affinity to Punjabi (PJL) and Gujarati (GIH) is inflated relative to other South Asian populations, while among Europeans, British (GBR) and Tuscans (TSI) show the strongest bias to this individual.

The exception to this rule is seen when a European MAF of 25% is applied, which appears to reduce the overall bias of ancient samples to Europeans, relative to other reference populations, but notably not to South Asians, in the case of Kotias. However, differing affinities for the various European panel populations is still apparent, dependent on ancient sample ancestry (e.g TSI and IBS for Neolithic individuals).

## 5.3.4 Exclusion of genotype information increases imputation bias

When only transversion sites are considered these biases are substantially reduced in magnitude and also show a more even distribution across the reference populations. This is the likely result of decreased imputation accuracy for transitions due to our exclusion of potential damage sites from the dataset prior to imputation. Interestingly, Loschbour does not show such a reduction. The reason for this is unclear, although we note Western European HGs show lower affinity with modern populations, relative to other ancient samples tested, which may be affecting overall imputation accuracies in the sample. Kotias, on the other hand, shows an extreme reduction in bias when only transversions are considered, indicating that prior genotype information for this sample markedly increases imputation accuracy within it.

## 5.3.5 Selecting a panel of variants for use in analyses downstream of imputation

We attempted to reduce imputation bias in our dataset by applying a MAF filter of 5% across the imputed dataset of 67 ancient individuals, prior to merging with the Hellenthal et al. (2014) dataset of modern genotypes. To maintain a robust number of SNPs sufficient for haplotypic analysis, transition SNPs were not discarded

A test of the form D(Chimp, 1000G Ref; Diploid call, Imputed call) was carried out to inform us of the final bias present in the dataset. Results are shown in S10 Fig. Overall scores ranged from Z=0.065 to 5.919 for European ancient samples and Z=7.398 to 9.615 for the Caucasus HG individual, Kotias. However, for European ancient samples differences in magnitudes between non-African reference panel populations were relatively minor, with Kotias showing higher variability.

## 5.3.6 Pseudo-haploid bias

A common practice is to construct individual genotypes for analysis by sampling only one allele at each polymorphic site. These pseudo-haploid calls introduce bias which we explored with the test D(Chimp, 1000G Ref; Diploid Call, Pseudo-haploid Call) (S11 Fig) with two different MAF filters imposed (World MAF of 25% and 5%). Only transversion sites were considered to avoid the confounding effects of damage. Significant biases to the reference panel were observed (Z = -0.768 to 3.65 and 1.44 to 4.099 for World MAFs 25% and 5% respectively). Importantly, bias was distributed unevenly with regard to reference population tested. The majority of samples show strongest bias to African populations, followed by Europeans. Decreasing MAF from 25% to 5% increased bias for the majority of tests, Loschbour being a notable exception. However, decreasing MAF also reduced the relative bias towards European reference populations, although bias to African populations remained the highest.

Given that the randomized nature of pseudo-haploidization should not in and of itself create bias to certain populations over others, the problem may stem from alignment bias to the reference genome used, an issue that has been previously noted [9]. Reads possessing alleles alternate to the reference sequence may receive lower mapping qualities, and subsequently be filtered out, which in turn would bias haploidization towards reference alleles. This bias would be especially pronounced in ancient data, where high levels of post mortem damage already decrease alignment quality [[10]](https://paperpile.com/c/THdaqt/zVqx). A preponderance of reference alleles, and loss of rarer variation, relative to diploid genotypes, would inflate affinity of pseudo-haploid samples to modern populations, particularly to those whose ancestry composes the human reference genome. Importantly, the application of MAF filters, may affect biases in unpredictable ways, depending on the ancestries of the samples and populations involved, as observed in S11 Fig.

# References

1. Gamba C, et al. (2014) Genome flux and stasis in a five millennium transect of European prehistory. Nat Commun 5:5257.

2. Jones ER, et al. (2015) Upper Palaeolithic genomes reveal deep roots of modern Eurasians. Nat Commun 6:8912.

3. Martiniano R, et al. (2016) Genomic signals of migration and continuity in Britain before the Anglo-Saxons. Nat Commun 7:10326.

4. Browning SR, Browning BL (2007) Rapid and accurate haplotype phasing and missing-data inference for whole-genome association studies by use of localized haplotype clustering. Am J Hum Genet 81:1084–1097.

5. Lazaridis I, et al. (2014) Ancient human genomes suggest three ancestral populations for present-day Europeans. Nature 513(7518):409–413.

6. Li H, et al. (2009) The Sequence Alignment / Map (SAM) Format and SAMtools 1000 Genome Project Data Processing Subgroup. Bioinformatics 25:2078–2079.

7. McKenna A, et al. (2010) The Genome Analysis Toolkit: a MapReduce framework for analyzing next-generation DNA sequencing data. Genome Res 20(9):1297–1303.

8. Danecek P, et al. (2011) The variant call format and VCFtools. Bioinformatics 27(15):2156–2158.

9. Günther T. (2016). Do we always need to remap published aDNA data? Retrieved from
https://tgnthr.wordpress.com/2016/11/23/do-we-always-need-to-remap-published-adna-data/

10. Schubert M, et al. (2012) Improving ancient DNA read mapping against modern reference genomes. BMC Genomics 13:178.

**S7 Table - List of ancient samples selected for genotype imputation.**

**S6 Fig - Estimation of Imputation accuracy on chromosome 21.**

Comparison of variant calls obtained for BR2, NE1, Loschbour and Stuttgart at full coverage with genotypes from the same 4 individuals downsampled to 2 and subsequently imputed. Accuracy in (A) all 3 types of genotypes; (B) homozygous reference; (C) heterozygous and (D) homozygous alternate.

**S7 Fig - Proportion of correctly imputed genotypes grouped by minor allele frequency bins of 0.005.**

In this analysis, imputed genotypes were filtered by post imputation genotype probability ⋝ 0.99.

**S8 Fig - Affinity of imputed calls to reference panel populations, relative to pseudo-haploid and diploid calls, for five high coverage ancient samples.**

Results are shown for both all sites and just transversions in two separate panels. A world minor allele frequency of 25% has been applied. 1000 Genomes population and superpopulation names are noted along the X axis.

**S9 Fig - Affinity of imputed calls from five high coverage ancient samples to reference panel populations, relative to diploid calls, for a series of MAF filters.**

Results are shown for both all sites and just transversions in on left hand and right hand panels respectively. Top panels display world MAF filters of 25% and 5%. Bottom panels display European MAF filters of 25% and 5%. 1000 Genomes population and superpopulation names are noted along the X axis.

**S10 Fig - Affinity of imputed calls from five high coverage ancient samples to reference panel populations, relative to diploid calls, for the final set of SNPs used in downstream analyses. 1000 Genomes population and superpopulation names are noted along the X axis.**
**S11 Fig - Affinity of pseudo-haploid calls to reference panel populations, relative to diploid calls, for five high coverage ancient samples.**

Results are shown for world MAF filters of 25% and 5%. Only transversion SNPs are considered. 1000 Genomes population and superpopulation names are noted along the X axis.
