# Supplementary material for: The population genomics of archaeological transition in west Iberia: Investigation of ancient substructure using imputation and haplotype-based methods
Source: PLoS Genet. 2017 Jul 27;13(7):e1006852. doi: 10.1371/journal.pgen.1006852 (PMC5531429; doi:10.1371/journal.pgen.1006852)
Supplement: S6 Text — (DOCX) [file pgen.1006852.s006.docx]

# **S6 Text**

# CHROMOPAINTER and fineSTRUCTURE analyses

Rui Martiniano, Lara M Cassidy, Ros Ó'Maoldúin, Russell McLaughlin, Nuno M Silva, Licinio Manco, Daniel Fidalgo, Tania Pereira, Maria J Coelho, Miguel Serra, Joachim Burger, Rui Parreira, Elena Moran, Antonio C Valera, Eduardo Porfirio, Rui Boaventura, Ana M Silva, Daniel G Bradley

## **6.1 CHROMOPAINTER analysis I - aDNA samples only**

### **6.1.1 Investigation of haplotype sharing in ancient Eurasians**

In CHROMOPAINTER analysis I, we included 67 imputed aDNA samples, of which 10 belong to the samples from the present study. We first removed from the imputed dataset variants with posterior genotype probabilities inferior to 0.99 using the script “filter_GP_vcf.py”. Next, we remove genotypes with MAF < 5% and SNPs with missing genotypes using vcftools [[1]](https://paperpile.com/c/qIvJZ3/pcBQg), which resulted in a significant decrease in SNP numbers (1.489.769 out of 30.675.833). We split the data by chromosome and to avoid re-phasing these genotypes we converted from VCF to IMPUTE2 format with bcftools version 1.2 (https://samtools.github.io/bcftools/bcftools.html). Hap files were converted to CHROMOPAINTER format "impute2chromopainter.pl” and created a recombination map with “makeuniformrecfile.pl”, both available at <http://www.paintmychromosomes.com/>.

The first step consists of mutation rate and recombination rate estimation, followed by estimating the normalization parameter “c” [[2]](https://paperpile.com/c/qIvJZ3/0G6gp). We have ran these computational tasks in HPC mode, by using fineSTRUCTURE to write the necessary commands to run the analysis and combine the output before starting the subsequent step. “Chromocombine” was then used to create a genome wide CHROMOPAINTER output for all individuals. For the fineSTRUCTURE analysis, the following settings used were: 3,000,000 burn in iterations, 1,000,000 sample iterations for the MCMC and 100,000,000 tree iterations. We share the coancestry matrix obtained for this analysis in S8 Table. fineSTRUCTURE was also used to estimate the Principal Component Analysis presented on the main text.

fineSTRUCTURE allowed obtaining detailed clustering within ancient samples, with 19 genetically defined populations. Regarding hunter-gatherer samples, we identified two separate clusters for WHG (Bichon and Loschbour, KO1 and LaBrana), one composed only of SHG (Motala and Ajvide) and one composed of CHG (KK1 and SATP). Four separate clusters were identified between the EEF samples included in our analysis - four separate clusters within EEF (Turkish, Greek and Hungarian EN; Spanish EN and Hungarian EN and Copper Age; Atlantic MN and LN clusters; and the Tyrolean Iceman Otzi). Lastly, from the Copper Age/Bronze Age period onwards, we obtain 11 additional clusters. We briefly describe the populations identified below.

####

#### 6.1.1.1 Hunter-gatherer samples

##### 6.1.1.1.1 Western Hunter-gatherers I (Western_HG1)

Bichon is an Upper Paleolithic from after the Last Glacial Maximum (LGM), while other WHG (Loschbour, LaBrana, KO1) all belong to a similar time slice of the Mesolithic ca. 6000 BC. Despite this ~6.000 years difference, Bichon’s similarity with younger WHG has been described [[3]](https://paperpile.com/c/qIvJZ3/0zKib). Interestingly, fineSTRUCTURE does not cluster the groups in a chronological manner. Instead, we see Bichon and Loschbour forming a separate cluster hinting at some level of geographical continuity in the Rhine basin (S12 Fig).

##### 6.1.1.1.2 Western Hunter-gatherers II (Western_HG2)

Despite belonging to geographically distinct hunter-gatherer populations in Iberia and Hungary, LaBrana and KO1 were included in the same population. A recently published study [[4]](https://paperpile.com/c/qIvJZ3/jWJGL) used f3 statistics to group individuals, and Hungarian KO1 and LaBrana were positioned in the same clade, however this has been interpreted as a possible artifact deriving from both samples being non-UDG-treated. In the present study, we excluded C->T and G->A changes prior imputation and therefore differences in UDG treatment between samples should not significantly affect our analysis. The positioning of KO1 and LaBrana in the same population may therefore represent real shared recent ancestry (S13 Fig).

##### 6.1.1.1.3 Scandinavian hunter-gatherers (Scandinavian_HG)

As posited in several studies [[5,6]](https://paperpile.com/c/qIvJZ3/epvLT+tdKbB), we confirm that Scandinavian hunter-gatherers (Ajvide58 and Motala12) form a cluster distinct from all WHG genomes (S14 Fig). The Motala sample, dated from ~ 5700 BC, predates the arrival of agriculture in Scandinavia, while Ajvide58, a belongs to the Pitted-Ware Culture. This was a Neolithic foraging culture that coexisted alongside agriculturalists for more than a millennium [[7]](https://paperpile.com/c/qIvJZ3/8K0yp) Their genetic similarity is further supported by the fact that both samples present the derived allele at Y-chromosome marker I2a1.

##### 6.1.1.1.4 Caucasus Hunter-gatherers (Caucasus_HG)

Two Caucasus hunter-gatherers (CHG), were included in the analysis: Satsurblia and Kotias, dated to the Upper Paleolithic and Mesolithic, respectively, have been shown to be a distinct population from European hunter-gatherers and Early Farmers samples [[3]](https://paperpile.com/c/qIvJZ3/0zKib). In the present analysis, both CHG samples form a cluster to the exclusion of all other samples, providing further support to the notion of continuity in this region, emphasizing their placement as a unique ancestral hunter-gatherer group (S15 Fig). This is also supported by Y-chromosome affinities with both belonging to haplogroup J. Genetic similarity between the two samples has also been demonstrated by their inclusion in the same cluster in a f3-based analysis [[4]](https://paperpile.com/c/qIvJZ3/jWJGL).

#### 6.1.1.2 Neolithic samples

##### 6.1.1.2.1 Turkish and Greek Neolithic and Central European LBK (AegeanEN_HungarianLBK)

In the present analysis, Turkish Early Neolithic (Bar31 and Bar8), Greek Early (Rev5) and Late Neolithic (Klei10, Pal7) , as well as Hungarian (NE6) and German (Stuttgart) Neolithic samples associated with the Linearbandkeramik culture were included in the same population (S16 Fig). All these samples contain mtDNA haplogroups usually present in Neolithic samples, K, X, J and T2 (Brandt et al., 2014). A previous CHROMOPAINTER analysis (using unlinked SNPs, not haplotypes) of a similar set of Neolithic samples (Hofmanova et al., 2016) suggested that the Aegean genomes contributed the highest amounts of genetic ancestry to Hungarian and German Early Neolithic Genomes.

##### 6.1.1.2.2 Hungarian Neolithic and Cardial Spanish (HungarianMLN_SpainCardialEN)

Greater genetic similarity is seen between Early Neolithic (NE1 and NE7) and Copper Age (CO1) samples from Hungary and one EN Cardial sample from Spain (CB13) (S17 Fig). This link between an Early Iberian and a subset of Hungarian samples probably reflects a common origin for first farmers. As suggested by [[8]](https://paperpile.com/c/qIvJZ3/haBmu), CO1 belonging to the same population as other Neolithic is a signal of genetic continuity. In addition, a link between Hungary and Spain had already been established, with Spanish CB13 hunter gatherer ancestry showing increased affinity to KO1 than LaBrana [[9]](https://paperpile.com/c/qIvJZ3/8oCuH).

##### 6.1.1.2.3 Atlantic Neolithic (Atlantic_Neolithic)

Our analysis reveals the inclusion of Portuguese Middle Neolithic and Late Neolithic we sequenced in the present work in the same cluster as Spanish Late Neolithic/Chalcolithic (Thorsten et al., 2015), Irish Middle Neolithic (Cassidy et al., 2015) and a Scandinavian farmer (Skoglund et al., 2014) (S18 Fig). This might suggest a link between Neolithic samples in Atlantic Europe which coincides with the presence of Megalithic cultures [[10]](https://paperpile.com/c/qIvJZ3/Y99ha). Increased affinity between MN/Chalcolithic samples from these regions had previously been suggested [[11]](https://paperpile.com/c/qIvJZ3/j3m0l), and now their inclusion in same genetic population provides further evidence for recent shared ancestry between Atlantic Neolithic samples.

####

#### 6.1.1.3 Late Neolithic/Copper Age and subsequent time periods

##### 6.1.1.3.1 Yamnaya and Afanasievo (Yamnaya_Afanasievo)

Our fineSTRUCTURE analysis placed the 2 Yamnaya samples (Copper Age pastoralists) and a single Afanasievo individual in the same cluster, proving the effectiveness of this method for detecting links between distant, but related archaeological cultures (S19 Fig). The genetic similarity between these two groups had been shown by Allentoft et al., (2015) and interpreted as eastward migrations from Yamnaya to the steppe.

##### 6.1.1.3.2 Sintashta, Andronovo and Hungarian Iron Age (Sintashta_Andronovo)

Our analysis only included a single Late Bronze Age Sintashta individual, which clusters with two later Andronovo samples and an Iron Age Hungarian (S20 Fig). Archaeological elements support the proposition that the Andronovo culture derived from the Sintashta and this idea was reinforced by the genetic similarity between samples of both cultures [[12]](https://paperpile.com/c/qIvJZ3/fgJeD). The clustering obtained in this analysis provides an additional source of evidence in favour of this genetic proximity, but also suggests that expansions to the West may have contributed to the Hungarian Iron Age.

##### 6.1.1.3.3 North Western and Central European (CopperAge_to_AngloSaxon)

This population is composed of multiple (n=13) Central and Northwestern European samples from the Late Neolithic to the Anglo-Saxon period in Britain (S21 Fig). These samples are: a Copper Age Bell Beaker sample from Czech Republic (RISE569), Early Bronze Age Czech (RISE569), Polish (RISE150, RISE577) and Irish (Rathlin1 and RSK1) individuals, Scandinavian Late Neolithic (RISE98) and Iron Age (RISE174) and finally, Roman British (6DRIF-3, 6DRIF-18, 6DRIF-21, 6DRIF-22) and Anglo-Saxon from York (NO3423).

Our analysis reinforces the idea of a genetic link between Central European EBA samples and the Bronze Age in NW Europe (specifically Ireland) as suggested previously [[11]](https://paperpile.com/c/qIvJZ3/j3m0l). There is also some support for a connection between the British Isles and Central Europe at level of Y-chromosome lineages, since ⅞ males from this cluster present the derived allele at R1b-M269, except for the Anglo-Saxon individual which has been identified as belonging to hg I1 [[13]](https://paperpile.com/c/qIvJZ3/V2Ck2).

##### 6.1.1.3.4 Hungarian Bronze Age (Hungary_BA)

Our fineSTRUCTURE analysis allowed for distinguishing 2 Hungarian Bronze Age individuals from the remaining Central and Western European clusters (S22 Fig). Although in PCA they appear to be distant from each other when compared to other individuals, fineSTRUCTURE was able to detect common recent ancestry.

Many of the different populations are readily observed in PCAs calculated with haploid genotypes. However, this sometimes results in subjective observations regarding population history. Furthermore, PCAs typically display the first and the second component, which describe the majority of the variability in a dataset, but this is usually a modest fraction of the total variation present in a dataset. The application of clustering methods to ancient data provides additional information regarding common recent ancestry and allows for the identification of populations composed of genetically indistinguishable individuals.

##### 6.1.1.3.5 Portuguese Bronze Age (Portugal_BA)

In the present analysis, fineSTRUCTURE has identified the 3 Portuguese Bronze Age individuals as a genetically distinct population (S23 Fig). When compared to Central or Northern European populations such as Ireland [[11]](https://paperpile.com/c/qIvJZ3/j3m0l), the degree of discontinuity between the Neolithic and Bronze Age in Portugal is not pronounced. However, despite the small sample size we have evidence suggesting complete discontinuity at the level of Y-chromosome lineages with all 3 male Bronze Age samples presenting derived alleles at marker M269.

Although in ADMIXTURE analysis we were not able to observe the presence of the CHG-related cluster in the ancestry proportions of the Portuguese Bronze Age samples, with D(Mbuti, X; Portuguese MN/LNCA, Portuguese BA) we find support for CHG/Yamnaya related introgression and also an increase in EHG ancestry.

In PCAs these samples are positioned between Central/North Western European Bronze Age and Iberian MN/LNCA Taken together, our results suggest moderate, yet significant admixture of an external source from populations with steppe-related ancestry, which have resulted in the differentiation of the Portuguese Bronze Age samples.

##### 6.1.1.3.6 Russian Late Bronze Age (Russia_LBA)

From the Late Bronze Age to Iron Age cultures, although many of these samples belong to broadly the same region, they are placed in distinct populations, and greater diversity has been described at the level of archaeology and genetics [[12]](https://paperpile.com/c/qIvJZ3/fgJeD) (S24 Fig). In ADMIXTURE analysis, we also observe an increase in the Asian-like cluster (purple) ancestry in Mezhovskaya and Karasuk, and also highlights the diversity in within this region during the LBA. In PCAs, East Asian-related admixture is visible with time from the LBA to the Iron Age.

##### 6.1.1.3.7 Russian IA and Karasuk (Russia_LBA_IA)

2 Russian IA (RISE601 and RISE602) and 2 LBA Karasuk (RISE493 and RISE495) samples were placed in the same population (S25 Fig). The PCA analysis demonstrates this close genetic affinity between these 4 samples.

##### 6.1.1.3.8 Clusters formed by a single sample

The Tyrolean Iceman (Keller et al., 2012) is placed in a separate cluster on its own, instead of with the EEF group of samples (Lazaridis et al., 2014). This may be because of fineSTRUCTURE’s increased capacity for detecting more subtle differences than other methods that rely on unlinked SNPs. Two Karasuk samples (RISE502 and RISE497) were placed in individual clusters and in PCAs they do not cluster with each other and show variable amounts of Eastern ancestry. One Russian Iron Age (RISE504) sample from Kytmanovo was also left excluded from all cluster. Lastly, 3DRIF-26, which presents greater affinity with Middle Eastern populations, forms a separate population from all other samples, as expected, since the dataset does not contain other samples with similar ancestry.

### **6.1.2 Investigation of hunter-gatherer affinity in aDNA samples**

In order to investigate regional hunter-gatherer affinity in our dataset, we compared haplotype donation from HG samples to samples from Neolithic, Bronze and subsequent time-periods. First, we selected from the coancestry matrix obtained above vectors of haplotype donation from LaBrana, Loschbour, Bichon and Koros1. Next, we calculated the difference between the vectors of each one of the HG X, where X={LaBrana, Loschbour, Bichon} and located more towards the West, and the vector of haplotype donation from Hungarian HG, which is geographically more eastern. Negative values indicate an excess of haplotype reception from a source most similar to the Hungarian HG KO1 than to HG X and according to our results, this was observed for every Neolithic sample outside Iberia, except for an EN Spanish Cardial sample. In the Iberian Middle and Late Neolithic/Chalcolithic, the patterns of haplotype donation are inverted and present instead an increase in sample affinity with HG X when compared to the Hungarian HG. We interpret this shift in HG ancestry in Iberian samples as the result of an increase in local (more western) HG admixture: the EN Cardial CB13 received more haplotypes from KO1 than HG X, highlighting its similarity with other Central European Neolithic samples, and MN/LNCA Iberian Neolithic individuals received more haplotypes from HG X than KO1, which results from a admixture with local HGs. We performed non-parametric Mann-Whitney tests with R to compare differences in excess haplotype donation between samples. One limitation of our analysis is that we did not include Eastern HGs, an important source of Eurasian ancestry, into the coancestry estimation because of lack of samples above the minimum genomic coverage threshold imposed for imputation.

## **6.2 CHROMOPAINTER analysis II - aDNA samples and present-day Eurasians + Yoruba**

Starting with the aDNA imputed dataset described above, we extracted 472.565 SNPs genotyped in present-day populations [[14]](https://paperpile.com/c/qIvJZ3/mxZR3) which we mapped to b37 (GRCh37) of the human genome. From the modern dataset, we extracted European, West-Asian, Middle Eastern, Yoruba and Han Chinese individuals and merged these with the ancient samples, excluding problematic SNPs and filtering by genotyping rate 0.01 and MAF < 5%, which resulted in a dataset of 287.334 polymorphisms genotyped across 805 individuals. Similarly to above, we split the data by chromosome, but the generation of a new dataset required a phasing step which we performed with shapeit v2.r778 [[15]](https://paperpile.com/c/qIvJZ3/ZAlmX). The subsequent steps were the same as described above.

### **6.2.1 Total Variation Distance**

Next, we estimated the Total Variation Distance (TVD) in a similar way to what was described previously [[16–19]](https://paperpile.com/c/qIvJZ3/zVXBr+o0rx6+KFIX1+3mLoy). Briefly, TVD here was calculated by first obtaining vectors of median haplotype donation (present in the coancestry matrix) from each ancient sample to present-day populations. Then we calculated the difference between vectors donated by pairs of one Bronze Age and one Neolithic sample from the same region when possible (Portuguese: MonteGato104 and LugarCanto42; Irish: Rathlin1 and Ballynahatty, Hungary; BR2 and NE1, Germany: RISE548 and LBK, Scandinavia: RISE98, Gok2, Eastern Europe: RISE548 and LBK. Two observations emerge from this analysis: 1) Bronze Age samples tend to donate more haplotypes to Central and Northern European populations and Neolithic samples donate more haplotypes to Southern groups, in particular the Sardinians; 2) The difference between vectors of haplotype donation between Bronze Age and Neolithic samples is more pronounced in the analysed Central and Northern regions, while in the South, especially in Iberia, this difference is very reduced, supporting the idea that the population turnover that occurred in Europe with migrations from the Steppe was less extensive in Iberia than in Central and Northern Europe.

### **6.2.2 Clustering of ancient DNA samples with present-day individuals**

We used the chromosome painting matrix obtained above to run fineSTRUCTURE (3,000,000 burn in iterations, 1,000,000 sample iterations for the MCMC and 100,000,000 tree comparisons). One of the most evident results of this analysis is that while the more distantly related hunter-gatherer and Neolithic period samples tend to form clusters with other ancients their contemporary. However, from the Bronze Age onwards, aDNA samples tend to be included in the same populations as modern individuals. This analysis contains ~287 thousand SNPs, approximately 1 million less than in Analysis I, but it also offers ~700 more individuals. This resulted in loss of resolution in some cases detect population structure, affecting mostly the Neolithic samples, and in other cases there was some benefit, in particular from the Bronze Age onwards. A brief description of the clustering obtained with ancients and moderns is presented below. We share the coancestry matrix obtained in S9 Table.

#### 6.2.2.1 Hunter-gatherers

The 2 CHG and Georgians belong to sister clades, sharing a common ancestor with the Adigey, a Northern Caucasus group. This provides further evidence of genetic continuity in the region. We obtained identical clustering with all other HG samples.

#### 6.2.2.2 Neolithic

The Atlantic cluster is a sister group to modern Sardinians. It now contains CB13, the Cardial Neolithic sample from Spain. Gok2 was now placed with Portuguese Bronze Age samples, perhaps because of having some level of EHG introgression. Neolithic samples from Turkey, Greece and Hungary, including LBK, are now in the same population, instead of separated in 2 distinct clusters.

#### 6.2.2.3 Bronze Age and onwards

The Portuguese Bronze Age samples are phylogenetically closer to Sardinians and Neolithic samples. We obtained increased resolution in the Central/Northwestern European cluster of samples identified in Analysis I. Two Unetice samples (EBA) from Poland (RISE150, RISE577), the Irish Bronze Age (Rathlin1, RSK1) and the Scandinavian Late Neolithic/Bronze Age (RISE98) samples form their own cluster. Roman British samples were included in the same population as modern individuals from the British Isles (Irish, Scottish, Welsh, English). The Anglo-Saxon (NO3423) and Nordic Iron Age (RISE174) are placed in the same population as modern Norwegian, indicating some level of continuity in this region at least from the Iron Age Period onwards.

Hungarian EBA sample from Vatya was now in the same population as present-day Polish and Belorussian. The Hungarian Iron Age sample is now placed in the same population as a subset of Bulgarian, Georgian and Armenian. This sample is described as pre-Scythian (Gamba et al., 2014) and belonging to the Mezocsat culture, which has been associated with the Cimmerians, which in turn have probably originated in the Pontic Steppe and expanded via Caucasus/Balkans towards Hungary. The Hungarian BR2 and the Bell Beaker from Czech Republic (RISE569) is included in the same population as present-day Hungarians.

As in Analysis I, one Russian LBA (RISE497) and one IA (RISE504) individuals, form distinct populations independent of any other ancient or modern sample. They form a sister group with the Han Chinese, which derives from East Asian introgression already observed in other analyses, such as ADMIXTURE in the present work.

Sample 3DRIF-26, previously described as being more similar to Middle Eastern populations (Martiniano et al., 2016), was positioned in the same branch as Syrian and Iranian samples and this forms a sister clade to a subgroup of Druze.

Sintashta and Andronovo cultures remain in the same population, as well as Yamnaya and Afanasievo samples. The population with 3 LBA Karasuk samples remained the same as in Analysis I. Karasuk culture sample RISE502 was now placed together with a previously identified cluster composed of 2 other samples from the same site and also 2 Iron Age from Russia (Verh-Ulmon and Sary-Bel). This group of samples is phylogenetically closer to the Chuvash population.

## **6.3 CHROMOPAINTER analysis III - comparison of linked and unlinked analyses**

In order to assess the benefit of incorporating LD information into population structure, we took the dataset generated in section 6.1 and pruned the data for linked SNPs using PLINK (--indep-pairwise 50 10 0.1), which removes any SNP correlated (r^2^ > 0.1) with neighbouring SNPs within a 50 SNP sliding window, starting at every 10 bp, resulting in 49.053 SNPs. Steps in Section 6.1 were repeated, except for the generation of a recombination map, which is not required for running an unlinked CHROMOPAINTER analysis. Overall, we have identified 19 clusters when using haplotype information in the CHROMOPAINTER analysis, which is almost a twofold increase in resolution when compared to the 10 populations detected using only unlinked SNPs (S26 Fig). In the unlinked analysis (which ignores linkage information) we observed the following differences when compared to the linked clustering:

Western hunter-gatherers LaBrana, Bichon, KO1 and Loschbour are included in the same population in the unlinked analysis, but when running with linkage information, CHROMOPAINTER divided these samples into 2 populations. The Scandinavian hunter-gatherer cluster continues to be separated in this analysis.

The unlinked analysis does not separate Atlantic Neolithic and Portuguese Bronze Age samples, which are clearly separated when using LD information. Early Neolithic Aegean, Hungarian and Spanish (CB13) and Copper Age Otzi are grouped in a single population, while the linked CHROMOPAINTER analysis detects 3 distinct clusters.

A very large population formed of 16 mostly Central and Northwestern samples ranging from Copper Age to the Anglo-Saxon period. Hungarian Bronze Age and Russian Sintashta were now placed in the same population as the previously identified NW European cluster. In the linked run, the Yamnaya samples were placed in the same population as Afanasievo, but now 5 other Russian samples belonging to Middle (Andronovo) and Late Bronze Age (Karasuk) are also included in this group. Russian Iron Age samples belonging to individual clusters are now grouped in the same population.

Note that we obtain identical results when running an unlinked analysis on the 1.3 M SNPs dataset (Analysis I). This indicates that incorporating LD information is crucial for more powerful detection of subtle population structure.

## **6.4 CHROMOPAINTER analysis IV - Analysis with unfiltered genotype probabilities**

In the previous analyses, we had filtered the imputed dataset of ancient samples with genotype probability of at least 0.99, which results in the loss of many genotypes (mostly heterozygotes) but also in excellent accuracy (see above). These heterozygotes are probably rarer, and might be shared by subsets of one or two ancient samples, and therefore reveal important connections through sharing of recent common ancestry. In order to explore this possibility, we performed a fineSTRUCTURE analysis with the same steps as above in section 6.1 (--maf 0.05 and --geno 0) but we did not filter genotypes according to posterior genotype probability, resulting in ~6.5 M SNPs genotyped across all samples.

In the unfiltered analysis fineSTRUCTURE detected 26 populations, 7 more than when filtering by posterior genotype probabilities ⋝ 0.99. The main differences at the level of population clustering are:

Bichon and Loschbour remain in the same cluster, but not KO1 and Labrana, or Motala and Ajvide, which are now separated. The Cardial EN CB13 is no longer part of a cluster with other Neolithic samples and now forms a cluster on its own. As in Analysis II, the Portuguese Bronze Age cluster now contains the Scandinavian farmer Gok2 perhaps caused by EHG-related ancestry. The Irish Bronze Age samples, Rathlin1 and Rathlin2 form a separate cluster and a Polish Unetice sample was positioned in the same cluster as Nordic IA. One other cluster identified contained a Czech Unetice sample and a Bell Beaker also from Czech Republic, Roman samples and a Anglo-Saxon. Andronovo and Sintashta are now separated, but are still in sister clades.

Despite very encouraging results, such as for example, the formation of an Irish Bronze Age cluster, the increase of informativeness derived from a much larger number of SNPs comes at the cost of introduction of errors and therefore uncertainty: in the case of heterozygous genotypes approximately 6% of calls are incorrect when unfiltered for genotype probability.

## **6.5 CHROMOPAINTER analysis V - Detection of biases in CHROMOPAINTER analyses derived from genotype imputation in ancient samples**

### **6.5.1 Dataset preparation**

The main concern regarding imputation of missing genotypes in ancient samples is the introduction of biases when using present-day populations as reference haplotypes. In principle, this issue should result in increased similarity between ancient and modern samples present in the reference haplotype dataset used for phasing and imputation. To investigate whether such biases exist, as well as to what extent they influence haplotype-based methods such as CHROMOPAINTER, we used 3 available high coverage genomes from different time-periods (Loschbour (HG), LBK (Neolithic farmer) and BR2 (Bronze Age)), from which we have generated 2 sets of data for each one of the ancient samples: one with imputed genotypes and the other with variants called without subsequent imputation.

As described in S5 Text, we downsampled reads approximately 2X genomic coverage and subsequently imputed with 1000 Genomes phase 3 reference dataset. Next, for each ancient sample separately, we merged imputed genotypes with a present-day populations dataset published by ref. [[14]](https://paperpile.com/c/qIvJZ3/mxZR3) composed of European, Middle Eastern and West Asian individuals, removing variants that were not present in all individuals and with minor allele frequency < 5%.

We then called genotypes in the high coverage genomes as in [[11]](https://paperpile.com/c/qIvJZ3/j3m0l). In summary, diploid genotypes calls for each position in the Hellenthal et al. (2014) dataset were generated using the UnifiedGenotyper tool in GATK v2.4-7 (with parameter -mbq 30). Genotype calls were filtered for a depth of coverage of 10X or above and a genotype quality of 30 or above. Each sample was then individually merged with a set of modern Eurasian genotypes as above, keeping only SNPs confidently called across all individuals and removing variants with MAF < 5%.

Next we extracted individuals and variants shared by both datasets for each ancient sample, so that the only difference is in the ancient samples’ genotypes, which have been imputed in one dataset in the other they have not.

### **6.5.2 Comparison of CHROMOPAINTER copying vectors between imputed and non-imputed samples**

We phased these datasets and ran CHROMOPAINTER/fineSTRUCTURE as described above, resulting in 6 distinct analyses, 2 per sample. For the output of both runs, we calculated median donated haplotypes by each one of the ancient samples to modern populations, which we compared using the “visreg” package [[20]](https://paperpile.com/c/qIvJZ3/hKISs) of the R programming language [[21]](https://paperpile.com/c/qIvJZ3/0Rr6y). We observe very highly correlated values (r^2^> 0.94, S27 Fig, a1-3), with the best correlated donation vectors belonging to imputed and non imputed Loschbour (r^2^=0.984).

In order to statistically test if aDNA samples become more similar to the modern populations present in the reference haplotypes used in imputation, we used normal Quantile-Quantile plots to detect outliers from residuals (labelled populations) (S27 Fig, b1-3). Imputation appears to introduce the biggest bias in Loschbour (b2), with a noticeable increase (7.61%) in the median value of donated haplotypes to the English population, which is represented in the 1000 Genomes dataset as GBR (British in England and Scotland). Imputed BR2 (b1) and LBK (b3) also show a moderate increase in donation to the English population (4.35% and 1.17%, respectively), but these are not statistical outliers. Loschbour (b2), also shows an increase in Belarusian ancestry (5.23%), a population which is not represented in the 1000 Genomes reference data (although there might be some affinity with CEU), and a decrease in Russian ancestry (7.03) when compared to the called variant analysis.

Imputed BR2 appears shows an increase in GermanyAustria (6.54%) and a decrease in Armenian (5.35%) and Welsh (4.74%). Regarding LBK, the imputed analysis appears to be enriched for Irish ancestry (7.29%), and it has considerably less Finnish (14.07%) and Belarusian (10.12%) (S27 Fig, c1-3).

Differences in imputation must be certainly related to the ancestry of the ancient sample, but according to our analysis, we do not have enough indication that these bias will qualitatively affect our findings. There is no strong evidence for systematic changes being caused by genotype imputation. Although there are moderate differences (at most 14%) between imputed and non imputed donated haplotypes, we don’t see ancient samples consistently donating more haplotypes to populations present in the 1KG. Note that the sum of the absolute difference between the vectors being compared (i.e. imputed Vs. not imputed) is very small, consisting only of 3 to 4% of the total number of haplotypes.

### **6.5.3 Comparison of fineSTRUCTURE clustering between imputed and non-imputed samples**

In order to test if small differences in copying vectors caused by imputation (above analysis) would affect the clustering results of ancient DNA samples, we ran fineSTRUCTURE with steps as in analysis I (3,000,000 burn in iterations, 1,000,000 sample iterations for the MCMC and 100,000,000 tree iterations). The clustering obtained in runs with imputed and non-imputed samples are very similar for all samples (S28 Fig), providing further support to a very modest effect from imputation on fineSTRUCTURE clustering and that in cases where we are not able to call diploid genotypes, our analysis with imputed data would probably very similar to the one made with diploid data from those samples.

Reassuringly, BR2 is being included in the same clade as present-day Hungarians, and in the proximity of a majority of Romanian and Bulgarian clusters. Loschbour is positioned in an ancestral population of Northwestern European samples, namely Norwegian, Orcadian, English, Scottish, Welsh and Irish. LBK is also placed as an ancestral population to a cluster composed of mostly Tuscan and North Italians in both analysis, but in the non-imputed, a single South Italian individual is also positioned in the proximity of LBK which we do not see in the imputed data, which is unexpected, since we are using modern haplotypes to impute ancient samples we expected the ancients to become more similar to present-day populations. Nevertheless, both LBK and Loschbour are both included on a population on their own in both imputed and non-imputed data. As observed in other analyses, samples from the Bronze Age or more recent are included in the same population as modern individuals, all other older samples normally form a cluster on their own.

Overall, our results indicate that there are small differences in the values of haplotype sharing between imputed and non imputed data. It is possible that biases are being introduced but there are also analyses that rely on the typically high accuracy of imputation to improve less accurate genotype calls [[22,23]](https://paperpile.com/c/qIvJZ3/EYNRY+XvzGL). Moreover, we did not find a systematic enrichment for 1000 Genomes populations in the chunk copying vectors obtained for imputed samples and when comparing fineSTRUCTURE clusters we observe only minor differences which do not alter the results of fineSTRUCTURE significantly. We note, however, that some bias towards the reference using D-statistics on S5 Text (Section 5.3).

Most of research articles that analysed low-coverage ancient DNA relied on the haploidization of genotypes, resulting in the loss of vital information to detect population structure. Imputation offers a way of using haplotype-based methods to obtain diploid genotypes from low-coverage, degraded ancient DNA samples with low error rates and with little bias. As ancient DNA expands from a mostly Eurasian focus, where imputation provides encouraging results, it is yet uncertain how well will this methods perform in the presence of ancient populations distant from the ones represented in haplotype reference data.

# References

1. [Danecek P, Auton A, Abecasis G, Albers CA, Banks E, DePristo MA, et al. The variant call format and VCFtools. Bioinformatics. 2011;27: 2156–2158.](http://paperpile.com/b/qIvJZ3/pcBQg)

2. [Lawson DJ, Hellenthal G, Myers S, Falush D. Inference of population structure using dense haplotype data. PLoS Genet. 2012;8: e1002453.](http://paperpile.com/b/qIvJZ3/0G6gp)

3. [Jones ER, Gonzalez-Fortes G, Connell S, Siska V, Eriksson A, Martiniano R, et al. Upper Palaeolithic genomes reveal deep roots of modern Eurasians. Nat Commun. 2015;6: 8912.](http://paperpile.com/b/qIvJZ3/0zKib)

4. [Fu Q, Posth C, Hajdinjak M, Petr M, Mallick S, Fernandes D, et al. The genetic history of Ice Age Europe. Nature. 2016; doi:](http://paperpile.com/b/qIvJZ3/jWJGL)[10.1038/nature17993](http://dx.doi.org/10.1038/nature17993)

5. [Lazaridis I, Patterson N, Mittnik A, Renaud G, Mallick S, Kirsanow K, et al. Ancient human genomes suggest three ancestral populations for present-day Europeans. Nature. Nature Publishing Group, a division of Macmillan Publishers Limited. All Rights Reserved.; 2014;513: 409–413.](http://paperpile.com/b/qIvJZ3/epvLT)

6. [Haak W, Lazaridis I, Patterson N, Rohland N, Mallick S, Llamas B, et al. Massive migration from the steppe was a source for Indo-European languages in Europe. Nature. 2015; doi:](http://paperpile.com/b/qIvJZ3/tdKbB)[10.1038/nature14317](http://dx.doi.org/10.1038/nature14317)

7. [Malmström H, Gilbert MTP, Thomas MG, Brandström M, Storå J, Molnar P, et al. Ancient DNA reveals lack of continuity between neolithic hunter-gatherers and contemporary Scandinavians. Curr Biol. 2009;19: 1758–1762.](http://paperpile.com/b/qIvJZ3/8K0yp)

8. [Gamba C, Jones ER, Teasdale MD, McLaughlin RL, Gonzalez-Fortes G, Mattiangeli V, et al. Genome flux and stasis in a five millennium transect of European prehistory. Nat Commun. 2014;5: 5257.](http://paperpile.com/b/qIvJZ3/haBmu)

9. [Olalde I, Schroeder H, Sandoval-Velasco M, Vinner L, Lobón I, Ramirez O, et al. A Common Genetic Origin for Early Farmers from Mediterranean Cardial and Central European LBK Cultures. Mol Biol Evol. 2015;32: 3132–3142.](http://paperpile.com/b/qIvJZ3/8oCuH)

10. [Cunliffe B. Europe between the Oceans 9000 BC–AD 1000. New Haven-London. researchgate.net; 2008; Available:](http://paperpile.com/b/qIvJZ3/Y99ha) <https://www.researchgate.net/profile/Jesper_Boldsen/publication/227376643_Barry_Cunliffe/links/540d975f0cf2d8daaacb4e8b.pdf>

11. [Cassidy LM, Martiniano R, Murphy EM, Teasdale MD, Mallory J, Hartwell B, et al. Neolithic and Bronze Age migration to Ireland and establishment of the insular Atlantic genome. Proc Natl Acad Sci U S A. 2016;113: 368–373.](http://paperpile.com/b/qIvJZ3/j3m0l)

12. [Allentoft ME, Sikora M, Sjögren K-G, Rasmussen S, Rasmussen M, Stenderup J, et al. Population genomics of Bronze Age Eurasia. Nature. 2015;522: 167–172.](http://paperpile.com/b/qIvJZ3/fgJeD)

13. [Martiniano R, Caffell A, Holst M, Hunter-Mann K, Montgomery J, Müldner G, et al. Genomic signals of migration and continuity in Britain before the Anglo-Saxons. Nat Commun. 2016;7: 10326.](http://paperpile.com/b/qIvJZ3/V2Ck2)

14. [Hellenthal G, Busby GBJ, Band G, Wilson JF, Capelli C, Falush D, et al. A genetic atlas of human admixture history. Science. 2014;343: 747–751.](http://paperpile.com/b/qIvJZ3/mxZR3)

15. [Delaneau O, Zagury J-F, Marchini J. Improved whole-chromosome phasing for disease and population genetic studies. Nat Methods. 2013;10: 5–6.](http://paperpile.com/b/qIvJZ3/ZAlmX)

16. [Leslie S, Winney B, Hellenthal G, Davison D, Boumertit A, Day T, et al. The fine-scale genetic structure of the British population. Nature. 2015;519: 309–314.](http://paperpile.com/b/qIvJZ3/zVXBr)

17. [van Dorp L, Balding D, Myers S, Pagani L, Tyler-Smith C, Bekele E, et al. Evidence for a Common Origin of Blacksmiths and Cultivators in the Ethiopian Ari within the Last 4500 Years: Lessons for Clustering-Based Inference. PLoS Genet. 2015;11: e1005397.](http://paperpile.com/b/qIvJZ3/o0rx6)

18. [Broushaki F, Thomas MG, Link V, López S, van Dorp L, Kirsanow K, et al. Early Neolithic genomes from the eastern Fertile Crescent. Science. American Association for the Advancement of Science; 2016; aaf7943.](http://paperpile.com/b/qIvJZ3/KFIX1)

19. [Busby GB, Band G, Si Le Q, Jallow M, Bougama E, Mangano VD, et al. Admixture into and within sub-Saharan Africa. Elife. 2016;5. doi:](http://paperpile.com/b/qIvJZ3/3mLoy)[10.7554/eLife.15266](http://dx.doi.org/10.7554/eLife.15266)

20. [Breheny P, Burchett W. visreg: Visualization of regression models. R package version. 2012; 2–0.](http://paperpile.com/b/qIvJZ3/hKISs)

21. [Team RC. R: A language and environment for statistical computing. R Foundation for Statistical Computing, Vienna, Austria, 2012. ISBN 3-900051-07-0; 2014.](http://paperpile.com/b/qIvJZ3/0Rr6y)

22. [Nielsen R, Korneliussen T, Albrechtsen A, Li Y, Wang J. SNP calling, genotype calling, and sample allele frequency estimation from New-Generation Sequencing data. PLoS One. 2012;7: e37558.](http://paperpile.com/b/qIvJZ3/EYNRY)

23. [1000 Genomes Project Consortium, Abecasis GR, Altshuler D, Auton A, Brooks LD, Durbin RM, et al. A map of human genome variation from population-scale sequencing. Nature. 2010;467: 1061–1073.](http://paperpile.com/b/qIvJZ3/XvzGL)

**S8 Table – Coancestry matrix obtained with CHROMOPAINTER for the analysis including 67 ancient samples.**

**S9 Table – Coancestry matrix obtained with CHROMOPAINTER for the analysis of a dataset including 67 ancient samples and modern Eurasian genomes.**

**S12 Fig - Geographical and PC genetic coordinates for the Western_HG1 cluster.**

**S13 Fig - Geographical and PC genetic coordinates for the Western_HG2 cluster.**

**S14 Fig - Geographical and PC genetic coordinates for the fineSTRUCTURE Scandinavian_HG cluster.**

**S15 Fig - Geographical and PC genetic coordinates for the fineSTRUCTURE cluster Caucasus Hunter-gatherers.**

**S16 Fig - Geographical and PC genetic coordinates for the fineSTRUCTURE AegeanEN_HungarianLBK cluster.**

**S17 Fig - Geographical and PC genetic coordinates for the fineSTRUCTURE HungarianMLN_SpainCardialEN cluster.**

**S18 Fig - Geographical and PC genetic coordinates for the fineSTRUCTURE Atlantic_Neolithic cluster.**

**S19 Fig - Geographical and PC genetic coordinates for the fineSTRUCTURE Yamnaya_Afanasievo cluster.**

**S20 Fig - Geographical and PC genetic coordinates for the fineSTRUCTURE Sintashta_Andronovo cluster.**

**S21 Fig - Geographical and PC genetic coordinates for the fineSTRUCTURE CopperAge_to_AngloSaxon cluster.**

**S22 Fig - Geographical and PC genetic coordinates for the fineSTRUCTURE Hungary_BA cluster.**

**S23 Fig - Geographical and PC genetic coordinates for the fineSTRUCTURE Portugal_BA cluster.**

**S24 Fig - Geographical and PC genetic coordinates for the fineSTRUCTURE Russia_LBA cluster.**

**S25 Fig - Geographical and PC genetic coordinates for the fineSTRUCTURE Russia_LBA_IA cluster.**

**S26 Fig - Comparison between (A) unlinked and (B) linked CHROMOPAINTER/fineSTRUCTURE analyses.**

The unlinked analysis is only able to identify 10 populations, 9 less than when incorporating the linkage model.

**S27 Fig** - **CHROMOPAINTER haplotype donation vectors between each one of the imputed and non-imputed samples**

(A) Correlation between imputed and non-imputed median haplotype donation from sample BR2 (1), Loschbour (2) and LBK (3). (B) Normal Quantile-Quantile plots and outlier detection (labelled populations). Coloured dots show populations present (red) or absent (black) in the 1000 Genomes reference haplotype dataset. (C) Barplots illustrating imputed (left) and non-imputed (right) median haplotype donation (light blue) and the difference between median haplotype donation per population (dark blue).

**S28 Fig - fineSTRUCTURE tree comparison between each one of the imputed and non-imputed samples (BR2, Loschbour and LBK).**

The position of aDNA samples (shown in red) is very similar in both analyses.
