# Supplementary material for: The population genomics of archaeological transition in west Iberia: Investigation of ancient substructure using imputation and haplotype-based methods
Source: PLoS Genet. 2017 Jul 27;13(7):e1006852. doi: 10.1371/journal.pgen.1006852 (PMC5531429; doi:10.1371/journal.pgen.1006852)
Supplement: S7 Text — (DOCX) [file pgen.1006852.s007.docx]

# **S7 Text**

# Analysis of polygenic traits

Rui Martiniano, Lara M Cassidy, Ros Ó'Maoldúin, Russell McLaughlin, Nuno M Silva, Licinio Manco, Daniel Fidalgo, Tania Pereira, Maria J Coelho, Miguel Serra, Joachim Burger, Rui Parreira, Elena Moran, Antonio C Valera, Eduardo Porfirio, Rui Boaventura, Ana M Silva, Daniel G Bradley

## 7. Analysis of polygenic traits

We took advantage of the availability of whole-genome diploid genotypes obtained through imputation to estimate polygenic risk scores (PRS) in 67 ancient DNA samples. We chose traits that are known to have a highly heritable component, such as height [[1,2]](https://paperpile.com/c/fW3mNz/dbbbR+P9XGz), pigmentation ([[3]](https://paperpile.com/c/fW3mNz/6LIaU), Anthropometric BMI [[4]](https://paperpile.com/c/fW3mNz/PFiZP) and T2D [[5]](https://paperpile.com/c/fW3mNz/fG4Mr). Polygenic risk scores were estimated using PLINK 1.9 using the “--score” flag, and “--q-score-range” to compare the effect of p-value filtering (unfiltered p>0 against p<0.001) were done to evaluate the robustness of signals observed. Polygenic scores were centered at the mean for the dataset (S29 Fig).

## **7.1 Height**

### **7.1.1 Height polygenic risk score estimation in ancient DNA samples**

We used the summary statistics from two GIANT studies on height: one containing beta values for 180 markers [[2]](https://paperpile.com/c/fW3mNz/P9XGz) and a later study [[1]](https://paperpile.com/c/fW3mNz/dbbbR), which provides polygenic risk for ~2.5 million SNPs. First, we filtered imputed calls with PLINK 1.9, removing SNPs not genotyped across all 67 ancient individuals. For the 2014 dataset, containing approximately 2.5 M SNPs, we passed the parameter “--q-score-range” to perform an unrestricted analysis in terms of p-values and one analysis including SNPs with p-values < 0.001 (S30 Fig).

Results are qualitatively similar for both p-value thresholds, with WHG1 and WHG2 groups having high polygenic height estimation, and Neolithic Aegean samples presenting lower values. When moving forward in time, a PRS increase the Middle and Late Neolithic/Chalcolithic, was observed, which could be associated with admixture with WHG. According to ref. [[6]](https://paperpile.com/c/fW3mNz/1UZgJ), Neolithic Iberian samples were subject to selection for lower height. We do not observe a reduction from genetic height from the 2 Anatolian Neolithic samples to the EN Cardial included in this analysis. However, possible caveats of our analysis are: 1) we only have 1 EN sample from Iberia; 2) we simply report polygenic scores and have not tried to detect signatures of selection as in Mathieson et al. ; 3) We used a different, denser dataset [[1]](https://paperpile.com/c/fW3mNz/dbbbR); 4) Unknown effects of population structure which may be affecting our results - the polygenic summary statistics for height were estimated on a European ancestry dataset. Nevertheless, according to our analysis, the Yamnaya present the highest scores for polygenic height and this could be consistent with the scenario proposed by Mathieson et al.

We then estimated polygenic risk score using the same dataset as Mathieson et al. (2015) - Lango et al (2010) - but without restricting for p-values or posterior genotype probabilities otherwise there would not be enough SNPs for analysis (S31 Fig). This analysis is less resolved, with ~180 SNPs instead of ~2.5 M, but nevertheless we observe that the Neolithic Aegean still present lower genetic height than the Early Neolithic Cardial Spanish CB13. However we do observe low genetic height for some, not all MN/LN samples, although this is inconclusive. One unambiguous result is the far superior genetic height presented by the Yamnaya.

### **7.1.2 Relationship between ancient sample ancestry in present-day populations and genetic height**

Following recent research on the topic of genetic height - [[6]](https://paperpile.com/c/fW3mNz/1UZgJ) and Iain Mathieson’s blog (<http://mathii.github.io/review/2015/10/21/selection-on-height-in-europe>), we investigated the correlation between ancient ancestry in present-day populations and height polygenic risk scores. To do this, we first calculated polygenic risk in Eurasian populations from the Human Origins dataset (Lazaridis 2014). This was followed by the estimation of the percentage ancestry of five ancient populations (EHG, CHG, WHG, Yamanya, Anatolian Neolithic) in the same dataset, which was done through the implementation of the F4 ratio method described in [[7]](https://paperpile.com/c/fW3mNz/Vr1Yf), using the Admixtools package (version 4.1). Two individuals (S10 Table) possessing the highest genomic coverage from each population were used in the test, which took the form f4(Mbuti, Ancient_Ind1; Modern_WEurasian, Dai)/f4(Mbuti, Ancient_Ind1; Ancient_Ind2, Dai). The resulting ‘percentage ancestries’ of 48 modern Eurasian populations for each ancestral population were then plotted against their height polygenic risk score (S32 Fig).

## **7.2 Skin pigmentation**

We used the summary statistics reported in [[3]](https://paperpile.com/c/fW3mNz/6LIaU) for SNPs at genes SLC24A5, TYR, APBA2 and SLC45A2 which were found to be associated with skin pigmentation in a population of Cape Verdeans. Highest genetic scores corresponding to darker pigmentation were observed for Western HG groups (S33 Fig), with much lower values (lighter pigmentation) observed for the Neolithic and subsequent time-periods. The transition from darker skin hunter-gatherers (e.g. [[8]](https://paperpile.com/c/fW3mNz/NDjij)) and lighter skin Europeans had been described [[6]](https://paperpile.com/c/fW3mNz/1UZgJ). Here we use the effect on pigmentation estimated collectively from 4 SNPs instead of 1 SNP independently. When applying this method to Russian LBA-IA populations, the results are highly variable and this is likely to be associated with variable amounts of East Asian ancestry. The same summary statistics have been used before to investigate signatures of selection in polygenic traits, where the authors observed that the pigmentation scores obtained in light skinned East Asian and American populations presented similar values to those observed in Africans, which was interpreted as a result of not having captured all the variants responsible for the phenotype of skin pigmentation across the different ancestries present at a global level [[9]](https://paperpile.com/c/fW3mNz/hT8Ue). The Cape Verdean population is admixed between African and European sources, and therefore will represent at least some part of the variants that modulate skin colour in populations with those ancestries, but not in East Asian individuals which most likely have different SNPs regulating this phenotype.

## **7.3 Body mass index (BMI)**

In order to estimate genetic risk scores for BMI in ancient samples (S34 Fig)we used summary statistics reported by [[4]](https://paperpile.com/c/fW3mNz/PFiZP) on a multi-ancestry cohort. Western HG 2 and Scandinavian HG presented higher genetic scores than Neolithic farmers with an increase in polygenic risk in Bronze Age populations. If true, this signal could potentially be interpreted as the effect of natural selection favoring higher BMI related alleles in hunter-gatherers because of their foraging mode of subsistence, and lower BMI alleles in the Neolithic, a period where crops and animal domestication would provide a stable source of nutrients. However, the high variability associated to these findings do not allow establishing a secure link between the scores obtained and changes in diet throughout prehistory. First, there is high variability according to which p-value thresholds are used (see for example, CHG), and second, [[9]](https://paperpile.com/c/fW3mNz/hT8Ue) observed intraregional differences in present-day human populations but it was difficult to interpret these because of lack of association with an ecological pattern.

## **7.4 Type 2 Diabetes (T2D)**

For T2D polygenic score estimation we used the summary statistics released by [[5]](https://paperpile.com/c/fW3mNz/fG4Mr) and calculated in a mostly European cohort. We converted odds ratio (OR) to effect size by taking the logarithm of OR/1.81 [[10]](https://paperpile.com/c/fW3mNz/q4xQC). We observed higher polygenic risk scores for HGs and Neolithic samples and a reduction in risk in Bronze Age populations (S35 Fig). Results here were less variable when using different p-value thresholds but within group variability makes the interpretation of these results difficult. In addition, recent work has shown variable results according to whether the summary statistics were estimated in a cohort with European or multi-ethnic ancestry [[11]](https://paperpile.com/c/fW3mNz/2LVjJ). Furthermore, in a study of modern samples, no correlation with geography was observed and scores did not exceed the expectations from population structure, and therefore do not provide evidence for selection as the driving force of these differences [[9]](https://paperpile.com/c/fW3mNz/hT8Ue)

# References

1. [Wood AR, Esko T, Yang J, Vedantam S, Pers TH, Gustafsson S, et al. Defining the role of common variation in the genomic and biological architecture of adult human height. Nat Genet. 2014;46: 1173–1186.](http://paperpile.com/b/fW3mNz/dbbbR)

2. [Lango Allen H, Estrada K, Lettre G, Berndt SI, Weedon MN, Rivadeneira F, et al. Hundreds of variants clustered in genomic loci and biological pathways affect human height. Nature. 2010;467: 832–838.](http://paperpile.com/b/fW3mNz/P9XGz)

3. [Beleza S, Johnson NA, Candille SI, Absher DM, Coram MA, Lopes J, et al. Genetic architecture of skin and eye color in an African-European admixed population. PLoS Genet. 2013;9: e1003372.](http://paperpile.com/b/fW3mNz/6LIaU)

4. [Locke AE, Kahali B, Berndt SI, Justice AE, Pers TH, Day FR, et al. Genetic studies of body mass index yield new insights for obesity biology. Nature. 2015;518: 197–206.](http://paperpile.com/b/fW3mNz/PFiZP)

5. [Morris AP, Voight BF, Teslovich TM, Ferreira T, Segrè AV, Steinthorsdottir V, et al. Large-scale association analysis provides insights into the genetic architecture and pathophysiology of type 2 diabetes. Nat Genet. 2012;44: 981–990.](http://paperpile.com/b/fW3mNz/fG4Mr)

6. [Mathieson I, Lazaridis I, Rohland N, Mallick S, Llamas B, Pickrell J, et al. Eight thousand years of natural selection in Europe [Internet]. 2015 Mar. doi:](http://paperpile.com/b/fW3mNz/1UZgJ)[10.1101/016477](http://dx.doi.org/10.1101/016477)

7. [Patterson N, Moorjani P, Luo Y, Mallick S, Rohland N, Zhan Y, et al. Ancient admixture in human history. Genetics. 2012;192: 1065–1093.](http://paperpile.com/b/fW3mNz/Vr1Yf)

8. [Olalde I, Allentoft ME, Sánchez-Quinto F, Santpere G, Chiang CWK, DeGiorgio M, et al. Derived immune and ancestral pigmentation alleles in a 7,000-year-old Mesolithic European. Nature. 2014;507: 225–228.](http://paperpile.com/b/fW3mNz/NDjij)

9. [Berg JJ, Coop G. A population genetic signal of polygenic adaptation. PLoS Genet. 2014;10: e1004412.](http://paperpile.com/b/fW3mNz/hT8Ue)

10. [Chinn S. A simple method for converting an odds ratio to effect size for use in meta-analysis. Stat Med. 2000;19: 3127–3131.](http://paperpile.com/b/fW3mNz/q4xQC)

11. [Martin AR, Gignoux CR, Walters RK, Wojcik GL, Gravel S, Daly MJ, et al. Population genetic history and polygenic risk biases in 1000 Genomes populations [Internet]. bioRxiv. 2016. p. 070797. doi:](http://paperpile.com/b/fW3mNz/2LVjJ)[10.1101/070797](http://dx.doi.org/10.1101/070797)

11. [Martin AR, et al. (2016) Population genetic history and polygenic risk biases in 1000 Genomes populations. *bioRxiv*:070797.](http://paperpile.com/b/fW3mNz/2LVjJ)

**S10 Table - List of ancient individuals used in the F4 ratio test.**

This table contains the individuals which were used to estimate the approximate percentage ancestry in modern populations of five ancestral groups who have contributed to western Eurasian variation, using an F4 ratio test (Patterson 2012).

**S29 Fig** **- Bar plots illustrating polygenic risk scores across time, estimated for each one of the ancient population clusters.**

The traits chosen were: A) Height; B) Pigmentation; C) BMI and D) T2D. Polygenic scores were centered at the mean for the dataset. As in Fig 1 in the main text, each cluster is represented with a different colour.

**S30 Fig - Polygenic risk scores estimated for height using genomewide summary statistics from the Wood 2014 dataset.**

(A) p=0 (B) p<0.001. SNPs with posterior genotype probability of less than 0.99 were excluded from analysis.

**S31 Fig - Polygenic risk scores estimated for height using genomewide summary statistics (Lango et al., 180 SNPs).**

**S32 Fig - Correlation between strands of ancestry and inferred polygenic risk score in present-day Europeans.**

Hunter-gatherer (WHG, EHG, CHG), Neolithic (Anatolian_EN) and Steppe (Yamnaya) Ancestry was measured by f4(Mbuti, Ancient_Ind1; Modern_WEurasian, Dai)/f4(Mbuti, Ancient_Ind1; Ancient_Ind2, Dai). Polygenic risk scores for height (92) were determined using ~280.000 SNPs in 48 European populations. Blue line presents the linear regression. Individual samples are represented by gray dots and larger coloured circles represent the mean genetic score for each population.

**S33 Fig - Height map and PCA.**

Red - increased genetic height scores, black - decreased genetic height. Broadly, hunter-gatherers and populations from Copper age and after present highest proportion of height increasing associated variants followed by Neolithic farmers.

**S34 Fig** **- Polygenic scores for pigmentation based on the SNPs reported in (94).**

SNPs with posterior genotype probability of less than 0.99 were excluded from analysis.

**S35 Fig** **- Polygenic risk scores estimated for BMI using genomewide summary statistics reported by (95) on a multi-ancestry cohort.**

(A) p=0 (B) p<0.001. SNPs with posterior genotype probability of less than 0.99 were excluded from analysis.

**S36 Fig - Polygenic risk scores estimated for T2D using genomewide summary statistics reported by (96).**

A) p=0 B) p<0.001. SNPs with posterior genotype probability of less than 0.99 were excluded from analysis.
