# Supplementary material for: The population genomics of archaeological transition in west Iberia: Investigation of ancient substructure using imputation and haplotype-based methods
Source: PLoS Genet. 2017 Jul 27;13(7):e1006852. doi: 10.1371/journal.pgen.1006852 (PMC5531429; doi:10.1371/journal.pgen.1006852)
Supplement: S8 Text — (DOCX) [file pgen.1006852.s008.docx]

# **S8 Text**

# Extended Haplotype Homozygosity Analysis

Rui Martiniano, Lara M Cassidy, Ros Ó'Maoldúin, Russell McLaughlin, Nuno M Silva, Licinio Manco, Daniel Fidalgo, Tania Pereira, Maria J Coelho, Miguel Serra, Joachim Burger, Rui Parreira, Elena Moran, Antonio C Valera, Eduardo Porfirio, Rui Boaventura, Ana M Silva, Daniel G Bradley

## 8. Extended Haplotype Homozygosity Analysis

Selscan [[1]](https://paperpile.com/c/IrZQ35/j7ioV) was used to investigate extended haplotype homozygosity (EHH) around SNPs of interest previously described in ref. [[2]](https://paperpile.com/c/IrZQ35/y62o0): LCT (rs4988235), SLC24A5 (rs1426654), SLC45A2 (rs16891982), HERC2 (rs12913832), EDAR (rs3827760) and FADS1 (rs174546). First, SNPs within 10 Mb of each SNP were included for analysis, removing SNPs which are multiallelic and with multiple physical coordinates. EHH requires large populations, and therefore selscan in 3 groups: HG, Neolithic farmers and Copper Age to Anglo-Saxon, using the --ehh and --keep-low-freq flag. Additionally, to maximize the number of overlapping SNPs across all samples, we did not impose a cutoff at the level of posterior genotype probability.

The effect of strong positive selection can be detected using long-range haplotype methods. When new variants are subject to weak selective forces, they usually take a long time to reach high frequencies in a population, and therefore, recombination would have had enough time to break down the haplotype surrounding them. Conversely, alleles under strong selection (and the haplotypes which contain them) will rapidly increase in frequency in a population [[3]](https://paperpile.com/c/IrZQ35/3RJT8). Extended haplotype homozygosity (EHH) makes use of LD decay to infer events of recent positive selection by estimating the probability of homozygosity at a certain base across 2 randomly chosen chromosomes in a population. EHH was applied to the dataset of ancient samples to variants previously described as under selection [[2]](https://paperpile.com/c/IrZQ35/y62o0) (S37 Fig). This method is typically applied to large datasets and therefore, the data was divided into 3 populations - HG, Neolithic farmers and Copper Age to Anglo-Saxon - instead of the 19 clusters obtained with fineSTRUCTURE.

EHH analysis of the region around the LCT SNP rs4988235 revealed larger haplotypes in the “Copper Age to Anglo Saxon” group than in present-day individuals, consistent with recent positive selection and subsequent decay of linkage disequilibrium (LD) from the selected SNP with time. The first appearance of the derived allele in our dataset was in 3 Russian BA samples (2 Karasuk and 1 from Mezhovskaya cultures), but also in a higher coverage Irish BA, as reported in [[4]](https://paperpile.com/c/IrZQ35/ZX3bq) and in 2 samples from Roman Britain, but not in the Neolithic, as expected [[2,5]](https://paperpile.com/c/IrZQ35/y62o0+BFh5g). Portuguese MBA samples presented the ancestral allele at this locus, suggesting that the ability to digest milk in this region might have only appeared at later stage, when compared to Central and NW Europe. While the frequency of the selected LCT allele in Bronze Age Europeans was relatively low (approximately 10% [[5]](https://paperpile.com/c/IrZQ35/BFh5g)), the appearance of homozygous genotypes in the Roman and Anglo-Saxon samples points to an increase of frequency during the Iron Age. Accordingly, this variant was absent in the Middle Bronze Age samples from Portugal.

The earliest appearances of a derived allele at SLC24A5 variant rs1426654, associated with skin pigmentation [[6]](https://paperpile.com/c/IrZQ35/WgtxL), were in a WHG (Hungarian KO1), as described previously [[7]](https://paperpile.com/c/IrZQ35/tFqy7) and in a SHG (Motala12), as observed in [[2]](https://paperpile.com/c/IrZQ35/y62o0). The EHH analysis shows a dramatic increase in frequency during the Neolithic with large regions of homozygosity, indicating strong selection. A weaker signal is observed for rs16891982 at the SLC45A2 gene, which appears for the first time in Motala12 (SHG) and despite having been described as being strongly selected [[2]](https://paperpile.com/c/IrZQ35/y62o0), the EHH profile does not suggest an extensive sweep at this locus.

FADS1 locus rs174546, associated with low triglyceride levels and reported as being under strong selection [[2]](https://paperpile.com/c/IrZQ35/y62o0), is ancestral in HG, emerging with shift to agriculture. Change in diet could have to have created a selection of moderate strength for the derived allele as suggested by larger haplotypes in the EHH analysis.

Considerable EHH surrounding the EDAR SNPs rs3827760 consistent with reports of selection [[8]](https://paperpile.com/c/IrZQ35/WMHkH). It appears as first as heterozygous in SHG Motala12, as reported in [[2]](https://paperpile.com/c/IrZQ35/y62o0), to reemerge in the Iron Age Karasuk, Andronovo and Kytmanovo. One other Karasuk sample (RISE497) presented homozygous genotype at this SNP. These findings corroborate an earlier appearance of the derived allele and later reintroduction in Europe probably associated with East Asian-related ancestries.

The derived allele at HERC2 locus rs12913832, associated with eye colour [[9]](https://paperpile.com/c/IrZQ35/xkhYG), was prevalent in HGs, however, because of the low number of samples it is impossible to make reliable inferences regarding selection in the present analysis. It was identified at high frequency in WHG, including SHG (0.75-1), with a decrease in frequency in the Neolithic clusters (0.21-0.5), particularly in the Atlantic Neolithic cluster, also observed by [[10]](https://paperpile.com/c/IrZQ35/UoHYC), to which MN/LN Portuguese samples belong (only one sample has derived alleles at this SNP). However, an increase in Portuguese Bronze Age (3 derived alleles in 2 samples from Torre Velha 3 burial site), which is also observed in the “European LN-AS” and “Sintashta_Andronovo” populations, but none in the “Yamnaya_Afanasievo” cluster. The observation that HG had blue eyes was first made by Olalde (Derived immune and ancestral pigmentation alleles in a 7,000-year-old Mesolithic European). CB13, Cardial Neolithic Spanish, previously undescribed, is heterozygous.

We note that haplotype-based methods such as EHH are highly dependent on phasing accuracy as well as on rare and low frequency variation. In the present work we demonstrated that rare/low frequency variants do not impute accurately in ancient samples, which is expected considering that these variants are not well represented in the modern population reference haplotype dataset used for imputation. Therefore, it is possible that our analysis has overestimated homozygosity due to this inability to detect low frequency variation. Furthermore, phasing accuracy in ancient samples is still poorly understood. With this in mind, our analyses presented here should be considered experimental. Lastly, model-based approaches that use allele frequency changes across time, such as for example the method recently published by reference [11], may provide a more statistically robust means of determining the timing of selection than qualitative inferences from the observation of EHH. The application of these methodologies should be the focus of future analyses.

# References

1. [Szpiech ZA, Hernandez RD. selscan: an efficient multithreaded program to perform EHH-based scans for positive selection. Mol Biol Evol. 2014;31: 2824–2827.](http://paperpile.com/b/IrZQ35/j7ioV)

2. [Mathieson I, Lazaridis I, Rohland N, Mallick S, Llamas B, Pickrell J, et al. Eight thousand years of natural selection in Europe [Internet]. 2015 Mar. doi:](http://paperpile.com/b/IrZQ35/y62o0)[10.1101/016477](http://dx.doi.org/10.1101/016477)

3. [Sabeti PC, Reich DE, Higgins JM, Levine HZP, Richter DJ, Schaffner SF, et al. Detecting recent positive selection in the human genome from haplotype structure. Nature. 2002;419: 832–837.](http://paperpile.com/b/IrZQ35/3RJT8)

4. [Cassidy LM, Martiniano R, Murphy EM, Teasdale MD, Mallory J, Hartwell B, et al. Neolithic and Bronze Age migration to Ireland and establishment of the insular Atlantic genome. Proc Natl Acad Sci U S A. 2016;113: 368–373.](http://paperpile.com/b/IrZQ35/ZX3bq)

5. [Allentoft ME, Sikora M, Sjögren K-G, Rasmussen S, Rasmussen M, Stenderup J, et al. Population genomics of Bronze Age Eurasia. Nature. 2015;522: 167–172.](http://paperpile.com/b/IrZQ35/BFh5g)

6. [Beleza S, Johnson NA, Candille SI, Absher DM, Coram MA, Lopes J, et al. Genetic architecture of skin and eye color in an African-European admixed population. PLoS Genet. 2013;9: e1003372.](http://paperpile.com/b/IrZQ35/WgtxL)

7. [Gamba C, Jones ER, Teasdale MD, McLaughlin RL, Gonzalez-Fortes G, Mattiangeli V, et al. Genome flux and stasis in a five millennium transect of European prehistory. Nat Commun. 2014;5: 5257.](http://paperpile.com/b/IrZQ35/tFqy7)

8. [Bryk J, Hardouin E, Pugach I, Hughes D, Strotmann R, Stoneking M, et al. Positive Selection in East Asians for an EDAR Allele that Enhances NF-κB Activation. PLoS One. Public Library of Science; 2008;3: e2209.](http://paperpile.com/b/IrZQ35/WMHkH)

9. [Sturm RA, Duffy DL, Zhao ZZ, Leite FPN, Stark MS, Hayward NK, et al. A single SNP in an evolutionary conserved region within intron 86 of the HERC2 gene determines human blue-brown eye color. Am J Hum Genet. 2008;82: 424–431.](http://paperpile.com/b/IrZQ35/xkhYG)

10. [Günther T, Valdiosera C, Malmström H, Ureña I, Rodriguez-Varela R, Sverrisdóttir ÓO, et al. Ancient genomes link early farmers from Atapuerca in Spain to modern-day Basques. Proc Natl Acad Sci U S A. 2015;112: 11917–11922.](http://paperpile.com/b/IrZQ35/UoHYC)

11. [Schraiber JG, Evans SN, Slatkin M. Bayesian Inference of Natural Selection from Allele Frequency Time Series. Genetics. 2016;203: 493–511.](http://paperpile.com/b/IrZQ35/DOlz)

**S37 Fig - Extended haplotype homozygosity (EHH) in regions under selection.**

Panels on the left represent the decay of EHH, or the probability of homozygosity at a certain base across 2 randomly chosen chromosomes in a population. Plots on the right represent existing haplotypes in a population, with the lower portion of the graph depicting haplotypes with the derived allele (red) and the upper part showing haplotypes carrying the ancestral allele (blue). Unique haplotypes in a population are not represented. Legend: CEU - Utah Residents (CEPH) with Northern and Western Ancestry; YRI - Yoruba in Ibadan, Nigeria; CHB - Han Chinese in Beijing, China; 1KG: 1000 Genomes Project. * Earliest appearance of the homozygous derived allele in the samples analysed.
