# Supplementary figures and images for: The population genomics of archaeological transition in west Iberia: Investigation of ancient substructure using imputation and haplotype-based methods
Source: PLoS Genet. 2017 Jul 27;13(7):e1006852. doi: 10.1371/journal.pgen.1006852 (PMC5531429; doi:10.1371/journal.pgen.1006852)

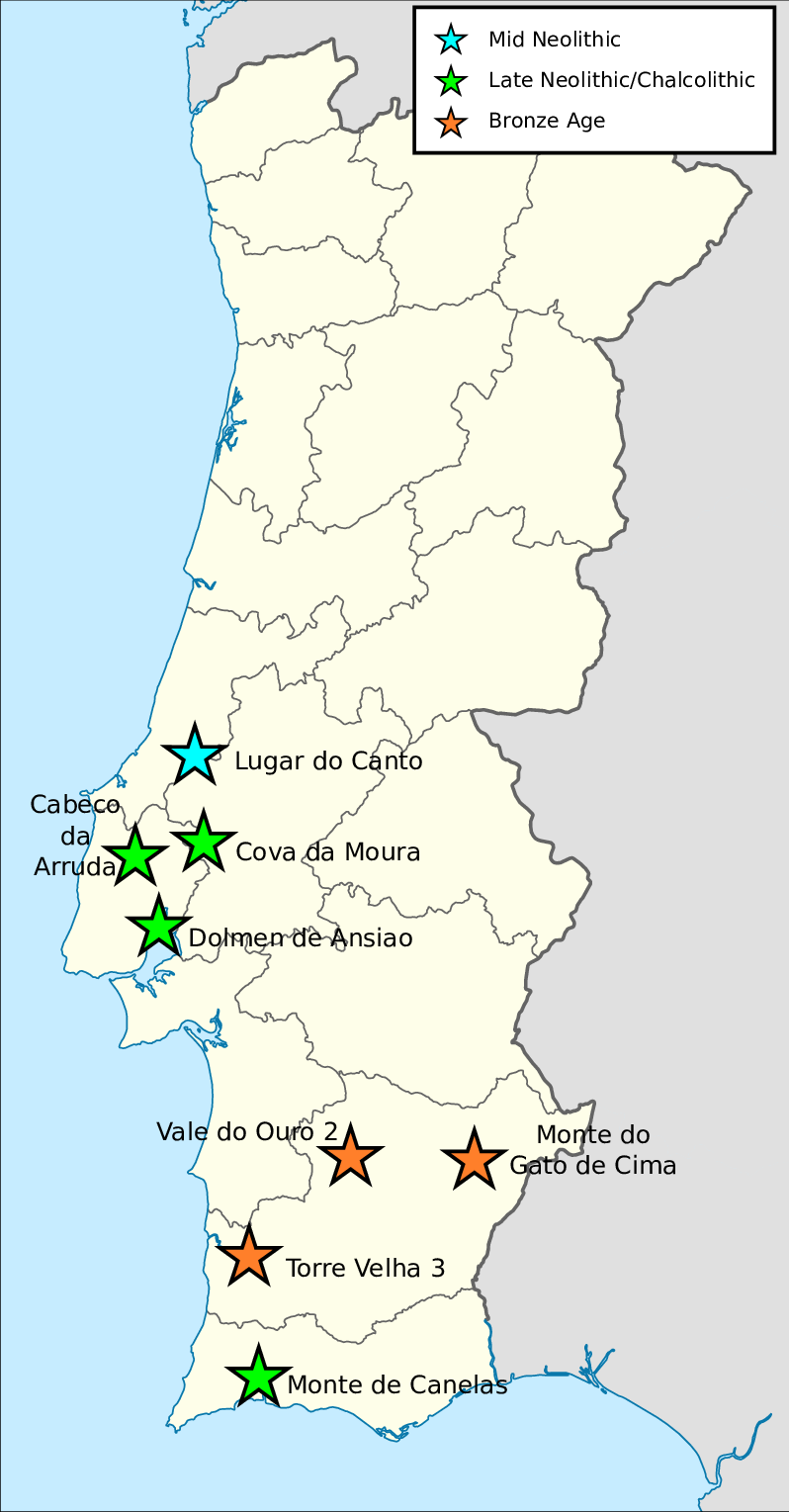

Supplement: S1 Fig — (TIF) [file pgen.1006852.s019.tif]

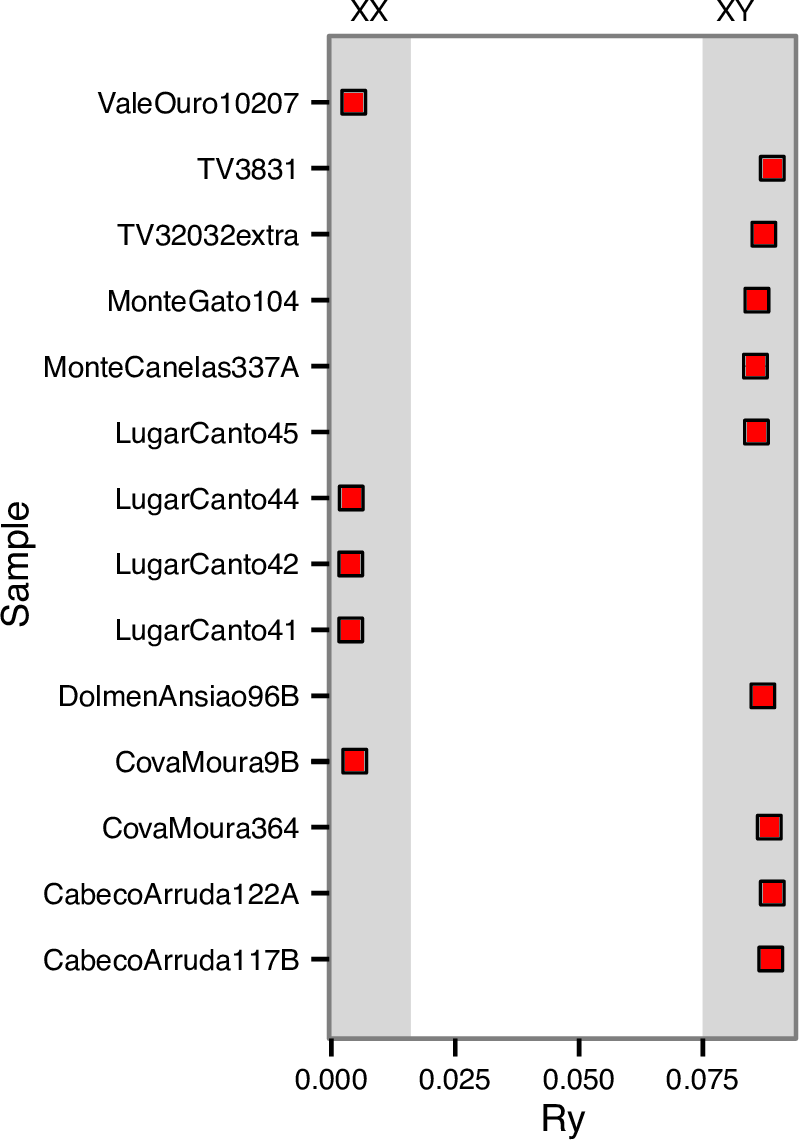

Supplement: S2 Fig — (TIF) [file pgen.1006852.s020.tif]

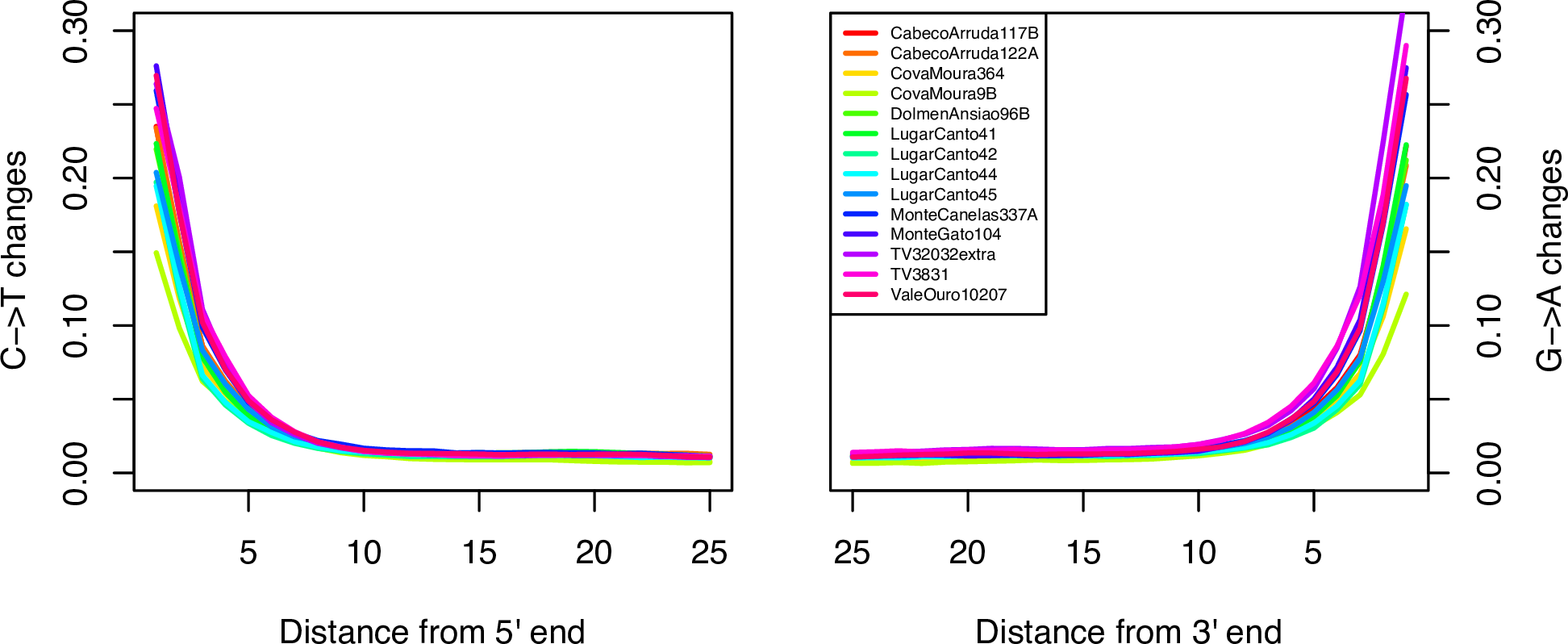

Supplement: S3 Fig — (TIF) [file pgen.1006852.s021.tif]

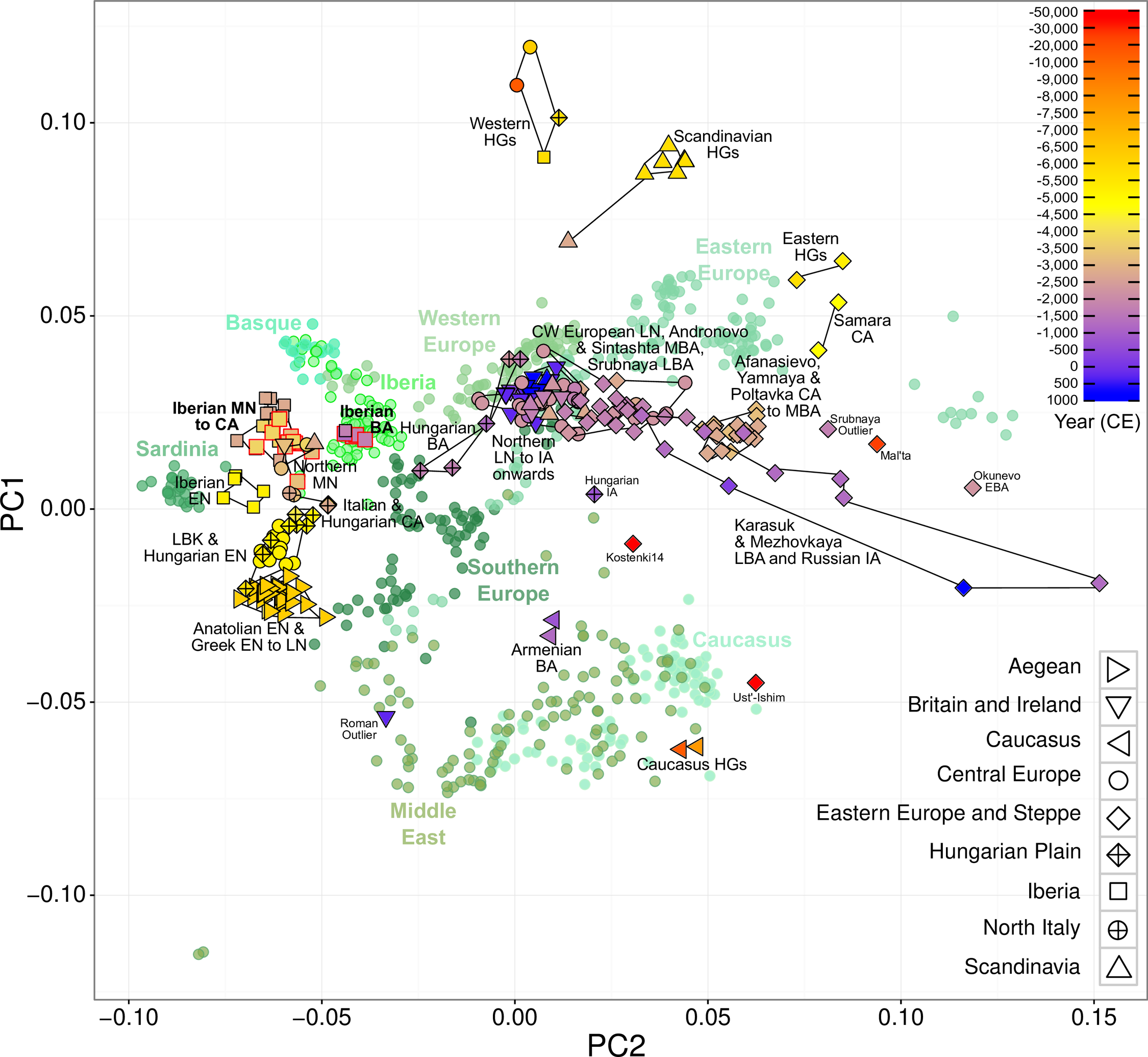

Supplement: S4 Fig — The analysis is based on approximately 600,000 SNP positions. Moderns samples from the Human Origins dataset are represented in greyscale, with the exception of modern Iberians shown in green. Ancient samples are coloured by time depth and shaped according to geographic region. Ancient individuals from Portugal are outlined in red. (TIF) [file pgen.1006852.s022.tif]

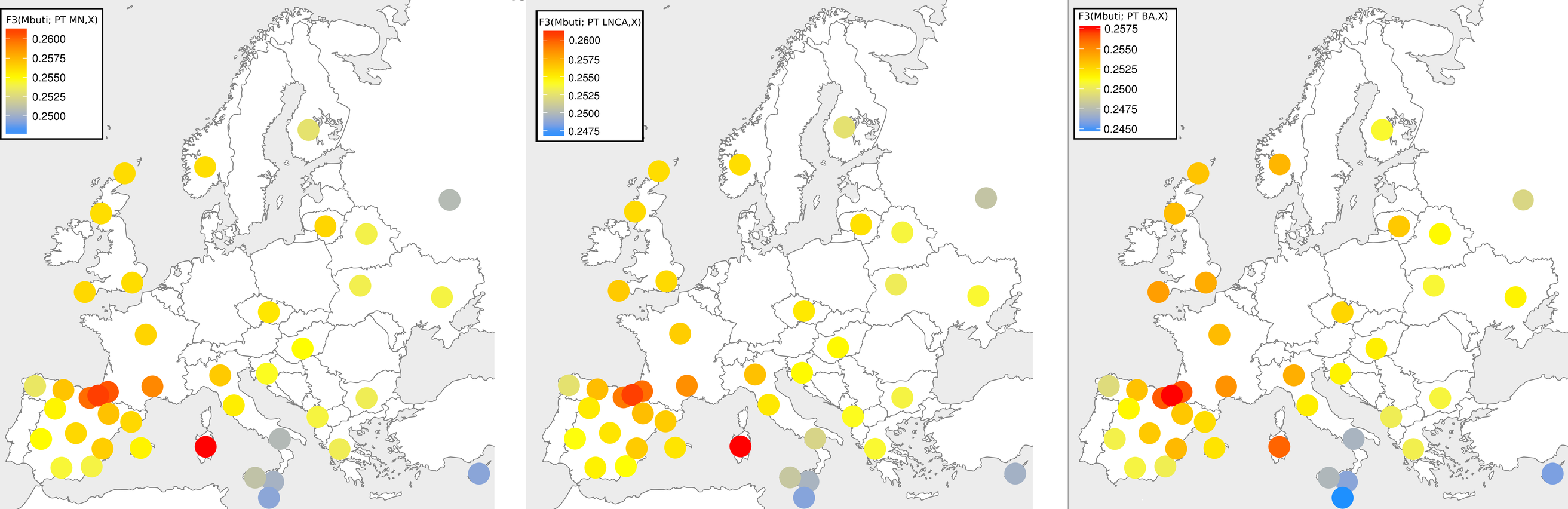

Supplement: S5 Fig — (TIF) [file pgen.1006852.s023.tif]

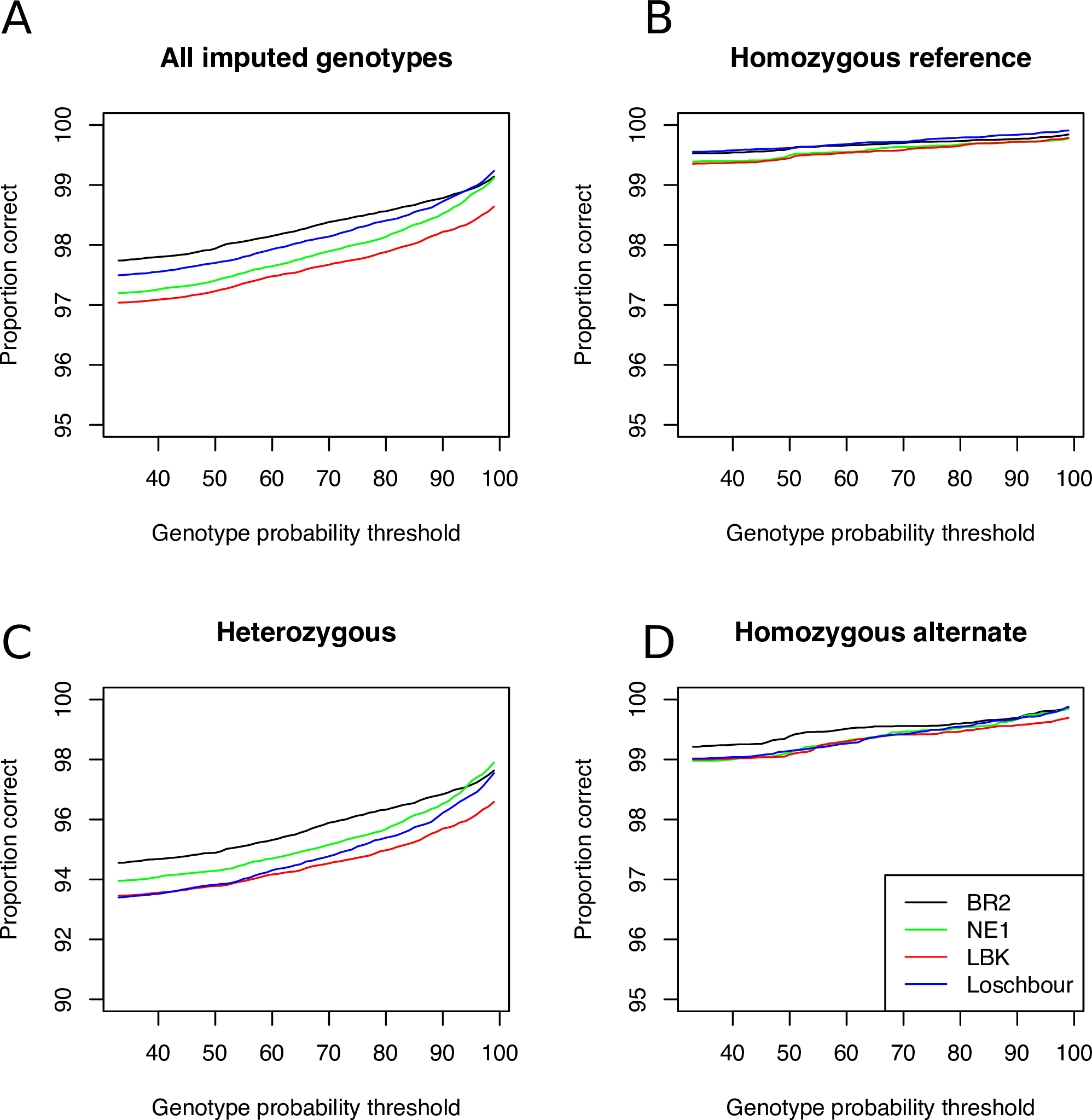

Supplement: S6 Fig — Comparison of variant calls obtained for BR2, NE1, Loschbour and Stuttgart at full coverage with genotypes from the same 4 individuals downsampled to 2x and subsequently imputed. Accuracy in (A) all 3 types of genotypes; (B) homozygous reference; (C) heterozygous and (D) homozygous alternate. (TIF) [file pgen.1006852.s024.tif]

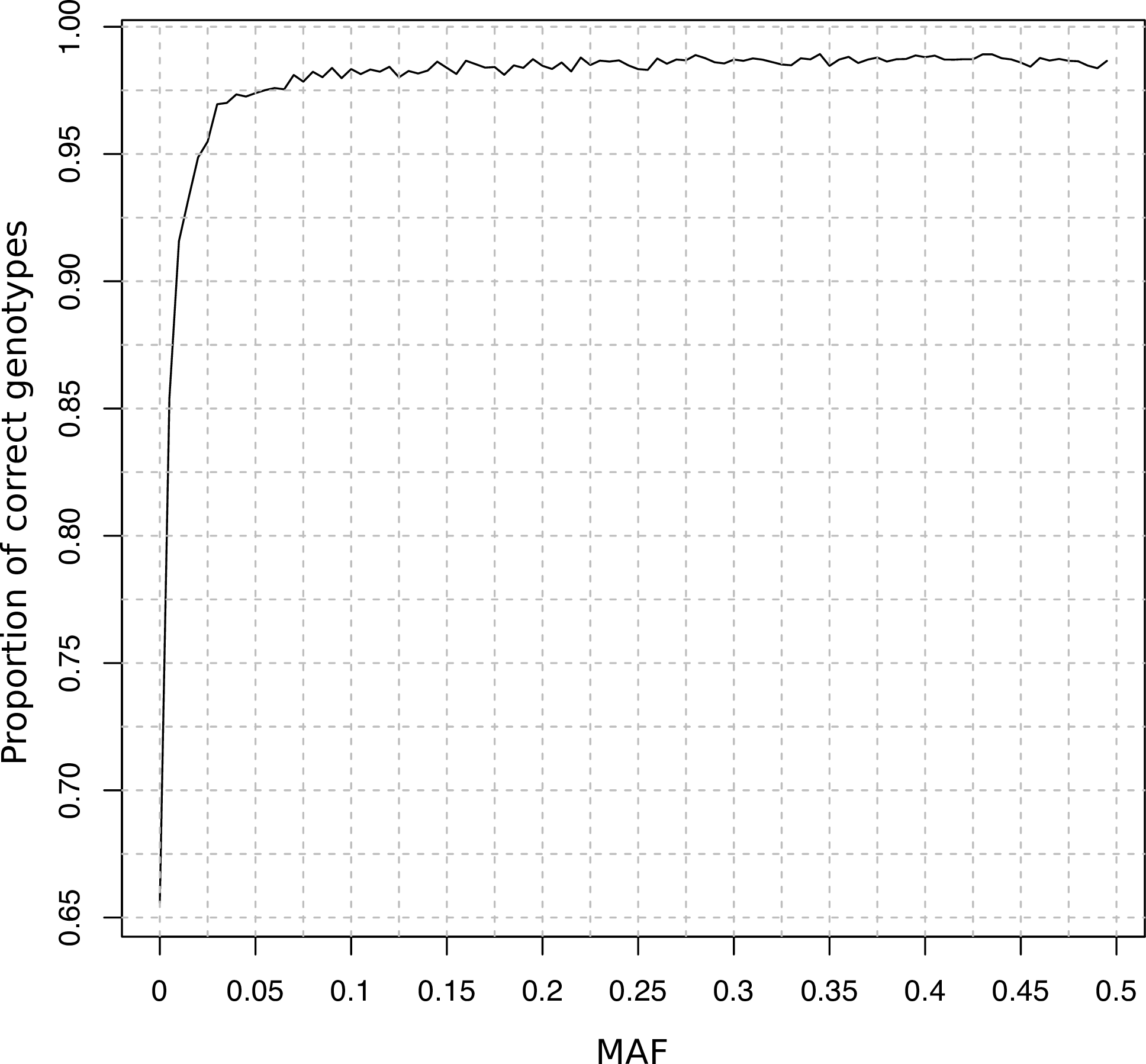

Supplement: S7 Fig — In this analysis, imputed genotypes were filtered by post imputation genotype probability ⋝ 0.99. (TIF) [file pgen.1006852.s025.tif]

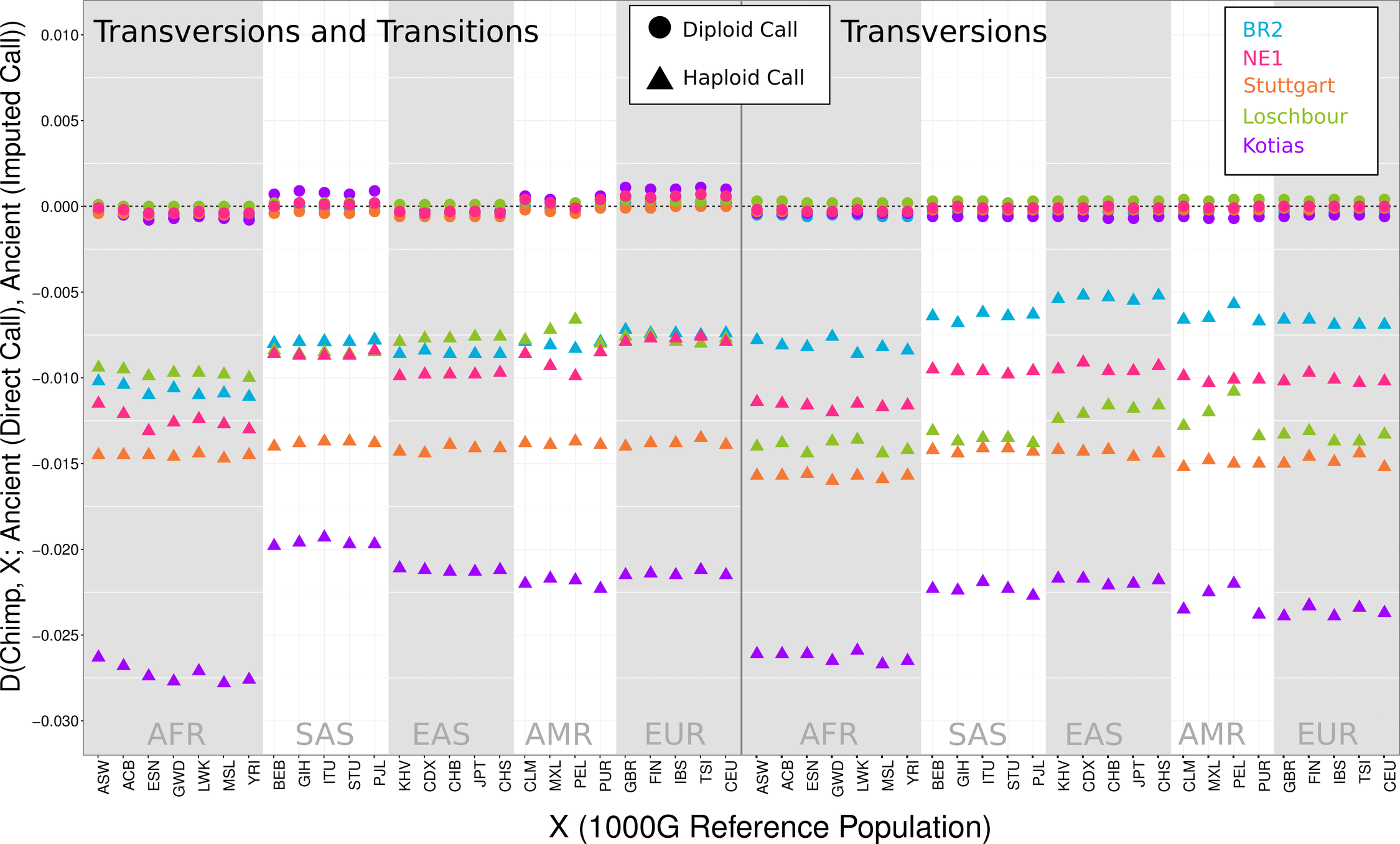

Supplement: S8 Fig — Results are shown for both all sites and just transversions in two separate panels. A world minor allele frequency of 25% has been applied. 1000 Genomes population and superpopulation names are noted along the X axis. (TIF) [file pgen.1006852.s026.tif]

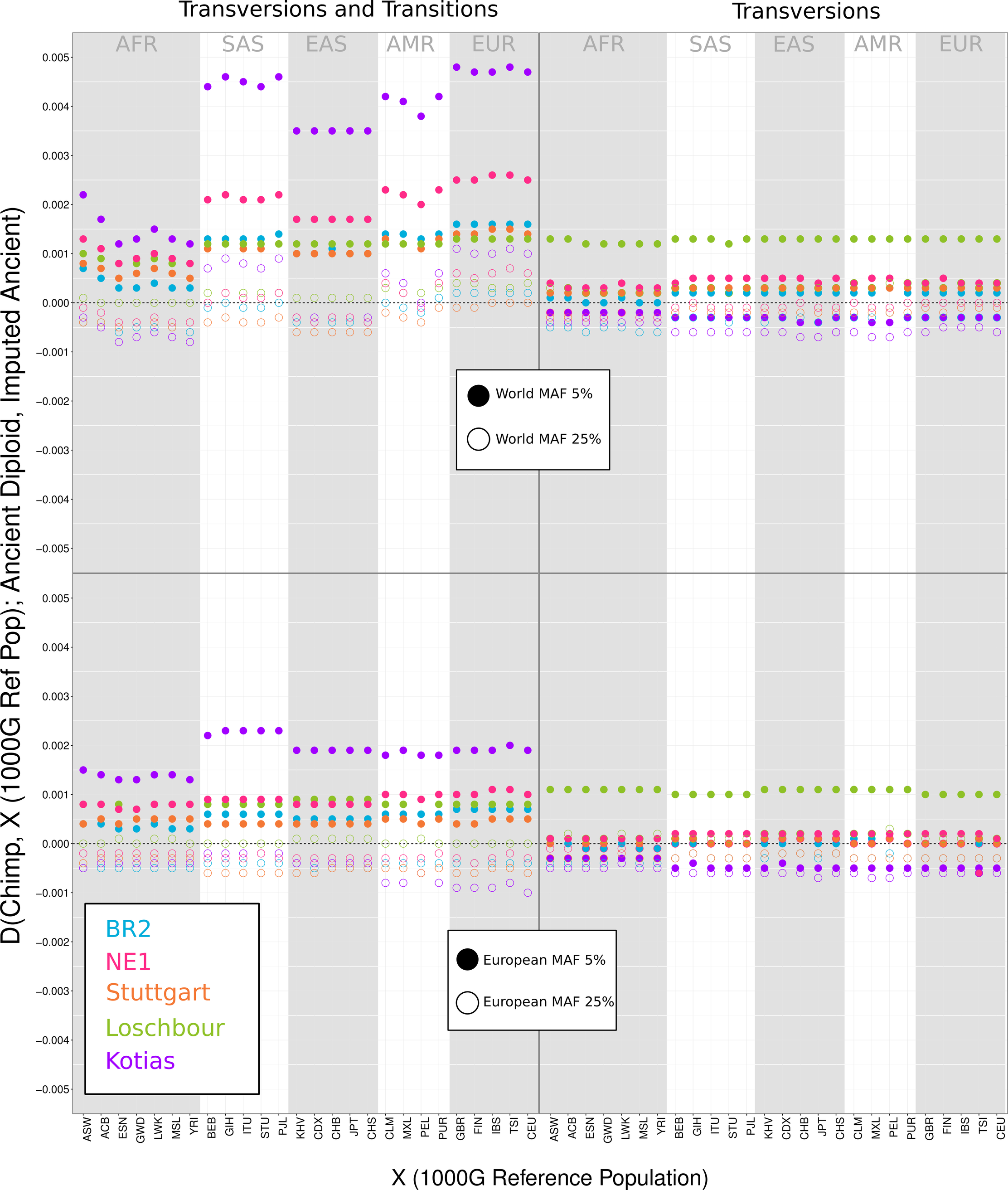

Supplement: S9 Fig — Results are shown for both all sites and just transversions in on left hand and right hand panels respectively. Top panels display world MAF filters of 25% and 5%. Bottom panels display European MAF filters of 25% and 5%. 1000 Genomes population and superpopulation names are noted along the X axis. (TIF) [file pgen.1006852.s027.tif]

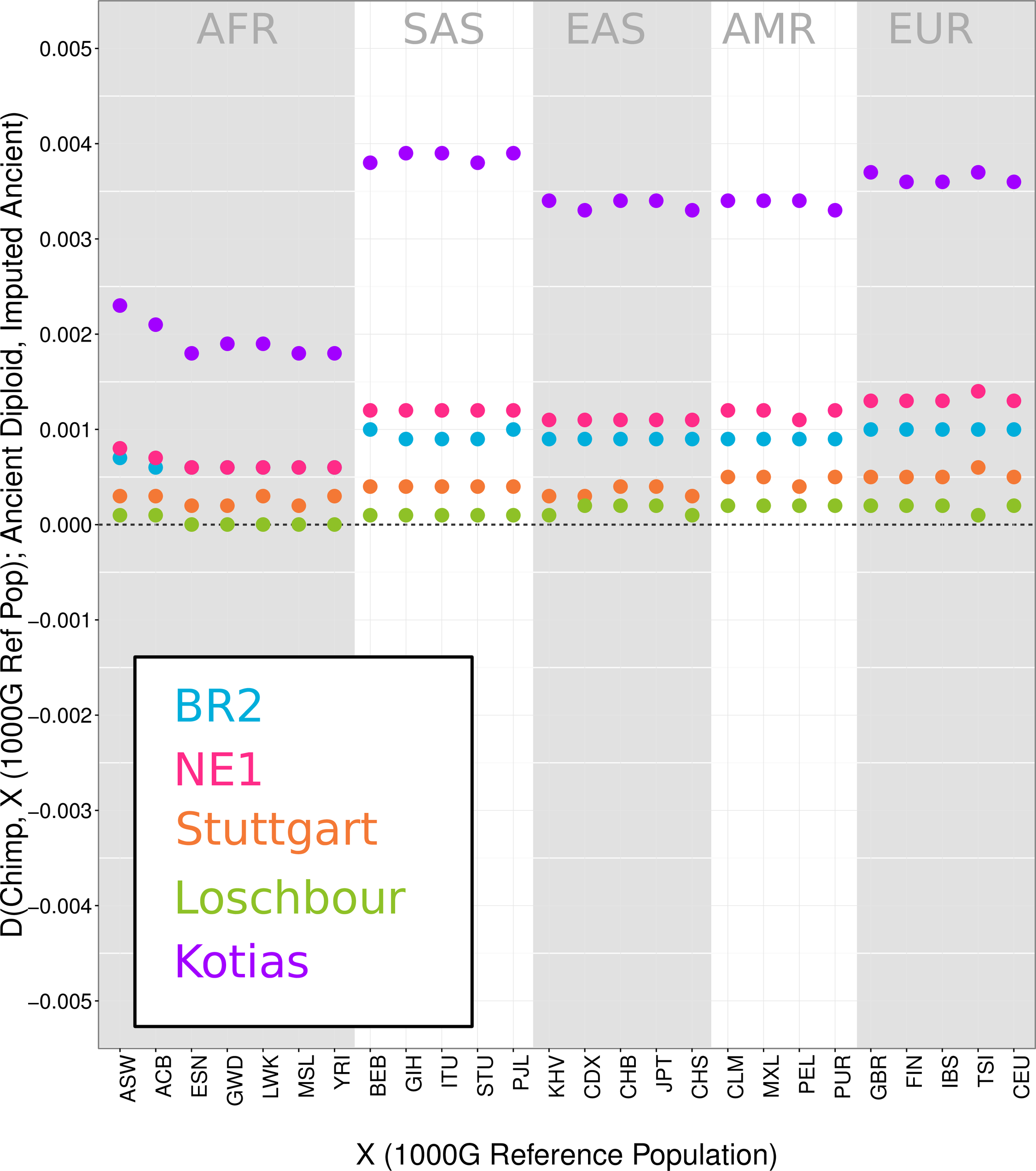

Supplement: S10 Fig — (TIF) [file pgen.1006852.s028.tif]

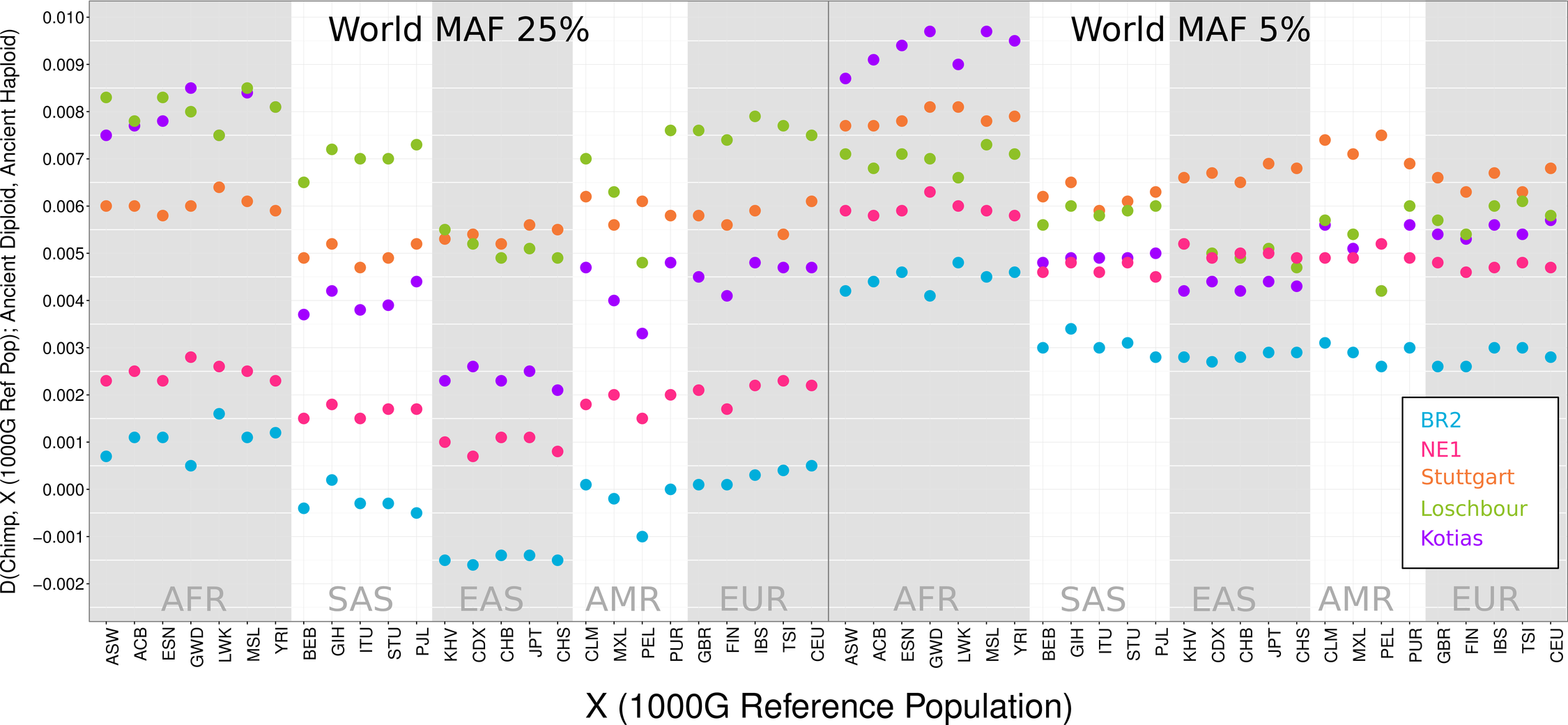

Supplement: S11 Fig — Results are shown for world MAF filters of 25% and 5%. Only transversion SNPs are considered. 1000 Genomes population and superpopulation names are noted along the X axis. (TIF) [file pgen.1006852.s029.tif]

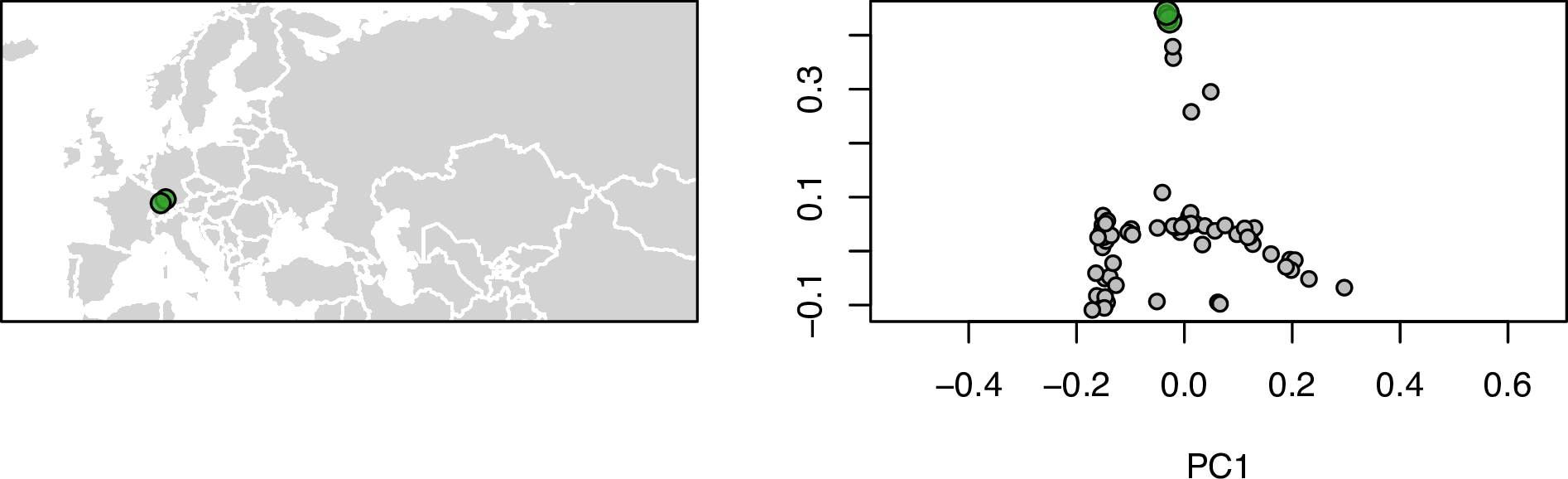

Supplement: S12 Fig — (TIF) [file pgen.1006852.s030.tif]

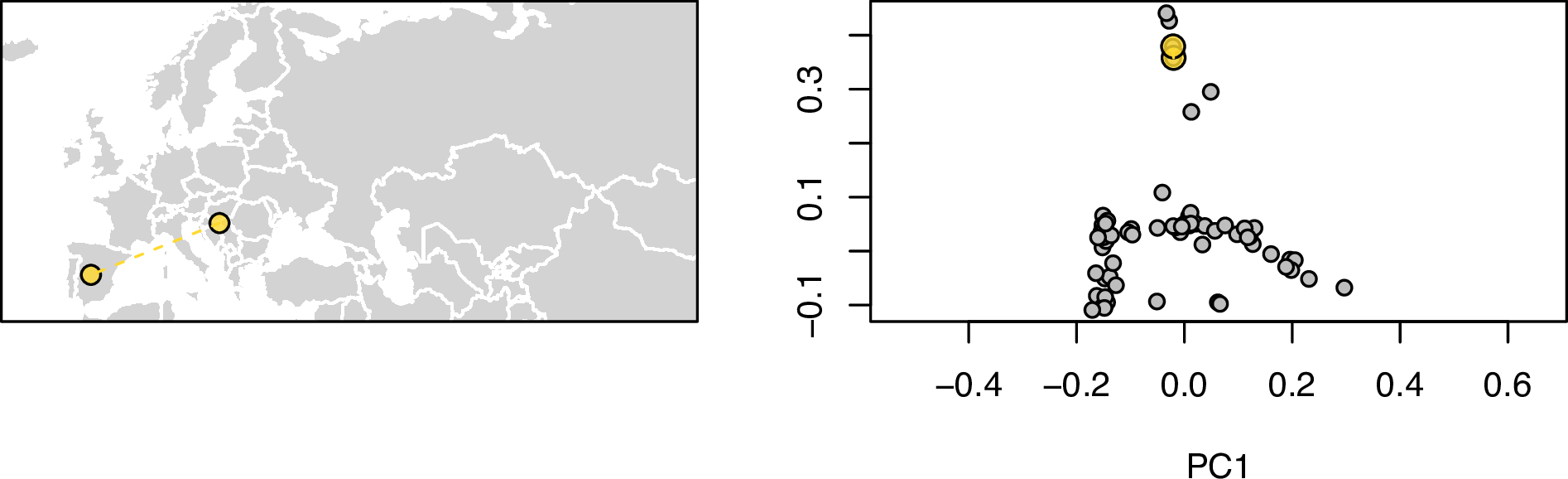

Supplement: S13 Fig — (TIF) [file pgen.1006852.s031.tif]

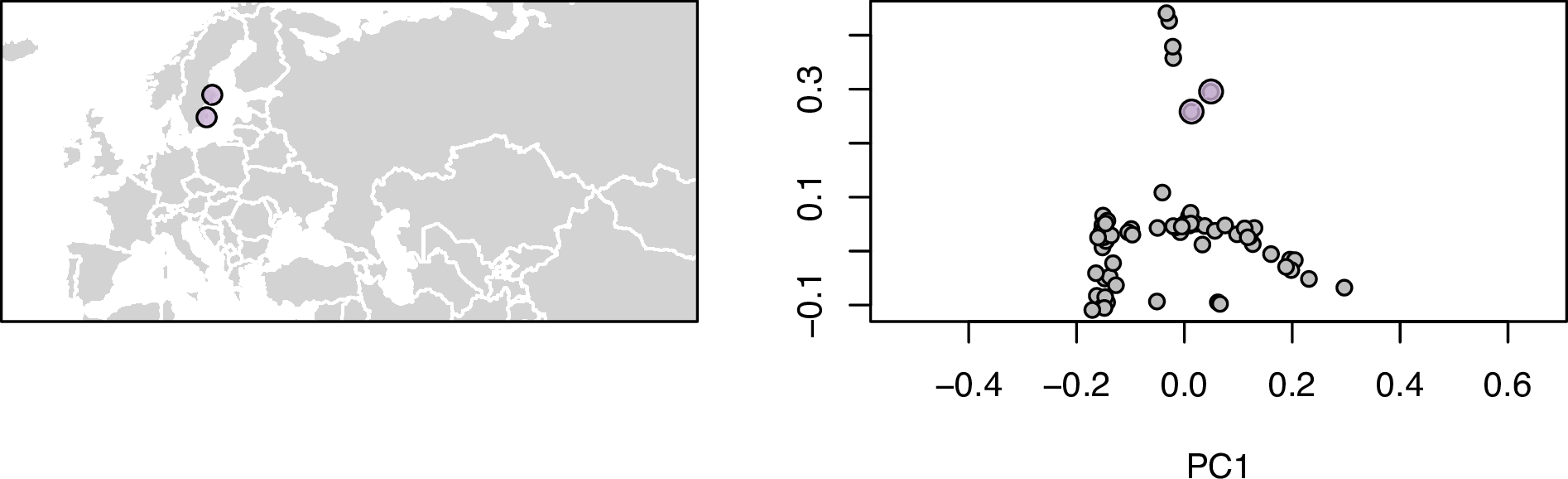

Supplement: S14 Fig — (TIF) [file pgen.1006852.s032.tif]

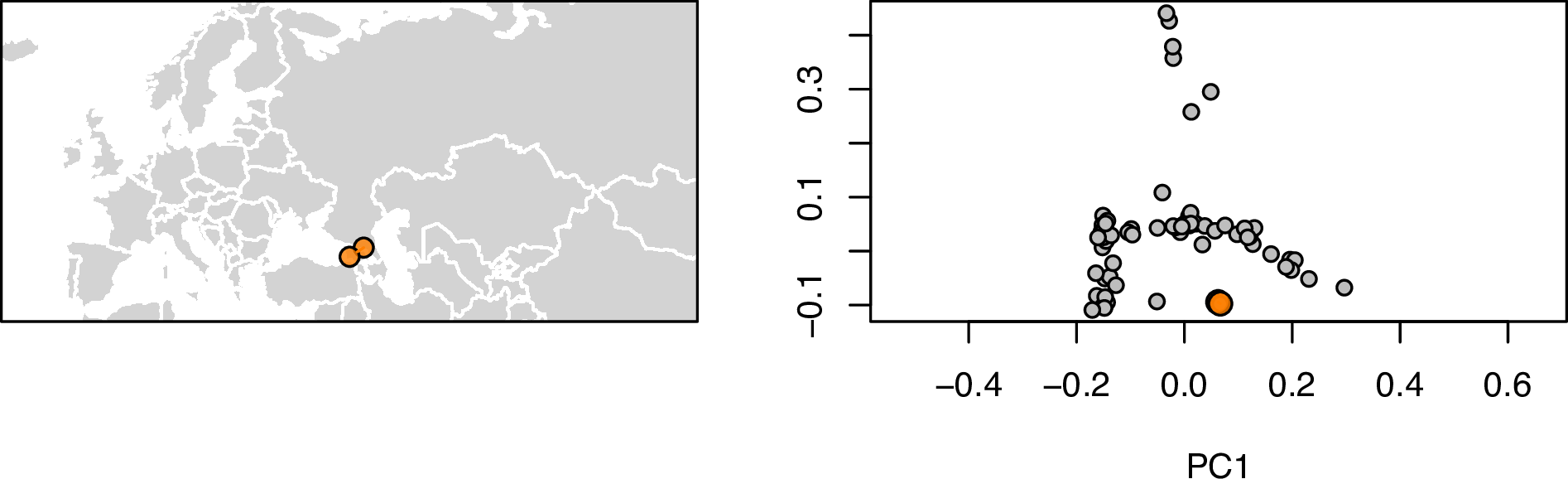

Supplement: S15 Fig — (TIF) [file pgen.1006852.s033.tif]

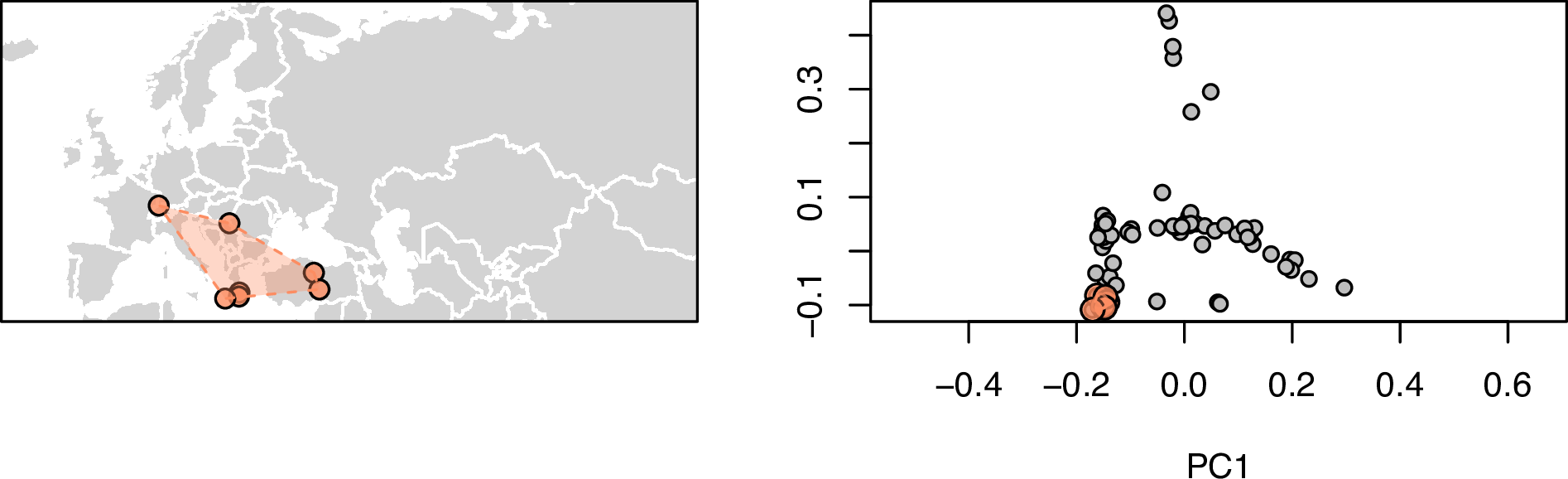

Supplement: S16 Fig — (TIF) [file pgen.1006852.s034.tif]

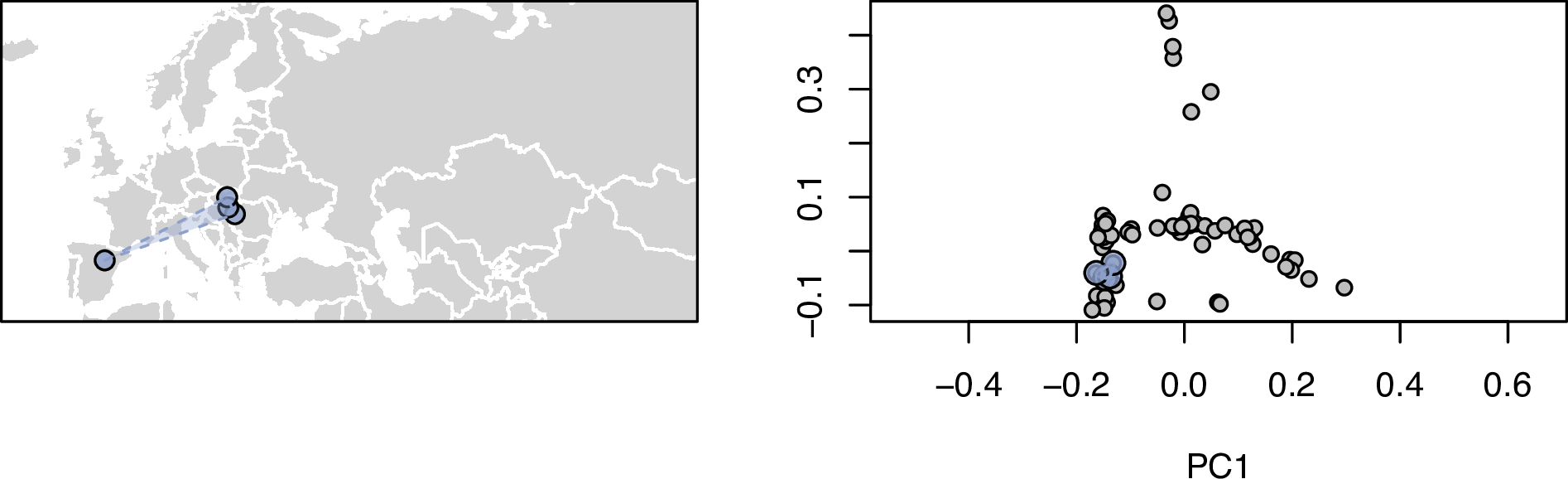

Supplement: S17 Fig — (TIF) [file pgen.1006852.s035.tif]

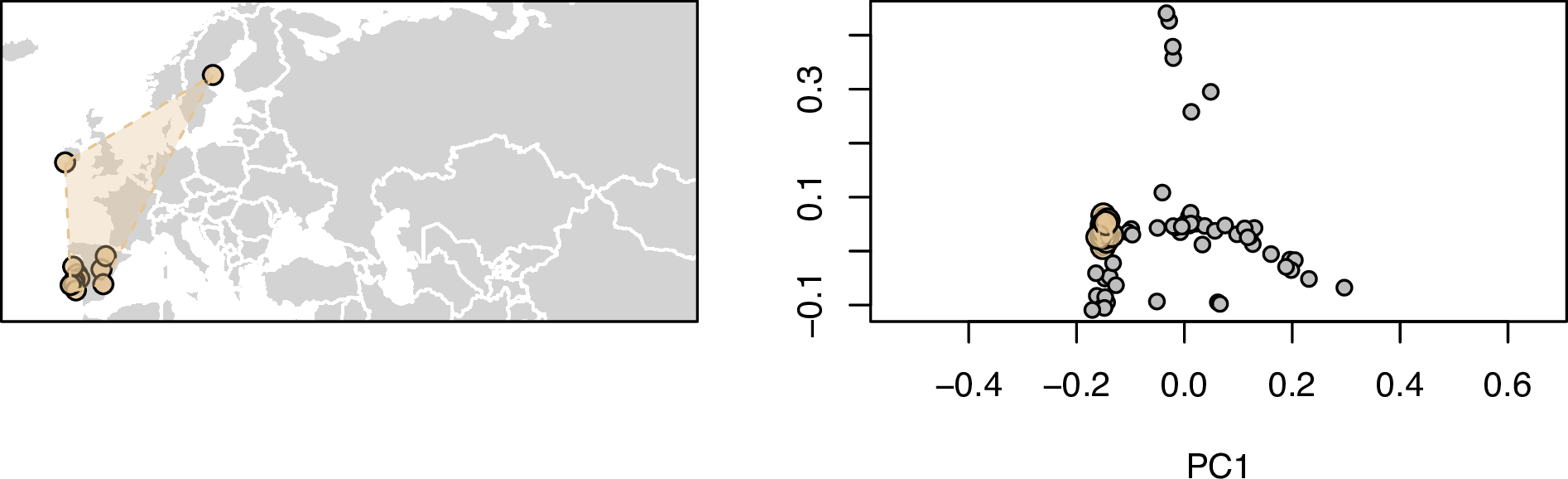

Supplement: S18 Fig — (TIF) [file pgen.1006852.s036.tif]

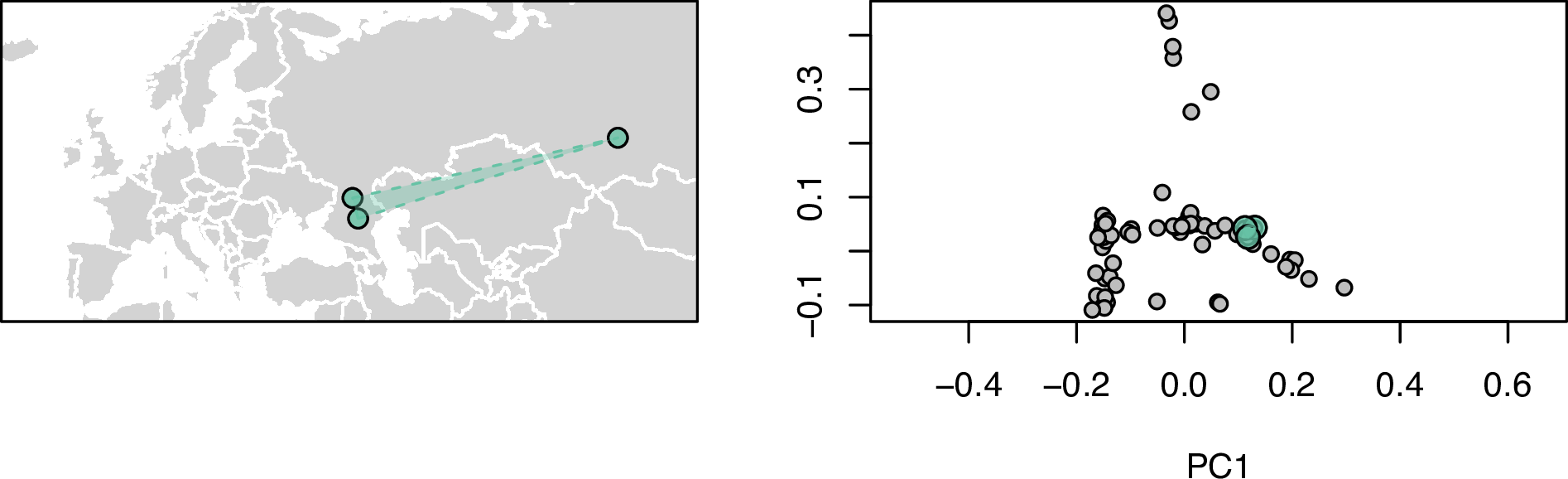

Supplement: S19 Fig — (TIF) [file pgen.1006852.s037.tif]

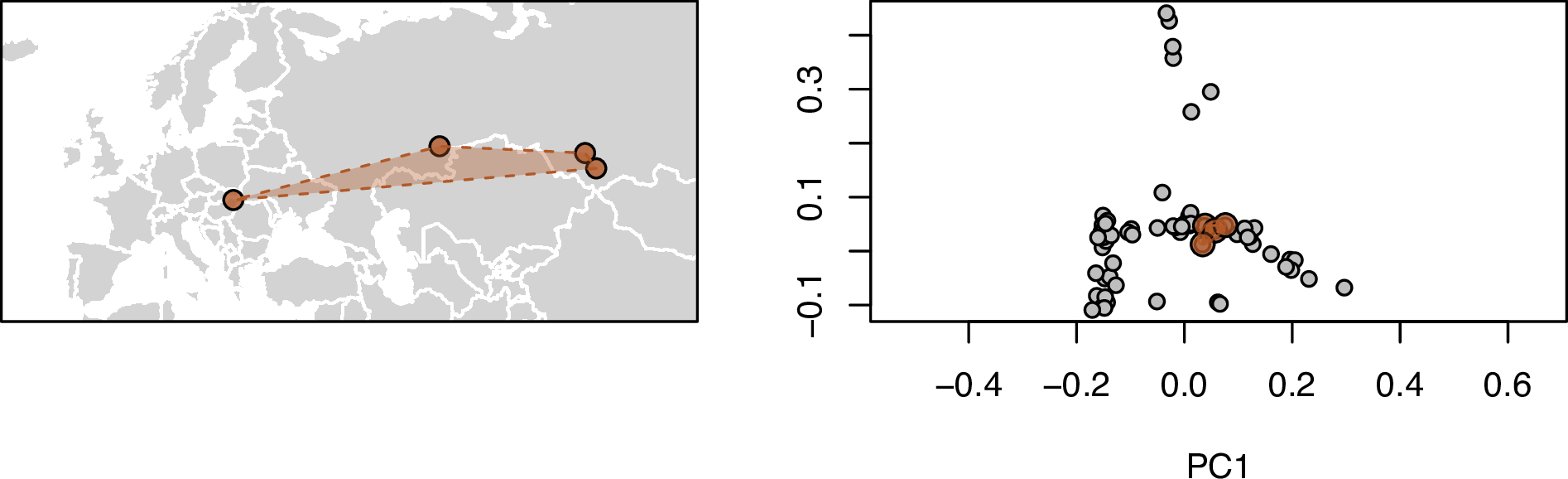

Supplement: S20 Fig — (TIF) [file pgen.1006852.s038.tif]

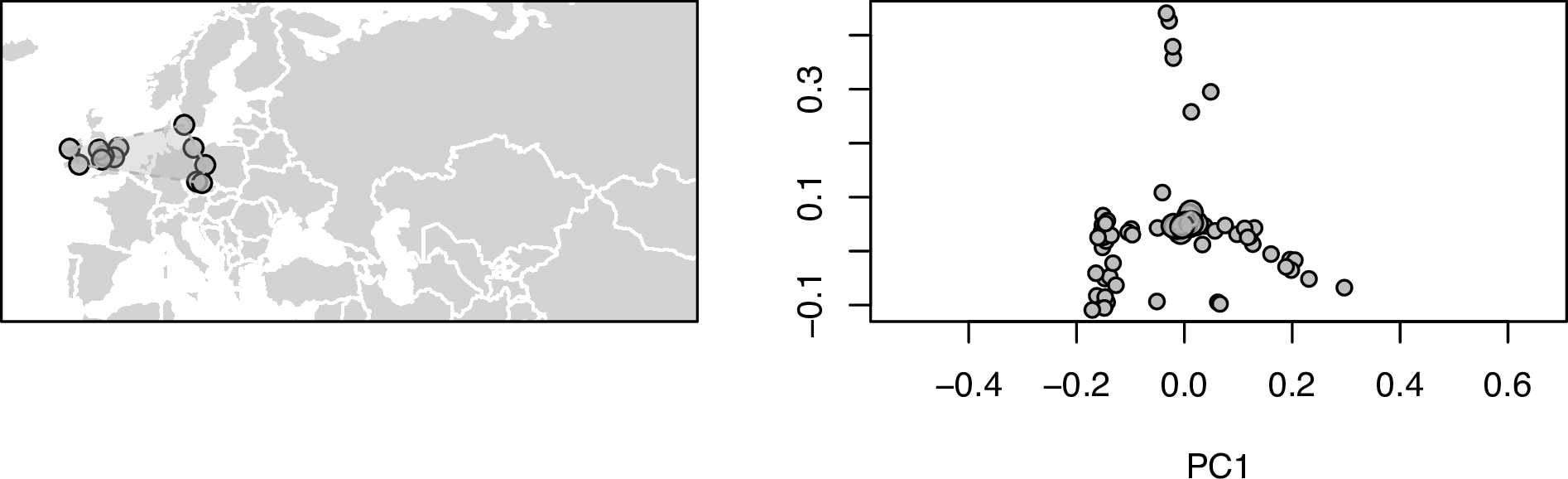

Supplement: S21 Fig — (TIF) [file pgen.1006852.s039.tif]

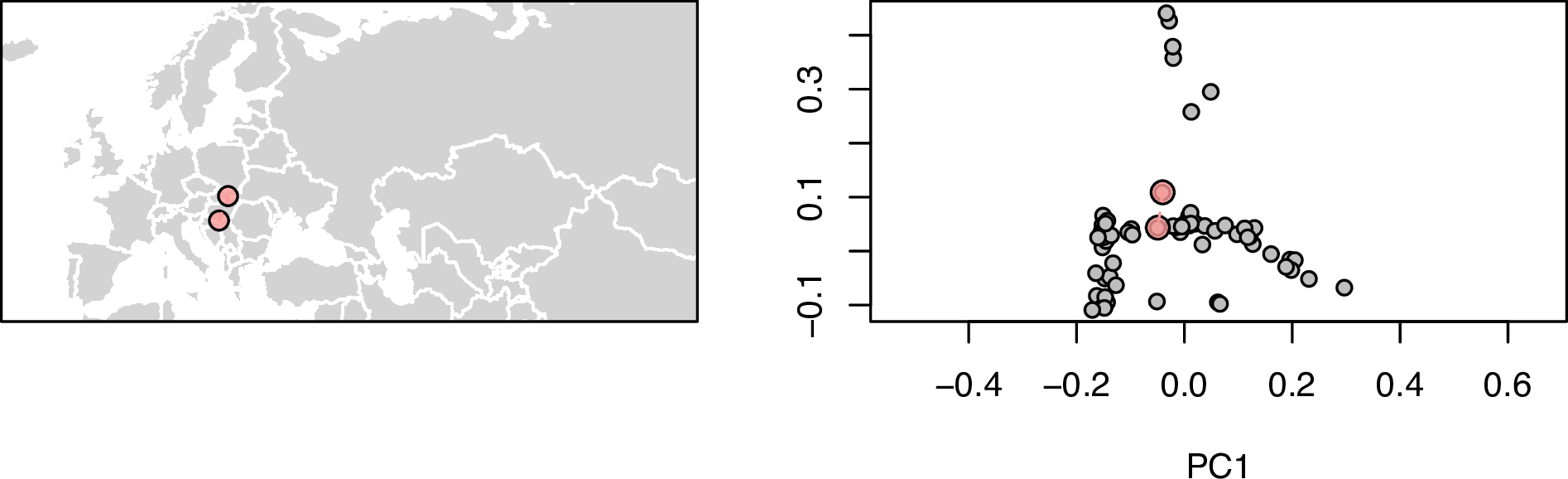

Supplement: S22 Fig — (TIF) [file pgen.1006852.s040.tif]

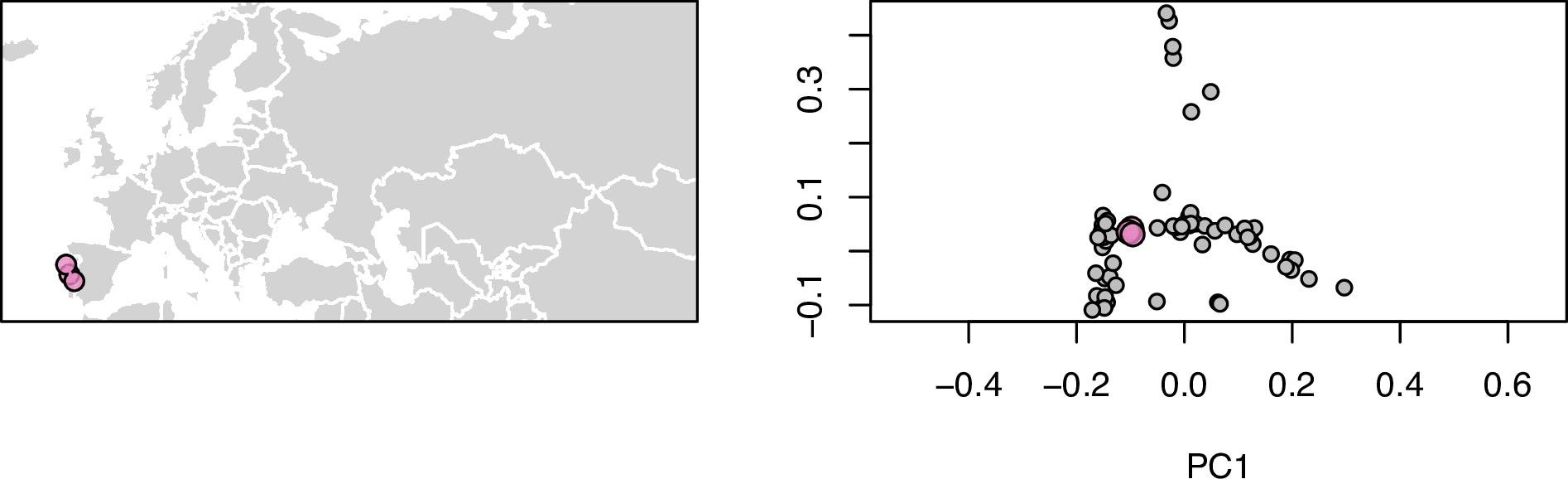

Supplement: S23 Fig — (TIF) [file pgen.1006852.s041.tif]

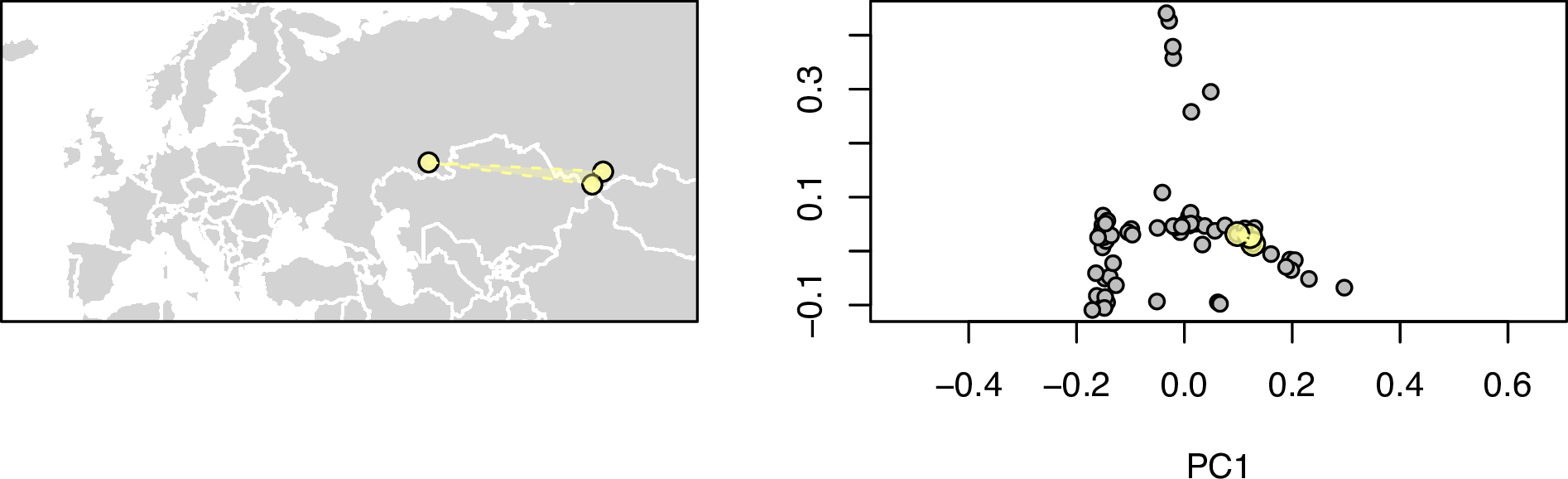

Supplement: S24 Fig — (TIF) [file pgen.1006852.s042.tif]

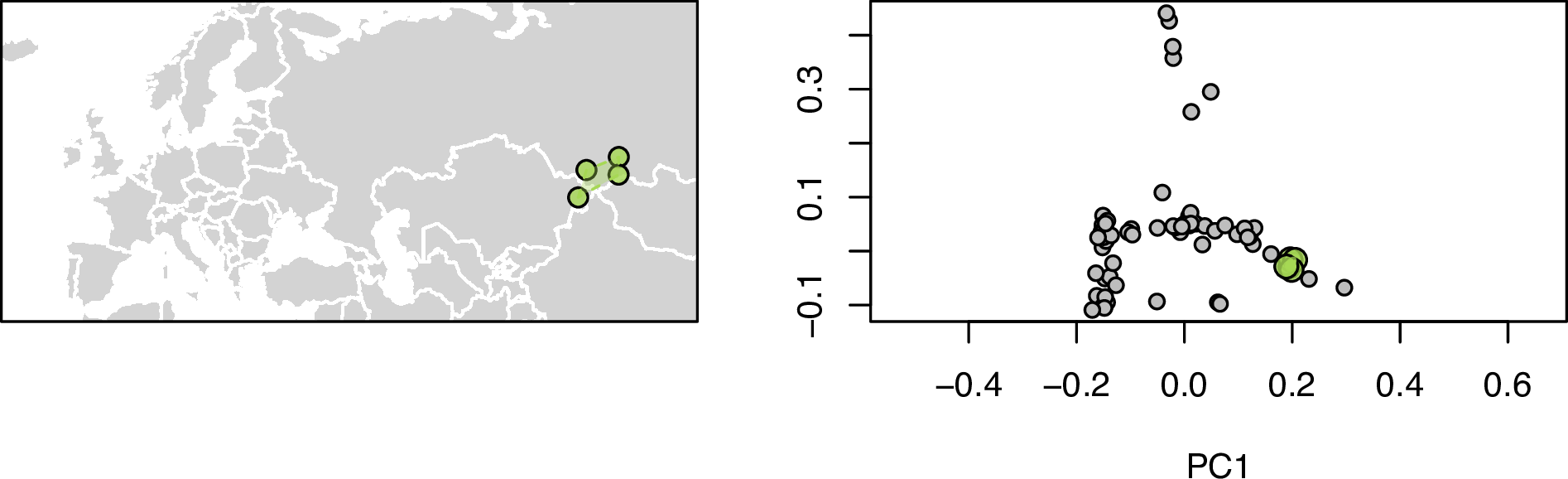

Supplement: S25 Fig — (TIF) [file pgen.1006852.s043.tif]

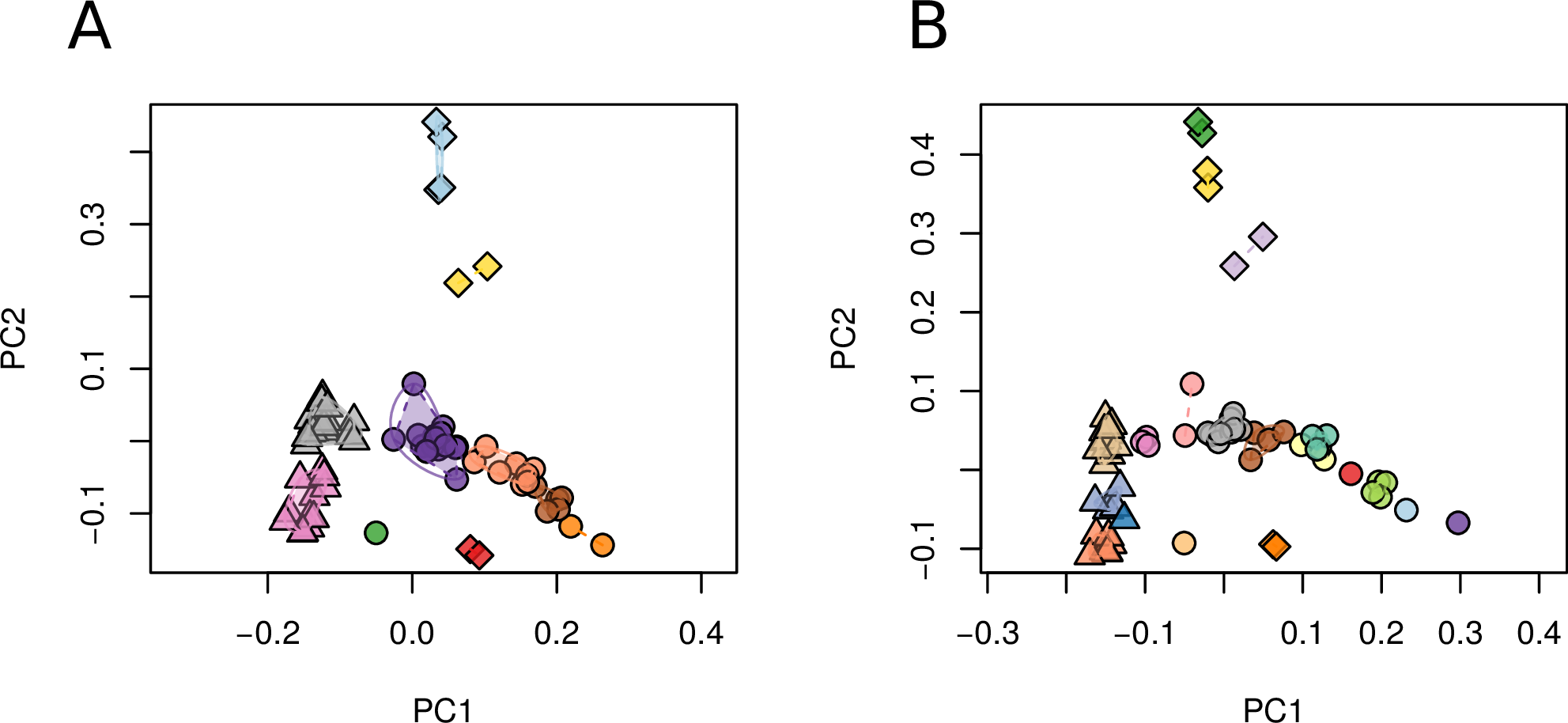

Supplement: S26 Fig — The unlinked analysis is only able to identify 10 populations, 9 less than when incorporating the linkage model. (TIF) [file pgen.1006852.s044.tif]

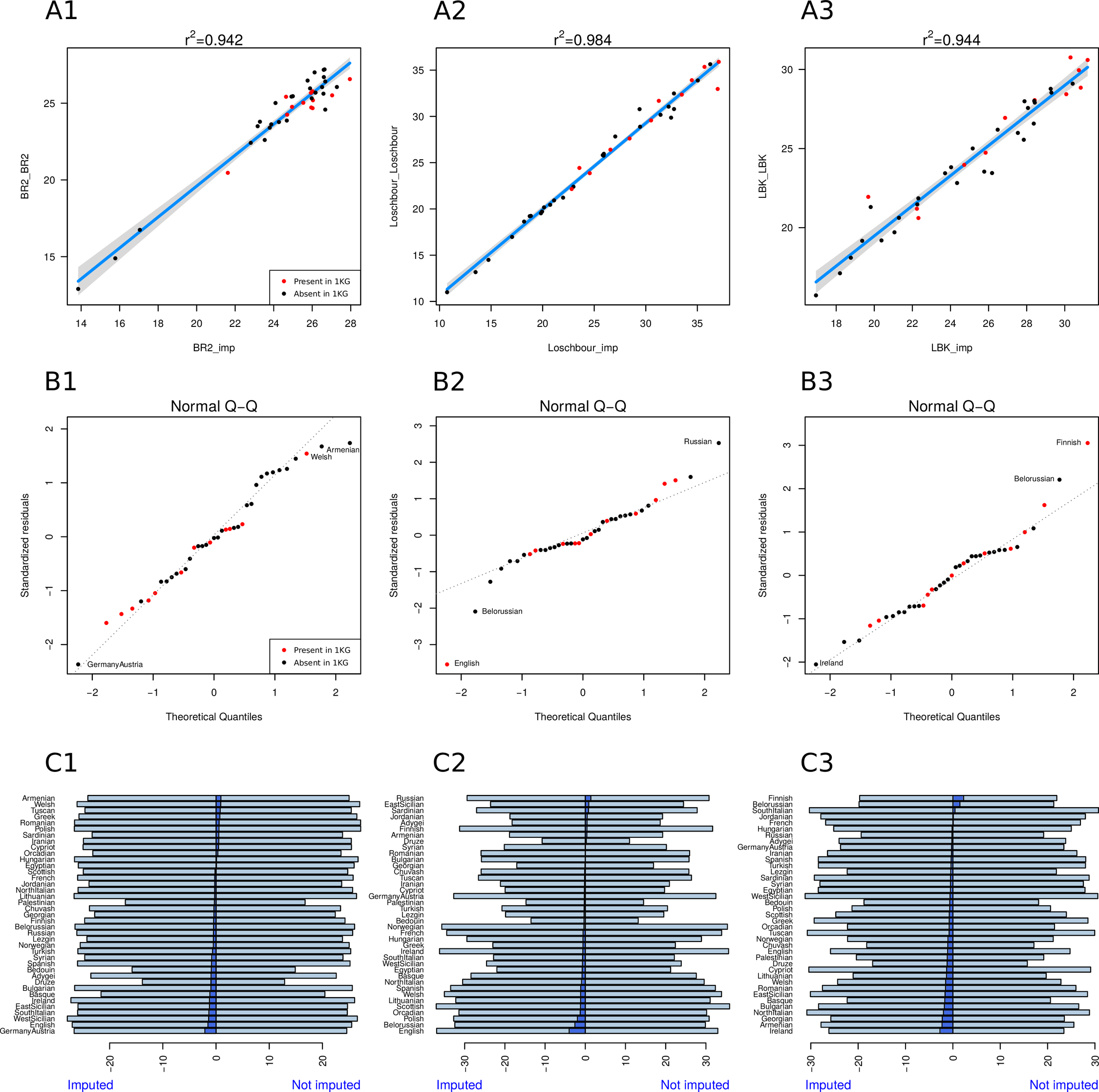

Supplement: S27 Fig — (A) Correlation between imputed and non-imputed median haplotype donation from sample BR2 (1), Loschbour (2) and LBK (3). (B) Normal Quantile-Quantile plots and outlier detection (labelled populations). Coloured dots show populations present (red) or absent (black) in the 1000 Genomes reference haplotype dataset. (C) Barplots illustrating imputed (left) and non-imputed (right) median haplotype donation (light blue) and the difference between median haplotype donation per population (dark blue). (TIF) [file pgen.1006852.s045.tif]

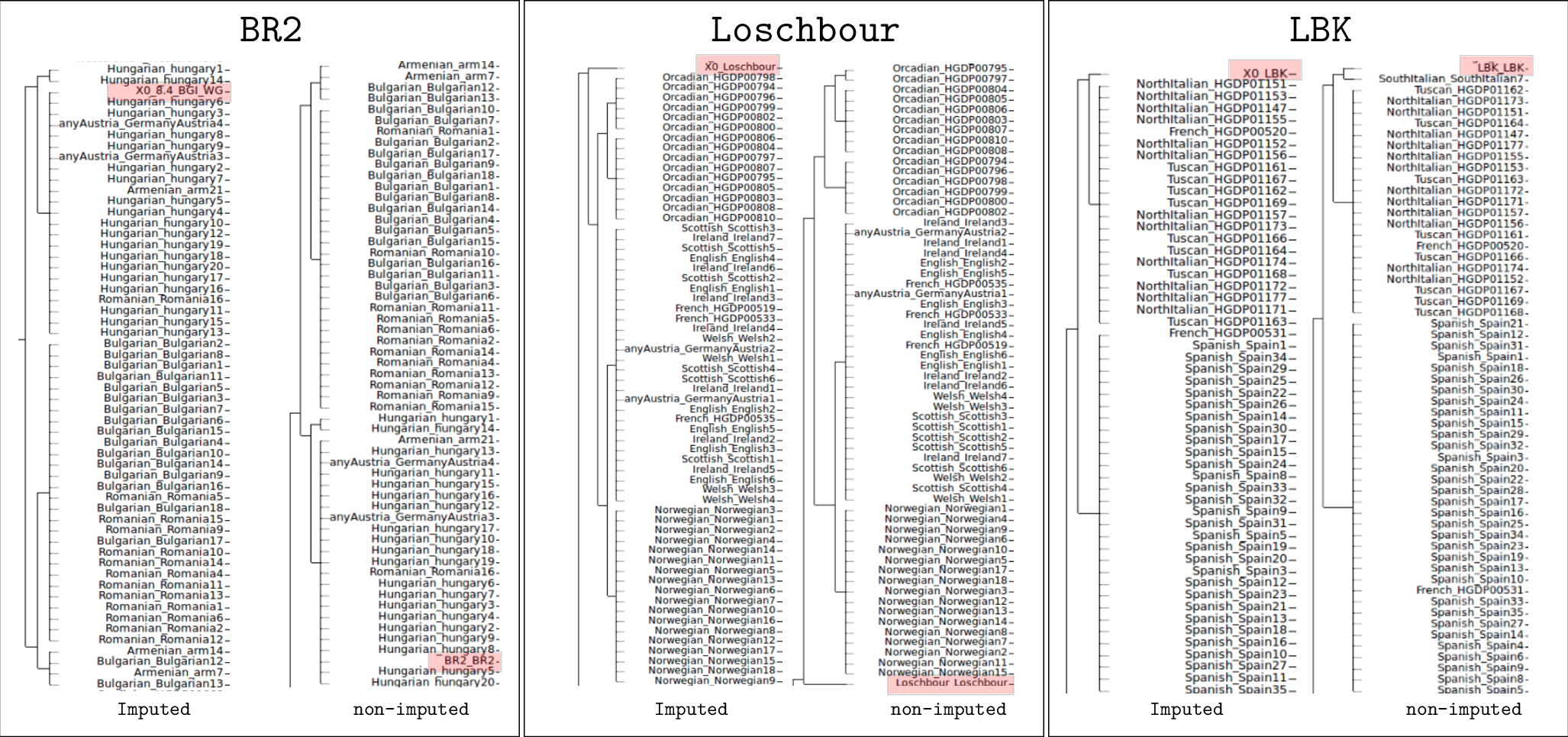

Supplement: S28 Fig — The position of aDNA samples (shown in red) is very similar in both analyses. (TIF) [file pgen.1006852.s046.tif]

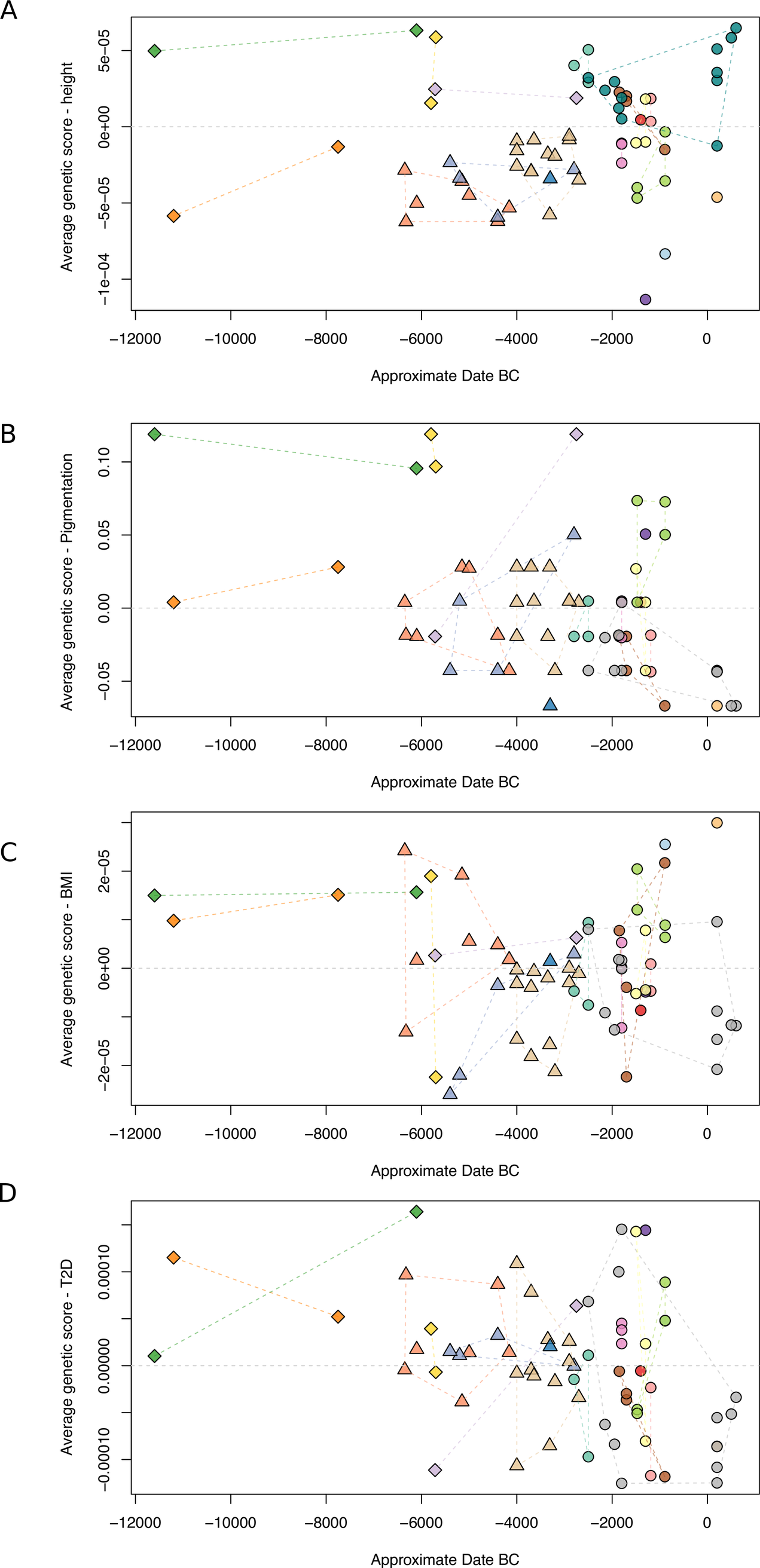

Supplement: S29 Fig — The traits chosen were: A) Height; B) Pigmentation; C) BMI and D) T2D. Polygenic scores were centered at the mean for the dataset. As in Fig 1 in the main text, each cluster is represented with a different colour. (TIF) [file pgen.1006852.s047.tif]

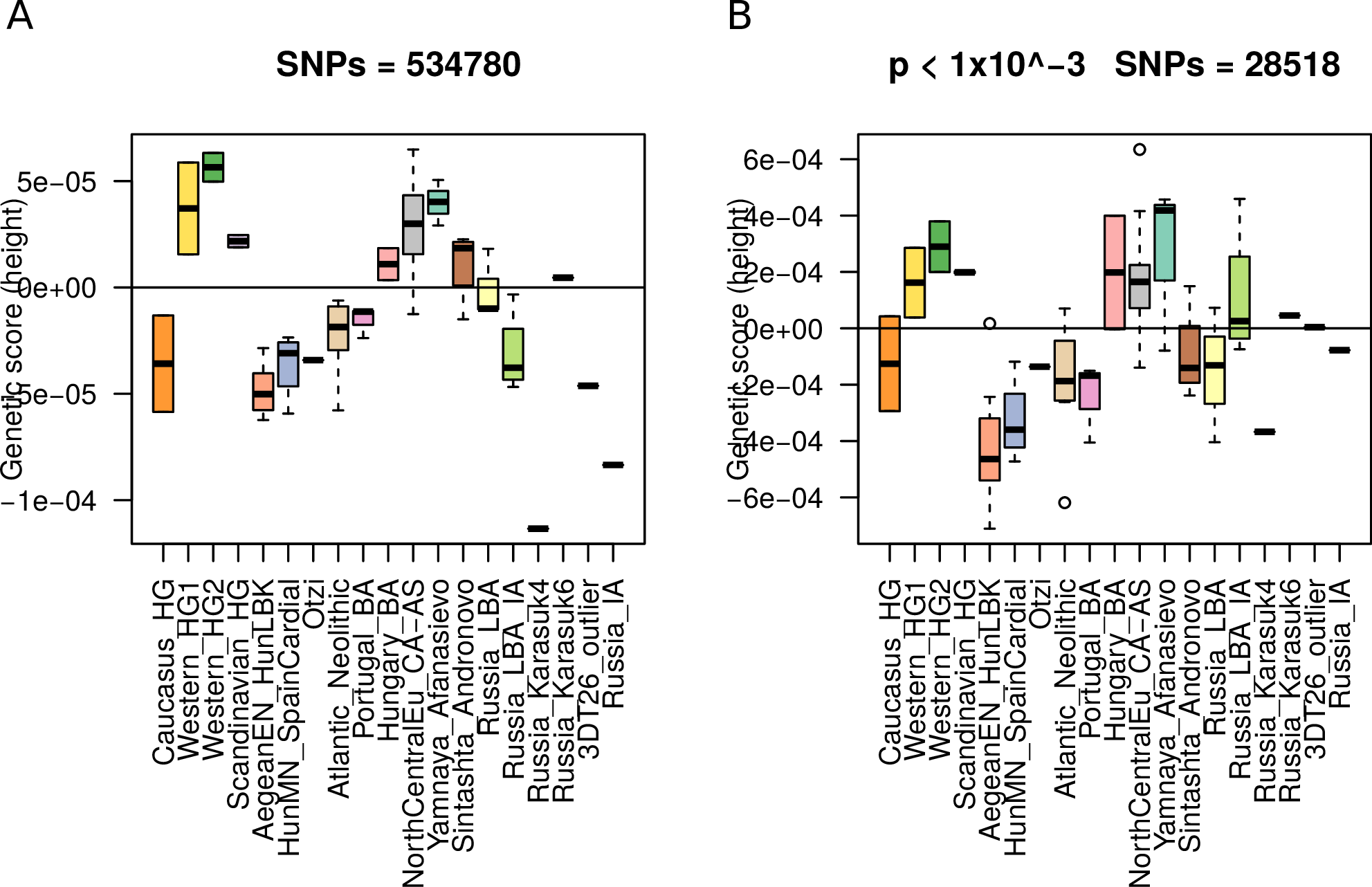

Supplement: S30 Fig — (A) p = 0 (B) p<0.001. SNPs with posterior genotype probability of less than 0.99 were excluded from analysis. (TIF) [file pgen.1006852.s048.tif]

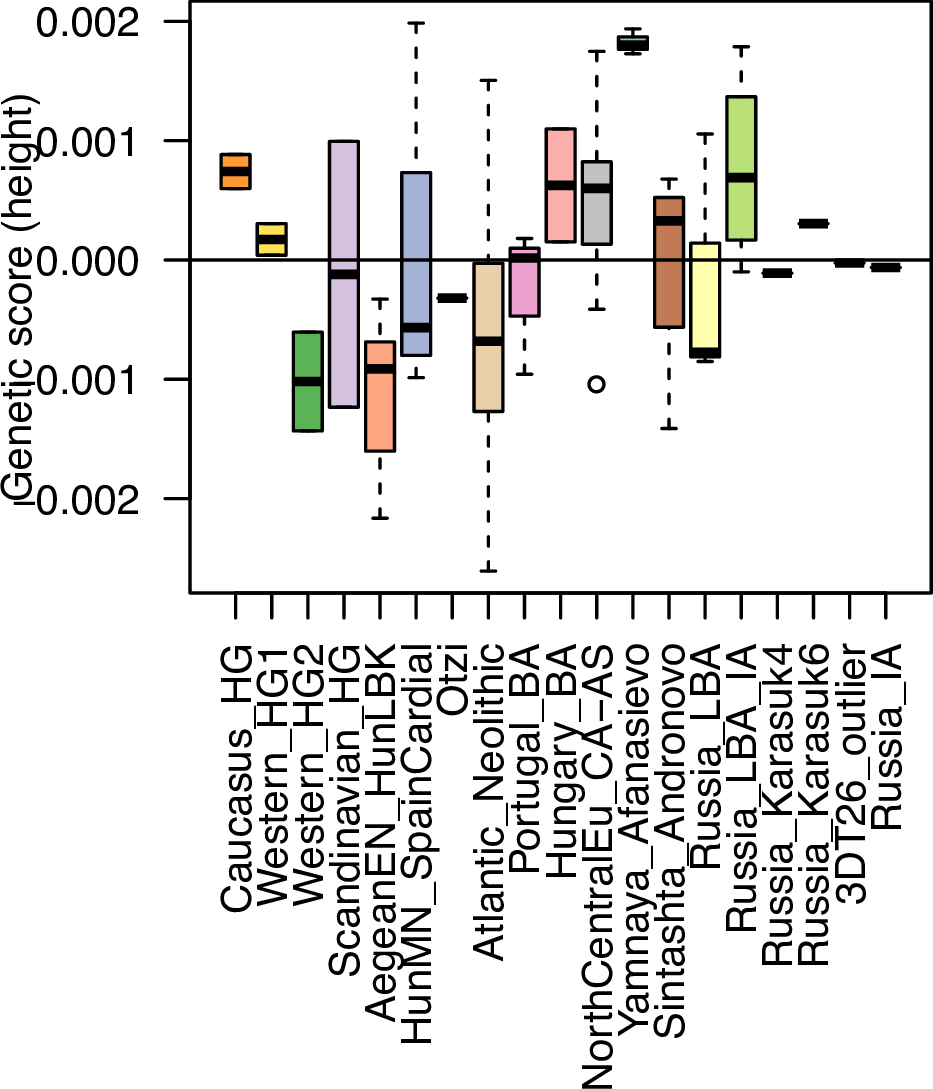

Supplement: S31 Fig — (TIF) [file pgen.1006852.s049.tif]

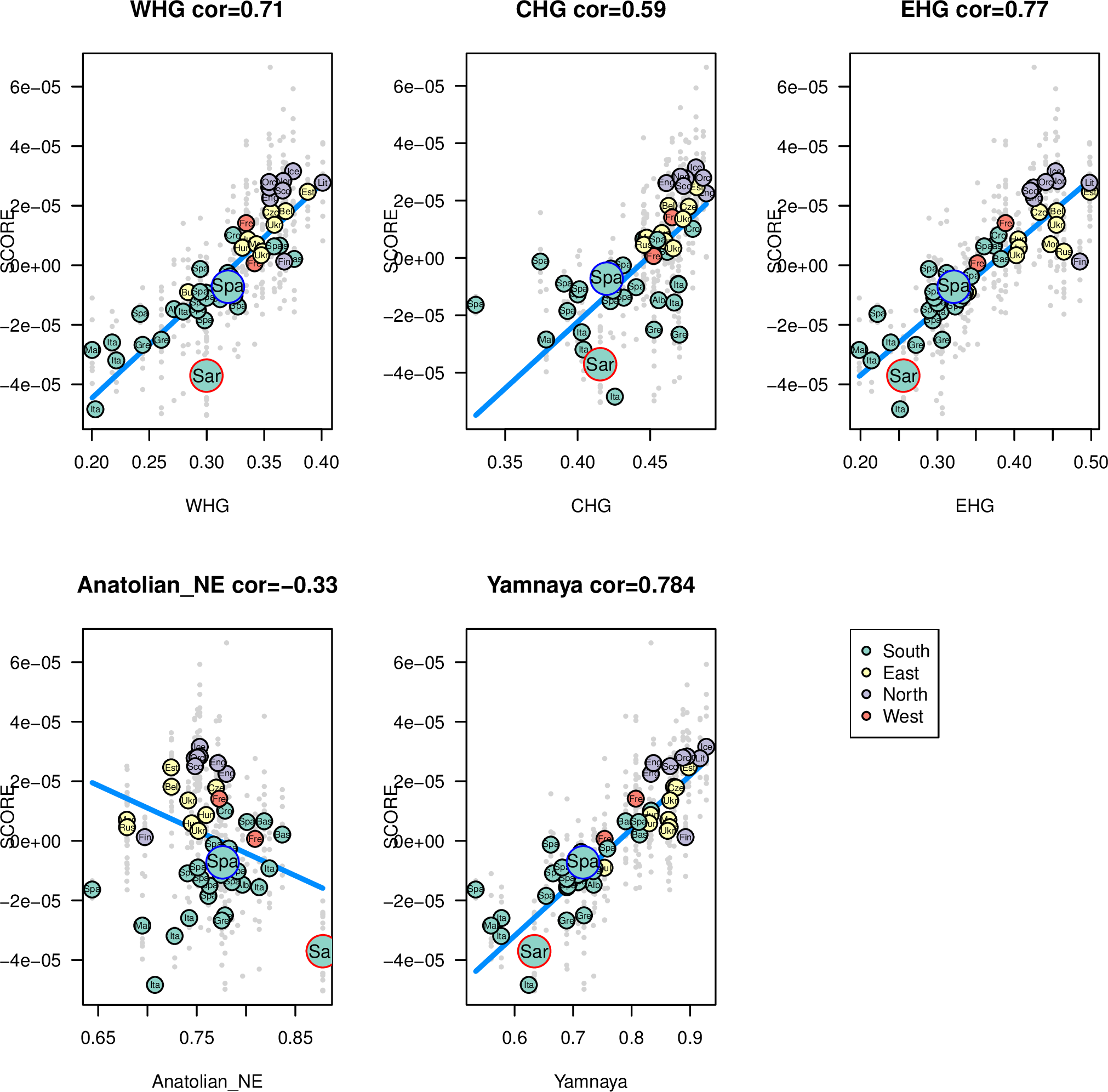

Supplement: S32 Fig — Hunter-gatherer (WHG, EHG, CHG), Neolithic (Anatolian_EN) and Steppe (Yamnaya) Ancestry was measured by f4(Mbuti, Ancient_Ind1; Modern_WEurasian, Dai)/f4(Mbuti, Ancient_Ind1; Ancient_Ind2, Dai). Polygenic risk scores for height (92) were determined using ~280.000 SNPs in 48 European populations. Blue line presents the linear regression. Individual samples are represented by gray dots and larger coloured circles represent the mean genetic score for each population. (TIF) [file pgen.1006852.s050.tif]

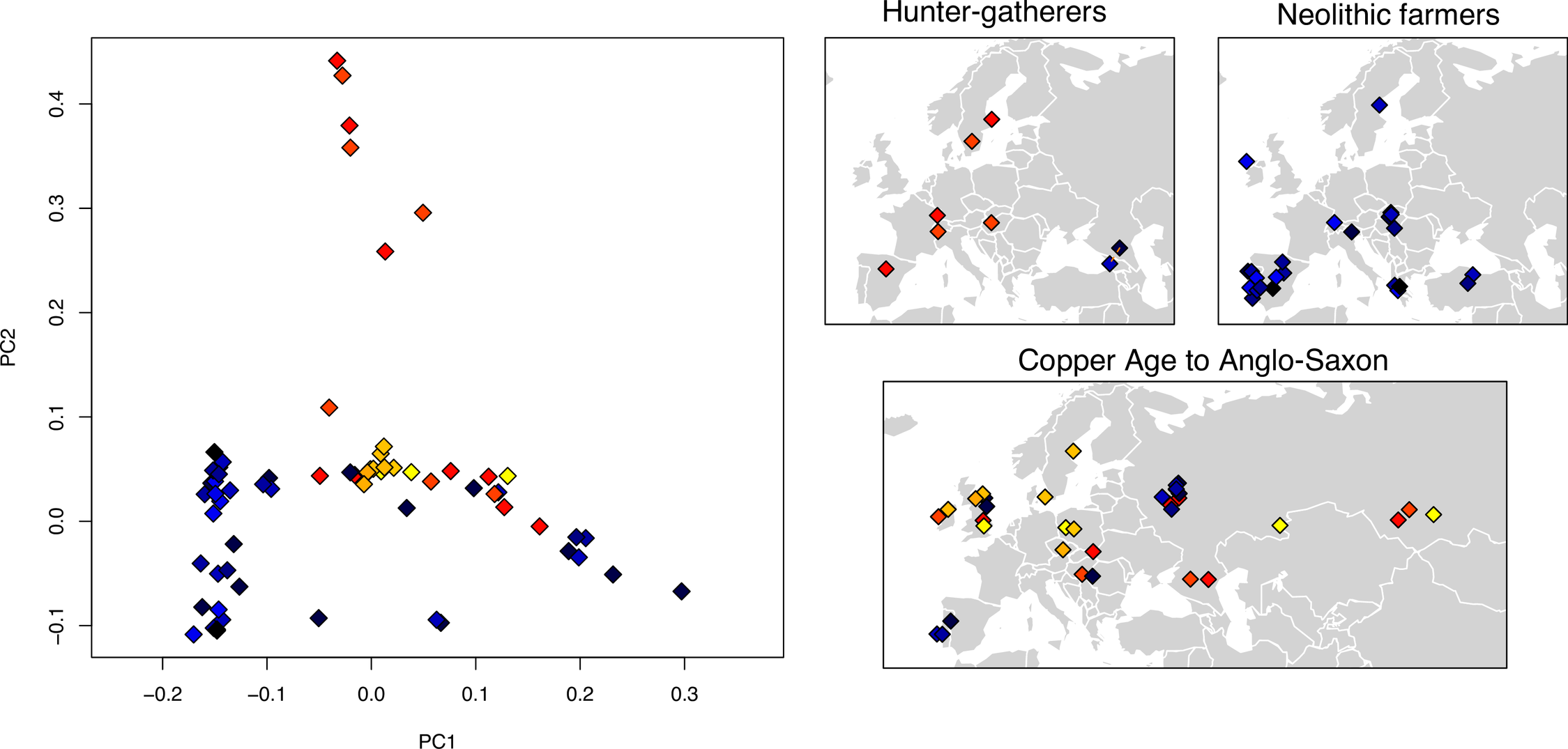

Supplement: S33 Fig — Red—increased genetic height scores, black—decreased genetic height. Broadly, hunter-gatherers and populations from Copper age and after present highest proportion of height increasing associated variants followed by Neolithic farmers. (TIF) [file pgen.1006852.s051.tif]

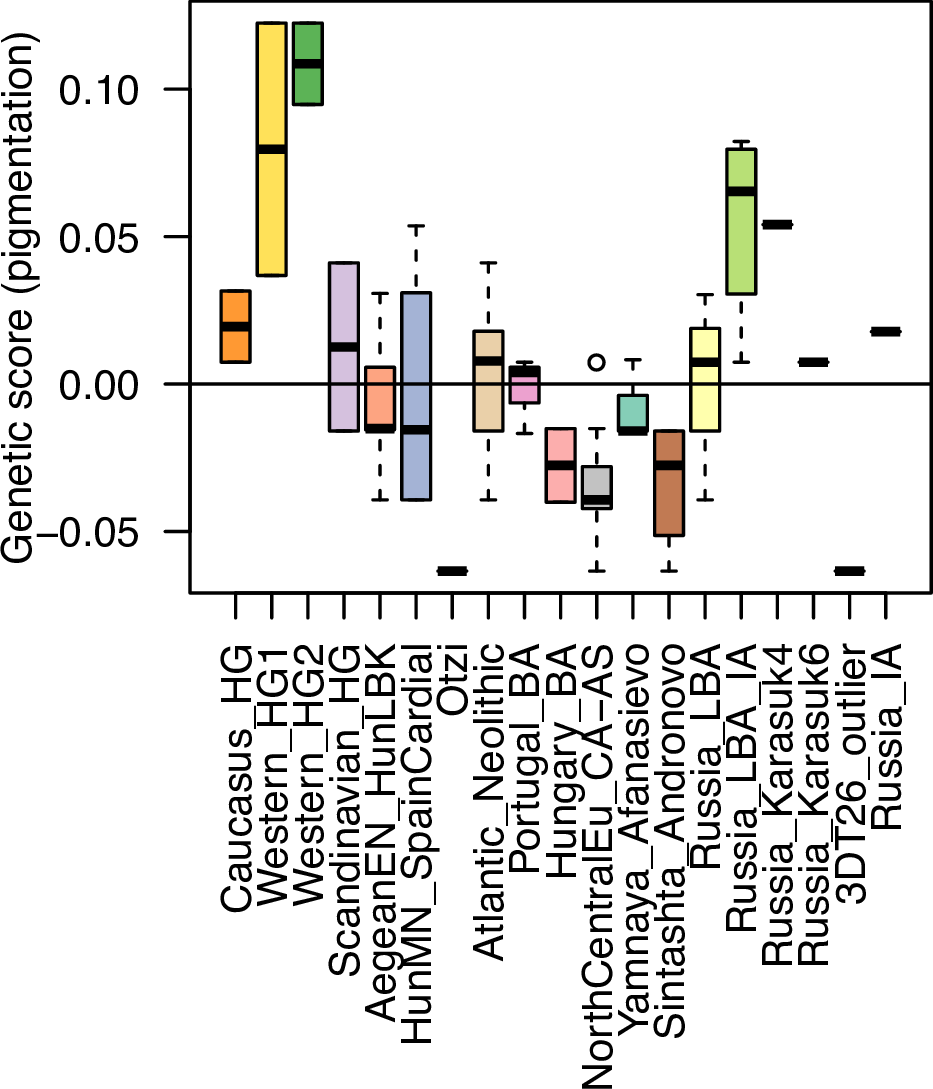

Supplement: S34 Fig — SNPs with posterior genotype probability of less than 0.99 were excluded from analysis. (TIF) [file pgen.1006852.s052.tif]

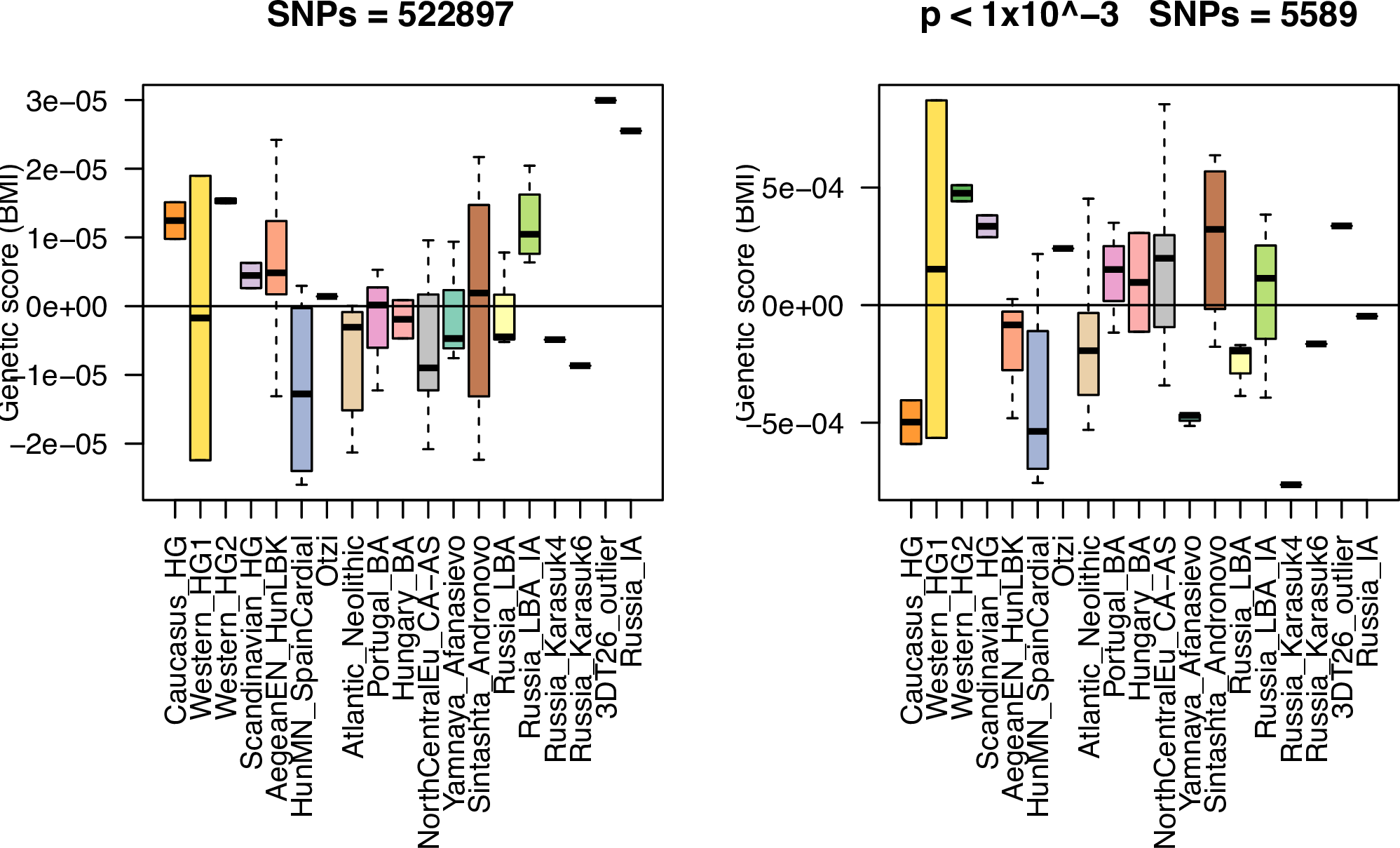

Supplement: S35 Fig — (A) p = 0 (B) p<0.001. SNPs with posterior genotype probability of less than 0.99 were excluded from analysis. (TIF) [file pgen.1006852.s053.tif]

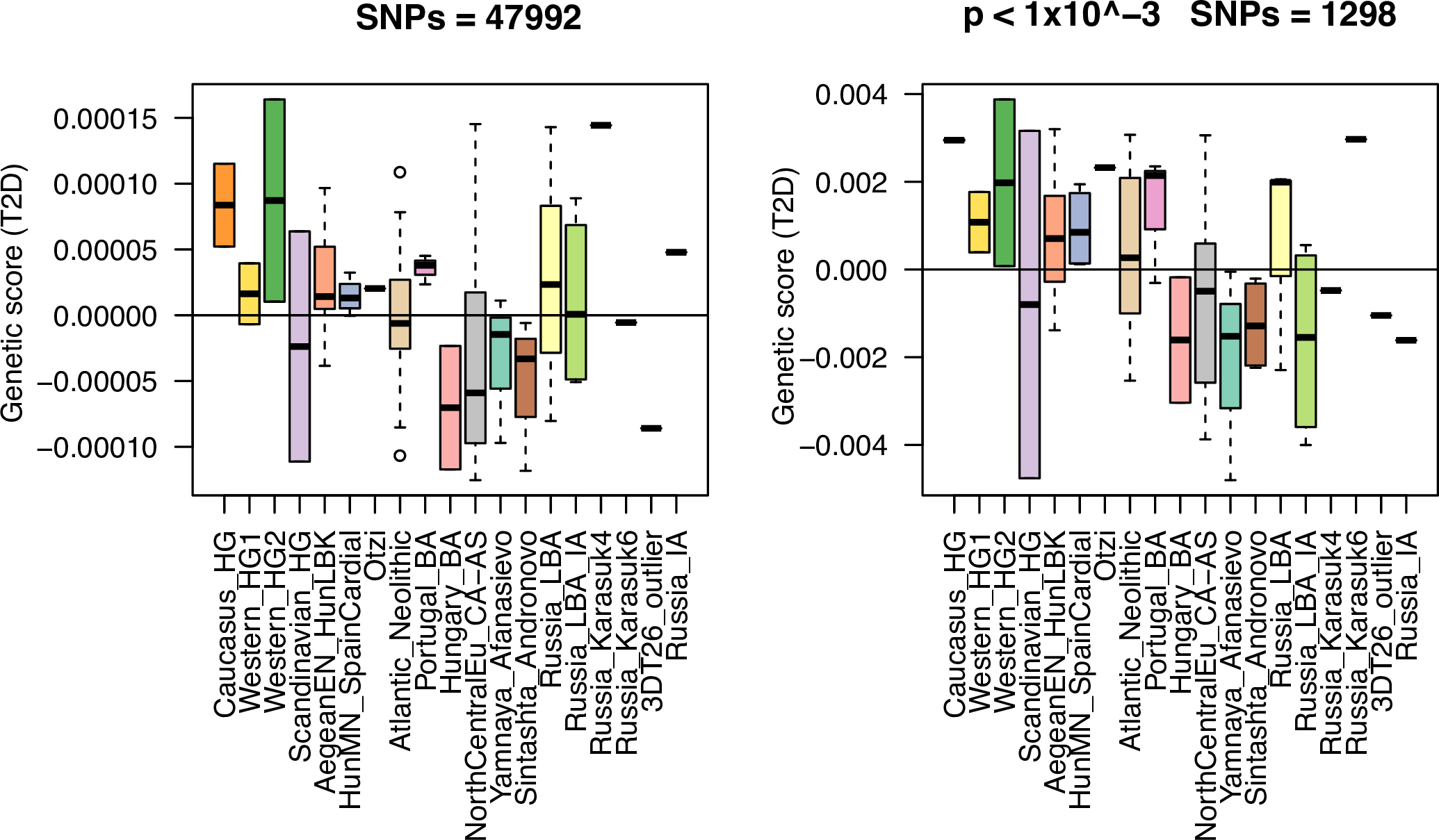

Supplement: S36 Fig — A) p = 0 B) p<0.001. SNPs with posterior genotype probability of less than 0.99 were excluded from analysis. (TIF) [file pgen.1006852.s054.tif]

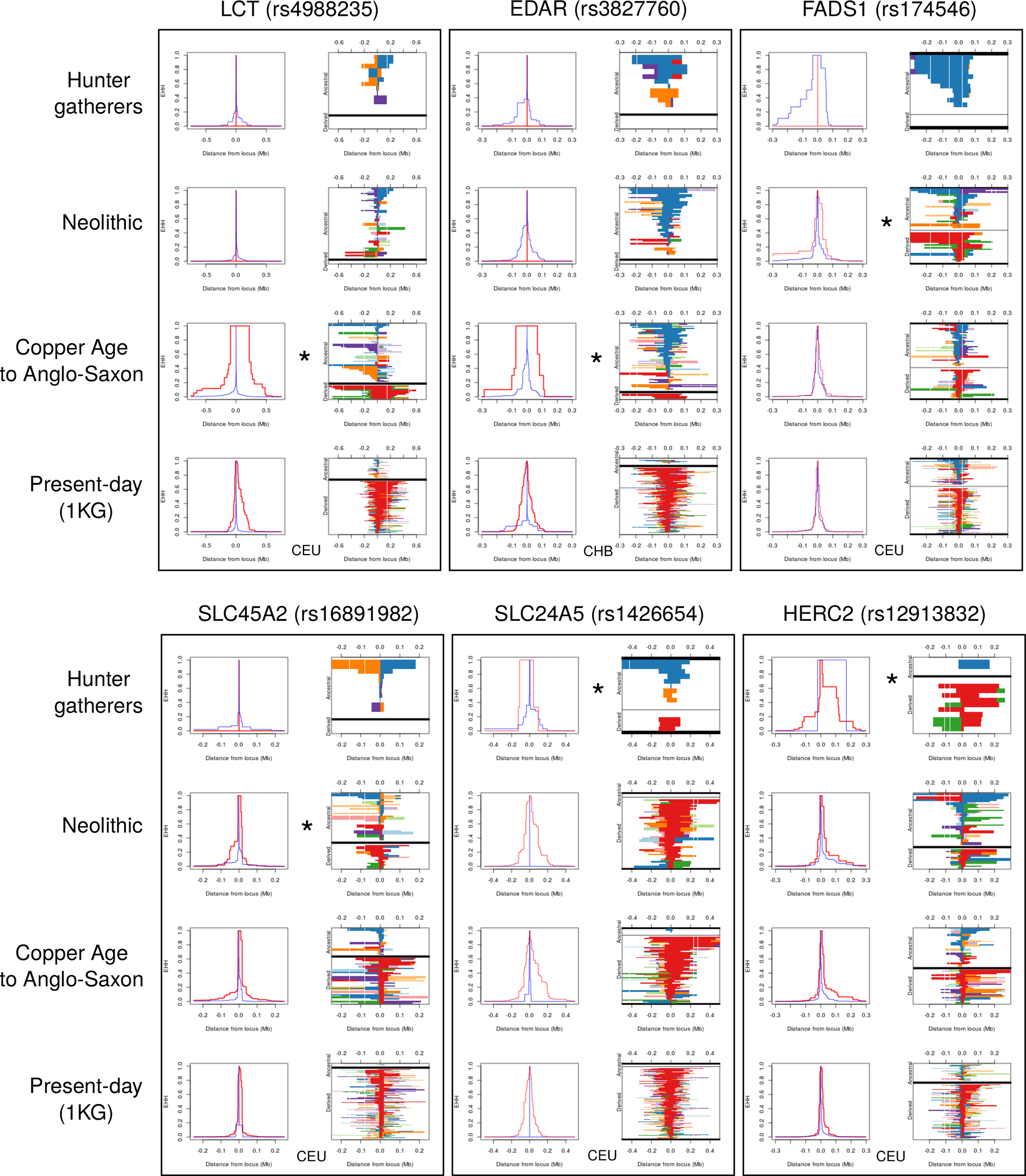

Supplement: S37 Fig — Panels on the left represent the decay of EHH, or the probability of homozygosity at a certain base across 2 randomly chosen chromosomes in a population. Plots on the right represent existing haplotypes in a population, with the lower portion of the graph depicting haplotypes with the derived allele (red) and the upper part showing haplotypes carrying the ancestral allele (blue). Unique haplotypes in a population are not represented. Legend: CEU—Utah Residents (CEPH) with Northern and Western Ancestry; YRI—Yoruba in Ibadan, Nigeria; CHB—Han Chinese in Beijing, China; 1KG: 1000 Genomes Project. * Earliest appearance of the homozygous derived allele in the samples analysed. (TIF) [file pgen.1006852.s055.tif]
